# A modular two yeast species secretion system for the production and preparative application of unspecific peroxygenases

**DOI:** 10.1038/s42003-021-02076-3

**Published:** 2021-05-12

**Authors:** Pascal Püllmann, Anja Knorrscheidt, Judith Münch, Paul R. Palme, Wolfgang Hoehenwarter, Sylvestre Marillonnet, Miguel Alcalde, Bernhard Westermann, Martin J. Weissenborn

**Affiliations:** 1 Leibniz Institute of Plant Biochemistry, Halle (Saale), Germany; 2 Department of Biocatalysis, Institute of Catalysis, CSIC, Madrid, Spain; 3 Institute of Chemistry, Martin-Luther-University Halle-Wittenberg, Halle (Saale), Germany

**Keywords:** Oxidoreductases, Expression systems, Molecular biology

## Abstract

Fungal unspecific peroxygenases (UPOs) represent an enzyme class catalysing versatile oxyfunctionalisation reactions on a broad substrate scope. They are occurring as secreted, glycosylated proteins bearing a haem-thiolate active site and rely on hydrogen peroxide as the oxygen source. However, their heterologous production in a fast-growing organism suitable for high throughput screening has only succeeded once—enabled by an intensive directed evolution campaign. We developed and applied a modular Golden Gate-based secretion system, allowing the first production of four active UPOs in yeast, their one-step purification and application in an enantioselective conversion on a preparative scale. The Golden Gate setup was designed to be universally applicable and consists of the three module types: i) signal peptides for secretion, ii) UPO genes, and iii) protein tags for purification and split-GFP detection. The modular episomal system is suitable for use in *Saccharomyces cerevisiae* and was transferred to episomal and chromosomally integrated expression cassettes in *Pichia pastoris*. Shake flask productions in *Pichia pastoris* yielded up to 24 mg/L secreted UPO enzyme, which was employed for the preparative scale conversion of a phenethylamine derivative reaching 98.6 % *ee*. Our results demonstrate a rapid, modular yeast secretion workflow of UPOs yielding preparative scale enantioselective biotransformations.

## Abstract (web summary)

Püllmann et al developed a modular Golden Gate-based secretion system, which enabled production and one-step purification of active fungal unspecific peroxygenases (UPOs) in yeast. Their system was applied to an enantioselective conversion on a preparative scale and may be used in the future for other genes of interest that are suitable for production in yeast.

## Introduction

Fungal unspecific peroxygenases (UPOs) have recently emerged as novel versatile hydroxylation biocatalysts. They solely rely on hydrogen peroxide as cosubstrate reaching impressive total turnover numbers for sp^3^-carbon hydroxylation of up to 300000^1–4^. Further UPO catalysed reactions include aromatic hydroxylation, heteroatom oxidation, halogenation and carbon–carbon double bond epoxidation^5^. Due to their high oxyfunctionalisation versatility and activity, while solely requiring hydrogen peroxide and being independent of auxiliary electron transport proteins and expensive cofactors, UPOs have attracted keen interest in the biocatalysis field^5–7^. Current challenges include suboptimal regio- and enantioselectivities towards specific substrates and the extremely limited panel of available UPOs, impeding broader screening setups to find a suitable catalyst to perform a particular reaction. There is an estimated number of more than 4000 putative UPO genes currently annotated and widely spread within the fungal kingdom representing just a small fraction of the available genetic diversity^8^.

To provide further insight into the natural function of UPOs as well as broadening the available substrate scope, it is crucial to access more enzymes from diverse phylogenetic backgrounds. Recent studies reported on the conversion of testosterone by a UPO derived from an ascomycetous mould, a reaction that could not be performed by any other UPO thus far^9^. This new activity further emphasises the limitations of the small available UPO panel. It would be further highly desirable to heterologously produce UPOs utilising fast-growing standard laboratory hosts such as bacteria or yeast. These organisms would facilitate protein engineering and allow directed evolution campaigns for tailoring UPOs towards desirable traits such as increased stability, regio- and enantioselectivity.

Although substantial work has been invested into the heterologous expression of the firstly discovered *Agrocybe aegerita* UPO (*Aae*UPO) using the yeast *Saccharomyces cerevisiae*, sufficient protein amounts of 8 mg/L were obtained as the result of an intensive directed evolution campaign^10^. This fundamental work led to several successful UPO application studies on the conversion of a range of substrates from agrochemicals to pharmaceuticals^11–14^. The yeast secretion variant PaDa-I (hereinafter: *AaeU*PO*) was adapted subsequently for large-scale protein production by utilising the methylotrophic yeast *Pichia pastoris* (syn. *Komagataella phaffii*) reaching recombinant protein titres of 217 mg/L within a bioreactor setup^15^.

The successful production was achieved by the introduction of nine amino acid exchanges. Four of these were localised within the 43 amino acid signal peptide (SP), which orchestrates protein secretion in the natural fungal host as well as in *S. cerevisiae*. The engineered signal peptide combined with the wild-type *Aae*UPO enzyme resulted in a 27-fold increase in protein secretion yield highlighting the paramount importance of the respective signal peptide for heterologous production as already shown by others^16–21^. Recent studies report the production of UPOs in *E. coli*^22,23^. However, it remains elusive whether these recombinant peroxygenases harbour comparable activities and stabilities if compared to UPOs produced in eukaryotic hosts. The reported expression yields are substantially lower compared to *S. cerevisiae* raising the question, whether enough functional protein could be produced for laboratory evolution campaigns.

Golden Gate cloning has proven to be an invaluable synthetic biology tool enabling seamless assembly of gene fragments utilising type IIs restriction enzymes^24–32^. By using type IIs restriction enzymes, defined 4 base pair sticky overhangs can be created for reassembly. These overhangs can be easily specified by PCR, allowing a sequence defined, efficient and seamless assembly of nine and more gene fragments in a one-pot and one-step digestion-ligation manner^25,32,33^.

For the detection of the target protein secretion in small volumetric amounts of yeast supernatant, a sensitive, high-throughput suitable, and protein-specific assay would be highly beneficial. Previously reported split-GFP (green fluorescent protein) systems, which rely on tagging the protein of interest with a short amino acid peptide tag and subsequent GFP reconstitution, present an ideal tool for this task^34,35^.

**Fig. 1:**
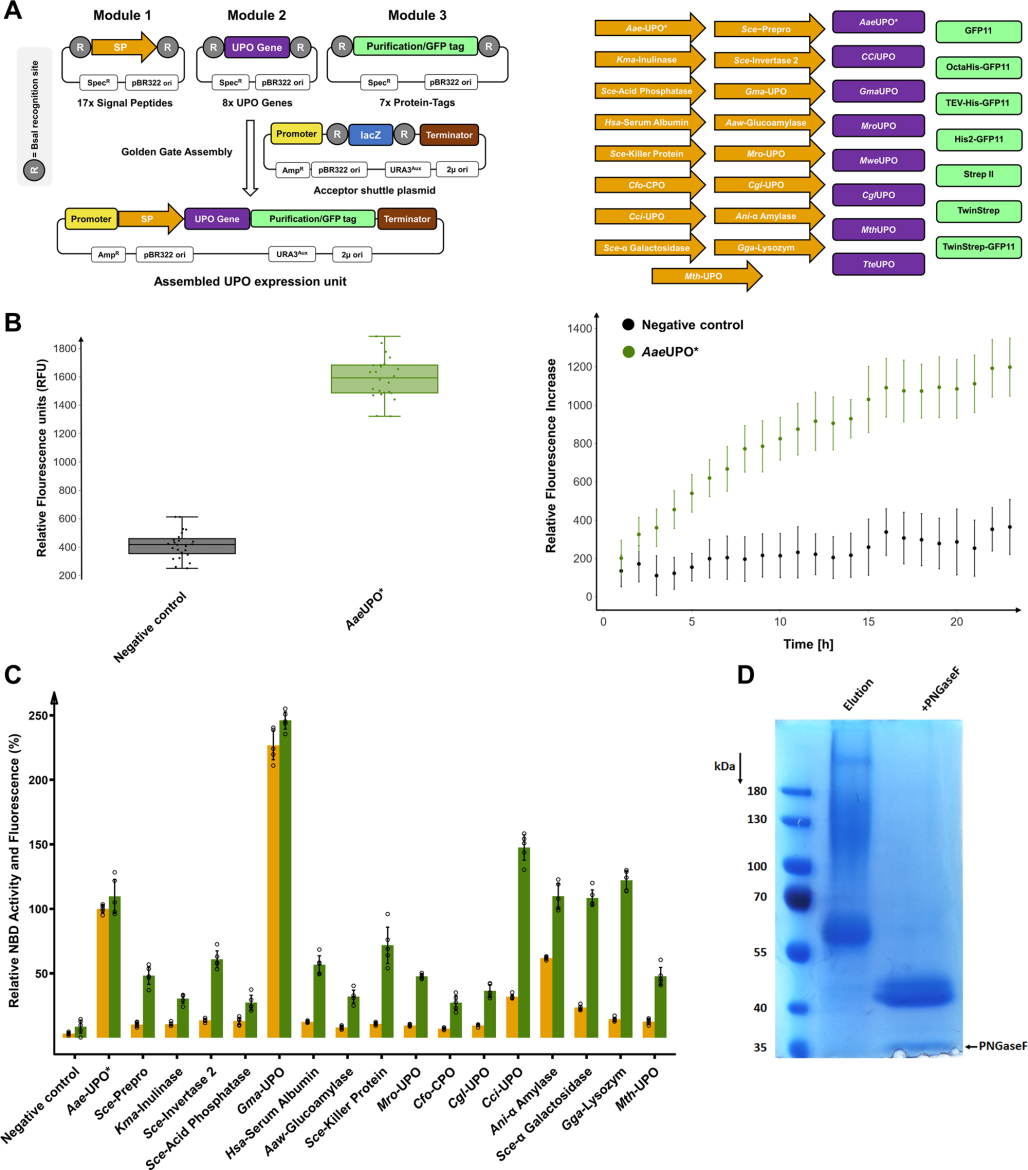
The Golden Gate system consisting of the modules signal peptide, UPO gene and protein-tag and its functional verification regarding split-GFP assay, signal peptide shuffling and purification using the model UPO *Aae*UPO* in *S. cerevisiae*. **A** *Left*: Concept of the modular Golden Gate system as a tripartite system, consisting of signal peptide (SP; contains ATG start codon), UPO gene (lacking start and stop codon) and *C*-terminal Tag (contains stop codon). *Right*: Overview of the individual parts of the modular shuffling systems, containing 17 signal peptides, 8 UPO genes and 7 *C*-terminal protein tags. Detailed sequence information of all parts can be found in Supplementary Tables 4 and 5. **B** Quantification of the UPO secretion in *S. cerevisiae* using the split-GFP system. Two constructs were utilised for testing, namely a previously derived yeast secretion variant of *Aae*UPO (*Aae*UPO*) and further including a *C*-terminal GFP11. The acceptor shuttle plasmid (pAGT572_Nemo 2.0) was used as negative control. *Left:* biological replicates (*n* = 24) of *Aae*UPO* and the negative control were screened within the split-GFP assay. Relative fluorescence units (RFU) were measured at 0 and 72 h after adding GFP1-10. Values are shown as boxplots (*Aae*UPO*: median = 1589, s.d. 8.9%; negative control: median = 416, s.d. 22.6%) with individual data points shown as dots. *Right:* Continuous fluorescence measurements (24 h; 23 time points) of each construct. Data are mean of fluorescence − background (background = first measurement after 1 h) ± s.d. of biological replicates (*n* = 24). **C** Screening of the constructed signal peptide shuffling library utilising *Aae*UPO* as reference protein. Values for 5-nitro-1,3-benzodioxole (NBD) conversion (orange bars) and fluorescence by split-GFP assay (green bars) were normalised to the previously used *Aae*UPO SP* -*Aae*UPO* construct (100%). Data are mean ± s.d. of biological replicates (*n* = 5). Primary data are displayed within the Source data file. Detailed information on the origin and the sequence of the signal peptides can be found in Supplementary Table 4. **D** SDS-PAGE analysis of *Aae*UPO* after one step TwinStrep tag^®^ purification, utilising the designed TwinStrep-GFP11 purification/detection combination tag. Additionally, *Aae*UPO* was subjected to enzymatic deglycosylation by PNGaseF and analysed (right lane).

In this study, we envisioned a tripartite Golden Gate-based modular system. This system consists of the modules ‘signal peptide’, ‘UPO gene’ and ‘protein-tag’ (Fig. 1A). The ‘protein-tag’ module combines affinity-based purification as well as the enzyme quantification by split-GFP. This *S. cerevisiae* expression system gave rise to a rapid workflow starting from UPO genes to heterologously produced and purified UPOs within 2 to 4 weeks (Supplementary Fig. 2).

To unlock access to higher protein amounts, we designed two fully compatible episomal and one integrative plasmid for UPO production in the methylotrophic yeast *Pichia pastoris*. In total, four active UPOs were heterologously produced in yeast for the first time. The high recombinant UPO yields using *P. pastoris* enabled the enantioselective hydroxylation of a phenethylamine derivative on a preparative scale.

## Results

### The modular Golden Gate UPO expression system

Three modules were designed for pre-defined assembly into an episomal *S. cerevisiae* shuttle expression plasmid. We created 32 modules (Fig. 1A) consisting of 17 signal peptides (Module 1), 8 UPO genes (Module 2) and 7 protein-tags (Module 3) derived from a broad phylogenetic background as summarised in Table 1. Module 3 is employed for affinity-based enzyme purification and/or split-GFP-based protein quantification. To verify the envisioned system for protein quantification, the *C*-terminal GFP11 detection tag (Module 3) was assembled with the previously evolved UPO signal peptide *Aae-*UPO* (Module 1) and the engineered peroxygenase *Aae*UPO* (Module 2)^10,36^. Target gene expression is controlled by a GAL 1.3 promoter, which is repressed in the presence of glucose and strongly induced by galactose, being a truncated version of the widely used GAL1 promoter. The successful split-GFP assay was validated by a significant fluorescence response in the sample with the secreted protein (Fig. 1B).

**Table 1:** Origin of utilised UPO genes and signal peptides for target protein secretion.

Descriptor	Type	Organism	Descriptor	Type	Organism
*Aae*UPO*	Secretion engineered UPO	*Agrocybe aegerita*	*Cfo-*CPO	Chloroperoxidase signal peptide	*Caldariomyces fumago*
*Gma*UPO	Wild-type UPO	*Galerina marginata*	*Cci*-UPO	UPO signal peptide	*Coprinopsis cinerea*
*Cci*UPO	Wild-type UPO	*Coprinopsis cinerea*	*Sce-*α Galactosidase	α Galactosidase signal peptide	*Saccharomyces cerevisiae*
*Mro*UPO	Wild-type UPO	*Marasmius rotula*	*Sce-*Prepro	α factor signal peptide	*Saccharomyces cerevisiae*
*Mwe*UPO	Wild-type UPO	*Marasmius wettsteinii*	*Sce*-Invertase 2	Invertase 2 signal peptide	*Saccharomyces cerevisiae*
*Cgl*UPO	Wild-type UPO	*Chaetomium globosum*	*Gma*-UPO	UPO signal peptide	*Galerina marginata*
*Mth*UPO	Wild-type UPO	*Myceliophthora thermophila*	*Aaw*-Glucoamylase	Glucoamylase signal peptide	*Aspergillus awamori*
*Tte*UPO	Wild-type UPO	*Thielavia terrestris*	*Mro*-UPO	UPO signal peptide	*Marasmius rotula*
*Aae-*UPO*	Engineered signal peptide of *Aae*UPO	*Agrocybe aegerita*	*Cgl-UPO*	UPO signal peptide	*Chaetomium globosum*
*Kma*-Inulinase	Inulinase signal peptide	*Kluyveromyces marxianus*	*Ani*-α Amylase	α Amylase signal peptide	*Aspergillus niger*
*Sce*-Acid Phosphatase	Acid phosphatase signal peptide	*Saccharomyces cerevisiae*	*Gga*-Lysozym	Lysozyme signal peptide	*Gallus gallus*
*Hsa*-Serum Albumin	Serum albumin signal peptide	*Homo sapiens*	*Mth*-UPO	UPO signal peptide	*Myceliophthora thermophila*
*Sce*-Killer Protein	Killer protein signal peptide	*Saccharomyces cerevisiae*			

Module 1, exhibiting 17 distinct signal peptides (SP), is the pivotal part for guiding protein secretion. The diverse signal peptide library consists of sequences originating from *S. cerevisiae*, further yeast organisms, basidiomycetes, ascomycetes and animals (Supplementary Table 4). Seven signal peptide sequences originate from (putative) UPOs and a closely related chloroperoxidase (*Cfu*CPO). To demonstrate the importance of the signal peptide, we assembled the *Aae*UPO* gene (Module 2) and the GFP11 tag (Module 3) with each of the 17 signal peptides (Module 1). UPO secretion levels were monitored by enzymatic activity using the 5-nitro-1,3-benzodioxole (NBD)^37^ assay as well as split-GFP detection (Fig. 1C).

All constructs showed significant secretion levels and enzymatic activities. The signal peptides *Cci*-UPO, *Ani*-α Amylase, *Sce*-α Galactosidase and *Gga*-Lysozyme led to similar protein concentrations as the evolved signal peptide *Aae-*UPO*. The signal peptide *Gma*-UPO resulted in a more than doubled activity and secretion of the *Aae*UPO* enzyme relative to the evolved *Aae*-UPO* signal peptide (220% increase). This observation is particularly impressive considering that the signal peptide *Aae*-UPO* was evolved for the optimised secretion of *Aae*UPO* in *S. cerevisiae* by subjecting it to several rounds of directed evolution^10^. The signal peptide *Gma*-UPO originates from the putative *Galerina marginata* UPO (*Gma*UPO). When correlating normalised enzymatic activity and split-GFP-based fluorescence values of the signal peptide library, in most cases, higher fluorescence levels than activity values were measured. This observation indicates the occurrence of differing *Aae*UPO* enzyme variants depending on respective signal peptide cleavage. This could be due to the great diversity of the utilised signal peptides likely resulting in differing *N*-termini and affecting the enzymatic activity of the processed enzyme.

To give rise to a general, one-step protein purification protocol for UPOs, Module 3 was further extended to allow for simultaneous affinity-based protein purification and GFP11-based fluorescence detection. Several versions of the GFP11 tag in combination with Strep^®^- or Hexa/Octahistidine-affinity tags were generated and tested (Supplementary Table 5)^38,39^. We used the protein tags with the previously identified beneficial combination of signal peptide (*Gma*-UPO, Module 1) and UPO (*Aae*UPO*, Module 2) and identified the TwinStrep-GFP11 protein tag as most suitable. This tag consists of a double 8 amino acid Strep II tag (Twin-Strep^®^-tag)^40^ and a *C*-terminal GFP11 sequence. Comparison of the modules GFP11 and TwinStrep-GFP11 revealed unaltered enzymatic activities but a significantly higher fluorescence response for the TwinStrep-GFP11 construct (Supplementary Fig. 3). This difference is probably due to better accessibility of the terminal GFP11 portion since the overall size of the tag is increased (27 vs. 59 amino acids), and several flexible linkers are included. SDS PAGE analysis revealed the successful one-step purification of the mature protein *Aae*UPO* (Fig. 1D). The positioning of an N-terminal Strep II protein-tag revealed greatly diminished UPO activity (Supplementary Fig. 4) and was therefore not further investigated.

### Utilisation of the modular system for the heterologous production of novel UPOs

To demonstrate that the modular system can provide quick access to produced UPOs, we choose seven UPO genes to be expressed in *S. cerevisiae* while three being undescribed putative UPOs. Four UPOs were previously described and produced in their natural hosts—*Marasmius rotula* UPO (*Mro*UPO)^41^, *Marasmius wettsteinii* UPO (*Mwe*UPO)^8^, *Chaetomium globosum* UPO (*Cgl*UPO)^9^—or heterologously expressed in an *Aspergillus oryzae* strain (*Coprinopsis cinerea* UPO (*Cci*UPO))^42^.

Two putative UPO sequences were selected based on sequence alignments and data bank searches using the short-type peroxygenase *Cgl*UPO as a template. Two sequences were retrieved, originating from fungi classified as thermophilic: *Myceliophthora thermophila* (*Mth*UPO) and *Thielavia terrestris* (*Tte*UPO)^43^, bearing 72% and 51% sequence identity to *Cgl*UPO, respectively (Supplementary Table 10). The predicted long-type UPO gene *Gma*UPO is derived from the basidiomycete *Galerina marginata* and was selected based on its high sequence identity (71 %) with *Aae*UPO*.

All genes were introduced as modules (Module 2) into the Golden Gate system and subjected to random shuffling utilising all 17 signal peptides (Module 1).

**Fig. 2:**
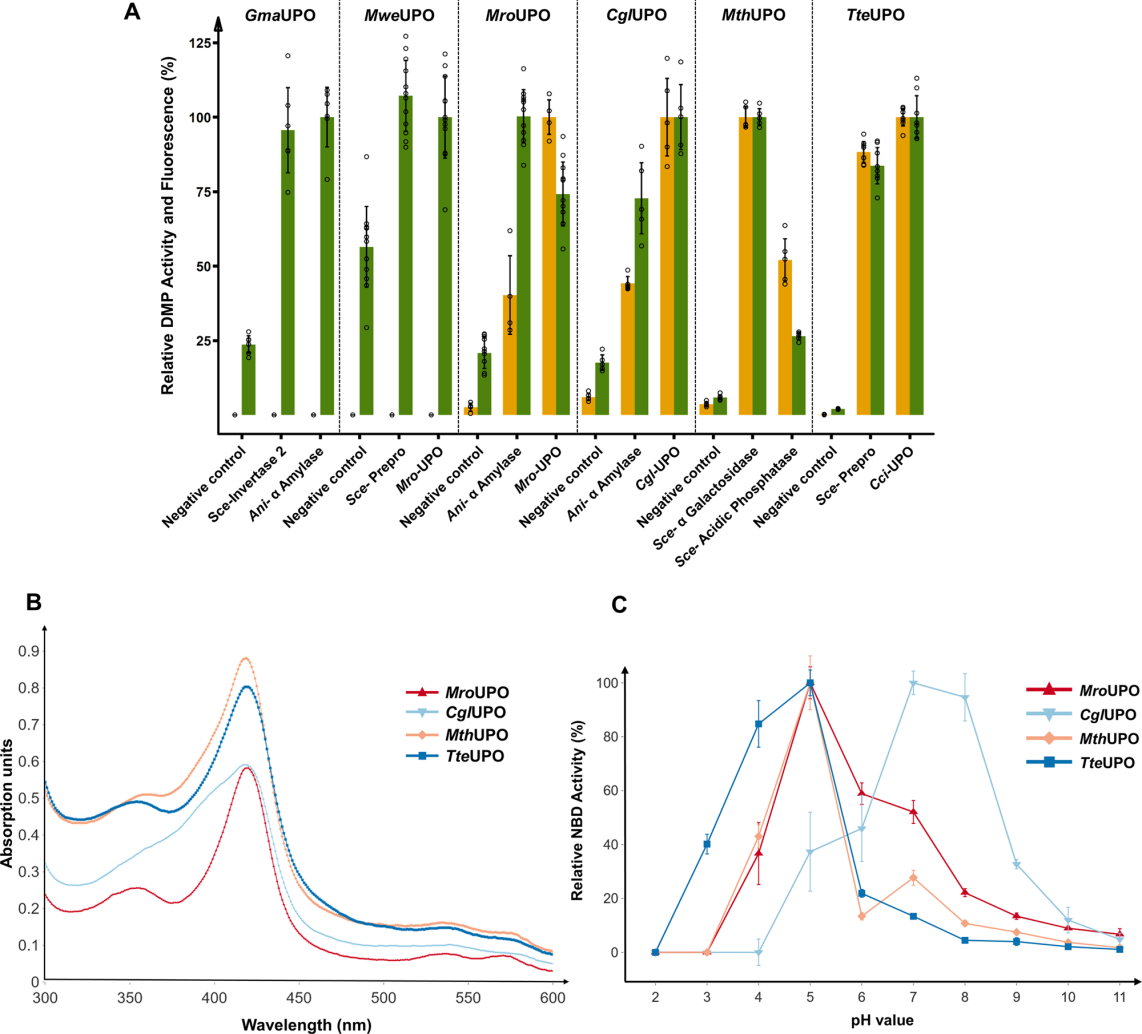
Through signal peptide shuffling identified novel UPO construct and their analysis of UV absorption spectra and pH profiles. **A** Golden Gate signal peptide shuffling was applied for the testing of described and putative UPO genes, and the two best signal peptide/UPO gene combinations are displayed. *Gma*UPO, *Mwe*UPO, *Mro*UPO and *Cgl*UPO were screened in combination with a GFP11-tag. *Mth*UPO and *Tte*UPO were screened using the TwinStrep-GFP11 protein tag. UPO enzyme activity was determined by monitoring the conversion of 2,6-dimethoxyphenol (DMP) to coerulignone. The highest average fluorescence (split-GFP) and conversion values (DMP) within one enzyme panel were set to 100%, and the other values normalised accordingly. Data are mean ± s.d. of biological replicates (*n* ≥ 4). Corresponding primary data are displayed within the Source data file. **B** UV-Vis absorption spectra of the purified peroxygenases *Mro*UPO, *Cgl*UPO, *Mth*UPO and *Tte*UPO in the wavelength range between 300 and 600 nm (measurement interval: 1 nm). **C** pH profiles of *Mro*UPO, *Cgl*UPO, *Mth*UPO and *Tte*UPO catalysed enzymatic conversion of 5-nitro-1,3-benzodioxole (NBD) to 4-nitrocatechol. The highest mean activity of a respective enzyme was set to 100% and the other values normalised accordingly. Data are means ± s.d. of measurements performed in triplicates. Corresponding primary data are displayed within the Source data file.

Out of the seven UPO genes, six could be secreted by *S. cerevisiae* in combination with at least two signal peptides (Fig. 2A). *Cci*UPO exhibited no secretion with any of the signal peptides. *Mwe*UPO and *Gma*UPO were identified through the split-GFP assay, but no activity was detected using the colorimetric 2,6-dimethoxyphenol (DMP) assay^12^. *Mwe*UPO, *Mro*UPO and *Cgl*UPO were the only UPOs, which showed the highest activities with their endogenous signal peptides, *Mro*UPO and *Mwe*UPO sharing the identical native signal peptide. *Mth*UPO and *Tte*UPO showed remarkable secretion levels within the microtiter plate setup, leading to 17-fold (*Mth*UPO) and 50-fold (*Tte*UPO) split-GFP signal intensities above background level. A high signal peptide promiscuity was observed for *Mth*UPO and *Tte*UPO with at least 5 and 8 suitable signal peptides, respectively (Supplementary Figs. 5 and 6).

### Purification and characterisation of the identified UPOs

All successfully secreted UPOs in combination with their best signal peptides were equipped with the TwinStrep-GFP11 tag, produced in 1 L shake flask scale, and purified by affinity chromatography. The occurrence and primary sequence of each UPO was confirmed by tryptic digest and mass spectrometric peptide analysis (Supplementary Tables 6 and 8). *Aae*UPO* analysis revealed the amino acids ‘EPGLPP’ being the first detectable residues at the *N*-terminus in accordance with previous results^15^. This finding indicates that the employed signal peptide *Gma*-UPO leads to a comparable cleavage pattern as the evolved *Aae*-UPO* signal peptide. The split-GFP response and the NBD activity also exhibited the same ratio for both signal peptides (Fig. 1C), which further strengthens the point of a similar cleavage pattern. *Mro*UPO and *Mwe*UPO were produced utilising their native signal peptide (*Mro*-UPO SP). Fragments derived from the signal peptide *Mro-*UPO (11 amino acids for *Mro*UPO and 9 amino acids for *Mwe*UPO) were identified by MS analysis, suggesting a different cleavage pattern compared to the natural host^8^. Obtained *N*-termini of *Gma*UPO and *Mth*UPO are in good agreement with the predicted cleavage sites based on alignments with the enzymes *Aae*UPO* and *Cgl*UPO, respectively. The *N*-terminus of *Cgl*UPO could not be resolved. For *Tte*UPO, a peptide fragment of 10 amino acids of the utilised signal peptide (*Sce*-Prepro) was identified.

*Gma*UPO and *Mwe*UPO were not further studied as the purified enzymes did not exhibit any activity towards the colorimetric peroxygenase substrates DMP and NBD. For these enzymes, we were not able to obtain pure elution samples for subsequent measurements of native as well as CO differential absorption spectra.

Biochemical parameters were therefore determined for *Mro*UPO, *Cgl*UPO, *Mth*UPO and *Tte*UPO. UV absorption profiles showed the expected characteristic peroxygenase haem-thiolate features. A Soret band with a maximum around 420 nm (*Mro*UPO: 419 nm; *Cgl*UPO: 418 nm; *Mth*UPO: 420 nm and *Tte*UPO: 419 nm) and two Q-bands in the range of 537 to 546 and 569 to 573 nm (Fig. 2B)^2^ were detected. *Cgl*UPO revealed a broader Soret band shape as well as less pronounced Q-bands. The respective carbon monoxide complexes exhibited absorption maxima around 444 nm (Supplementary Fig. 7).

Protein purity and glycosylation were analysed by SDS-PAGE. Native deglycosylation was performed using PNGaseF (Supplementary Fig. 8). All obtained molecular weights after deglycosylation were in approximate agreement with the calculated weight based on the primary sequence and peptide analysis by mass spectrometry. *Mro*UPO exhibited a defined band at approx. 42 kDa that was slightly shifted towards lower molecular weight after deglycosylation. *Cgl*UPO revealed a smeared band in the range of 55–130 kDa. Deglycosylation led to the occurrence of two distinct protein bands of approx. 37 and 33 kDa indicating different protein subtypes. *Mth*UPO and *Tte*UPO showed an intensive smeared band in the range of 55–200 kDa. This smear was converted into distinct protein bands upon deglycosylation with approx. 38 kDa and 36 kDa for *Mth*UPO and *Tte*UPO, respectively.

To gain insights into the impact of the glycosylation on the activity of the respective enzyme, UPOs were deglycosylated in the native state and assessed for their activity towards NBD (Supplementary Fig. 9). The enzymatic activity of *Mro*UPO was in comparison least affected with a decrease of approx. 80%, whereas in the case of *Cgl*UPO no activity could be obtained after deglycosylation. The activity was substantially impaired as well for *Tte*UPO and *Mth*UPO, leading to a complete loss and approx. 85% decrease, respectively, in enzymatic activity.

We next evaluated the pH-dependencies of the enzymes using NBD as a substrate (Fig. 2C). *Mro*UPO, *Mth*UPO and *Tte*UPO exhibited a similar profile with maximum activity at slightly acidic conditions (pH 5), whereas *Cgl*UPO’s activity optimum was detected at pH 7. *Tte*UPO showed a broader tolerance towards lower pH values, retaining medium (pH 3; 40%) and high activity (pH 4.0; 80%) at acidic conditions. The obtained values for *Mro*UPO and *Cgl*UPO are in good agreement with previous data obtained with homologously produced enzyme^9,41^.

### Enzymatic epoxidation and hydroxylation experiments

**Fig. 3:**
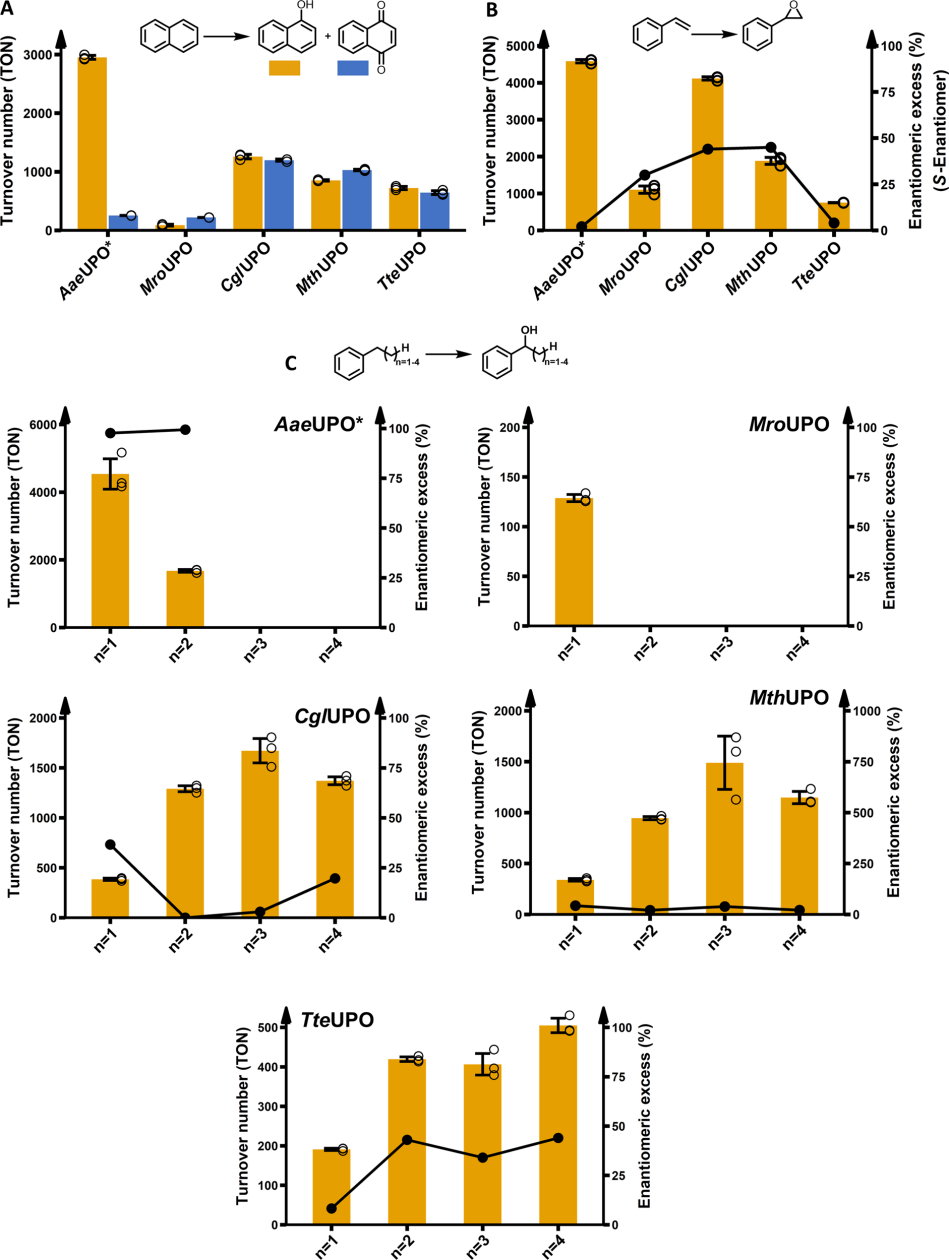
Enzymatic activity assessment of the peroxygenases regarding aromatic hydroxylation, epoxidation and sp^3^-carbon hydroxylation. All reactions were performed for 1 h using 1 mM of substrate. Bar charts display the obtained turnover numbers (TON) within one hour. The lines correspond to the enantiomeric excess %. Data are mean ± s.d. measurements derived from biological triplicates with individual data points shown as circles. See supplementary information for further details. **A** Conversion of naphthalene to naphthol and 1,4-naphthoquinone. **B** Conversion of styrene to styrene oxide. **C** A homologous row of phenylethane, phenylpropane, phenylbutane and phenylpentane hydroxylation, respectively, focusing on hydroxylation of the benzylic carbon. The alcohol enantiomer is indicated by an (R) or (S). The exact enantiomer for phenylpentane was not determined. See Supplementary Fig. 17 for occurrence of side-products. For *Mro*UPO catalysed conversion of phenylethane no enantioselectivity could be determined.

The heterologously produced UPOs were tested towards their substrate specificity and activities by investigating three distinct reaction types: aromatic hydroxylation (sp^2^-carbon), alkene epoxidation and the benzylic hydroxylation (sp^3^-carbon) of phenylalkanes with varying alkyl chain lengths from two to five carbons (Fig. 3). All reactions were performed under the same conditions and assessed for the achieved turnover number (TON) within one hour. Substantially differing behaviour could be observed between *Aae*UPO* and the novel heterologously produced UPOs regarding substrate conversion, specific product formation and stereoselectivity. *Aae*UPO* proved to be the only enzyme displaying a high specificity for single hydroxylation of naphthalene leading to 1-naphthol (92% of the formed product, Fig. 3A). The other UPOs exhibited a strong tendency for further oxidation leading to the dione product 1,4-naphthoquinone. The epoxidation of styrene (Fig. 3B) was efficiently catalysed by *Aae*UPO* (4580 TON) in combination with a poor stereoselectivity (2% *ee*). *Cgl*UPO exhibited comparable epoxidation activities (4110 TON) and an enantioselectivity of 44% *ee*. For *Mth*UPO, TON decreased to 1100 but revealed the highest stereoselectivity (45% *ee*). The studies of the benzylic hydroxylation of phenylalkanes—ranging from phenylethane to phenylpentane—confirmed the known preference of *Aae*UPO* towards short alkane chain length (Fig. 3C)^3^. Starting from 4500 turnovers for the conversion of phenylethane and deteriorating to no product formation and only traces of benzylic hydroxylation using phenylbutane and phenylpentane, respectively (for other product formations see Supplementary Fig. 17). *Cgl*UPO and *Mth*UPO exhibited an inverted trend with increasing product formations for longer alkyl chain lengths, exhibiting the lowest activity for the phenylethane hydroxylation.

The highest activity was detected in both cases using phenylbutane (*Cgl*UPO: 1670 TON, *Mth*UPO: 1490 TON) with only slightly decreased activity for phenylpentane as a substrate and the only significant side-product being the further oxidation of the benzylic alcohol to the corresponding ketone (Supplementary Fig. 17). *Tte*UPO showed a similar preference towards long-chain phenylalkanes with the highest TON for phenylpentane conversion (500 TON). *Tte*UPO represented the only UPO with a significant specificity towards the formation of the *S* alcohol enantiomer for phenylpropane and phenylbutane. For phenylpentane, it revealed the formation of the opposite alcohol enantiomer than the other tested UPOs as well.

### Expanding the modular UPO secretion system to *Pichia pastoris*

**Fig. 4:**
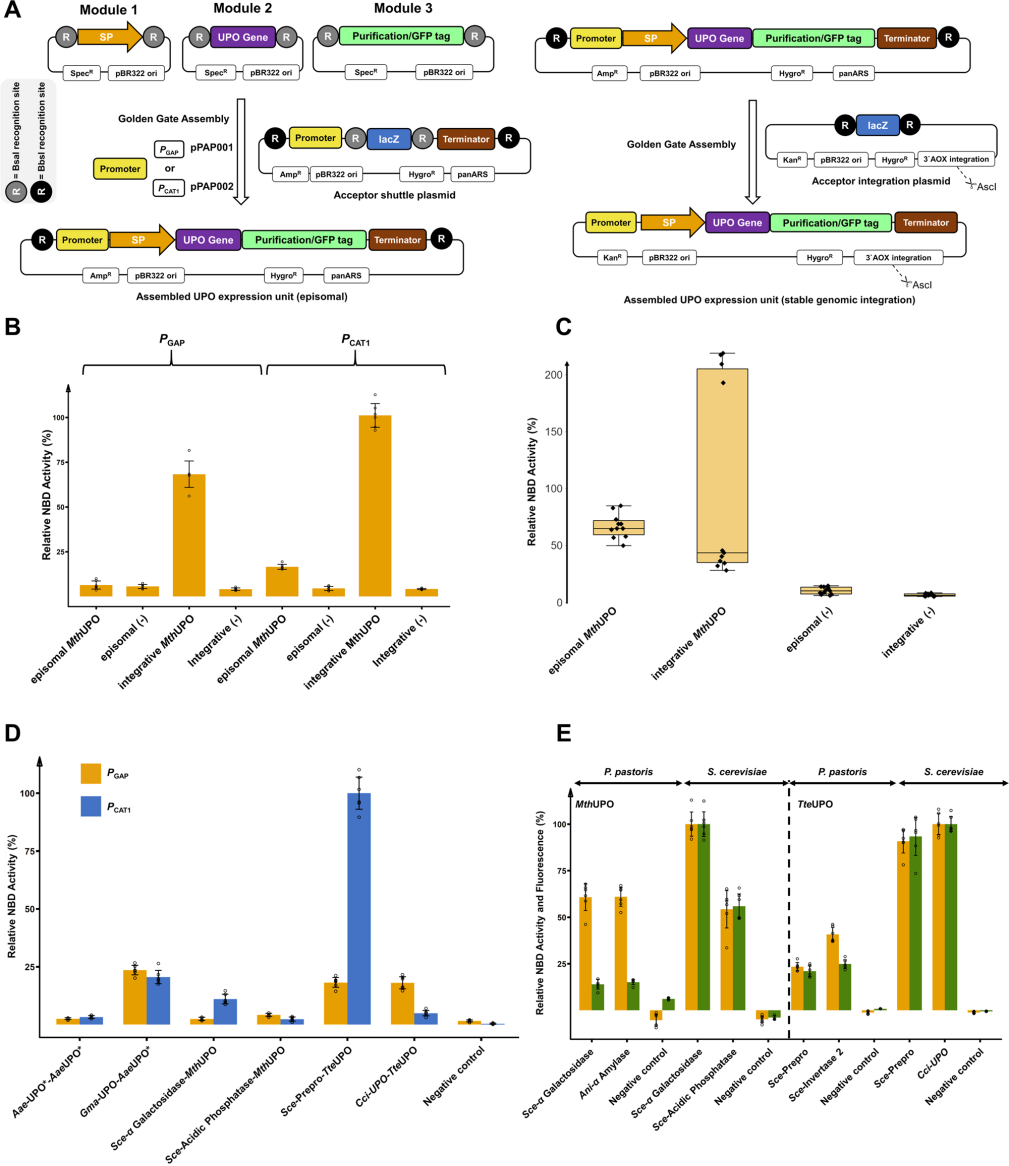
The compatible modular Golden Gate setup utilising episomal and integrative *P. pastoris* plasmids and its application. **A** *Left*: Overview of the designed episomal *P. pastoris* screening setup. All previously created basic modules are compatible to be used within this system. Two episomal plasmids were designed harbouring the constitutive strong promoter *P*_GAP_ or the strong inducible promoter *P*_CAT1_. *Right:* Identified gene constructs can be directly transferred in a one-pot Golden Gate reaction (+BbsI) from the episomal plasmid to an integrative plasmid. After linearisation (AscI digest) this plasmid can be integrated into the genomic 3’AOX region of *P. pastoris*. **B** Comparison of relative activities of 5-nitro-1,3-benzodioxole (NBD) conversion for different *P. pastoris* constructs bearing the tripartite combination of α Galactosidase signal peptide-*Mth*UPO-TwinStrep-GFP11. *P*_GAP_ bearing constructs were screened utilising Glucose (1.5 % (w/v)) as sole carbon source. *P*_CAT1_ bearing constructs were screened utilising a dual feeding strategy (0.5% (v/v) glycerol and 1.5% (v/v) methanol) as primary and inducible carbon sources. The highest expression mean is set to 100% and all data normalised. Data are mean ± s.d. of biological replicates (*n* = 6) originating from streak outs of one previously screened colony of the respective construct. **C** Comparison of relative activities of NBD conversion of *P*_CAT1_-based constructs bearing the tripartite combination of α Galactosidase signal peptide-*Mth*UPO-TwinStrep-GFP11. Box plots of biological replicates (*n* = 11) of individual *P. pastoris* colonies for each construct. The highest expression mean is set to 100% and all data normalised (episomal *Mth*UPO: median = 65, s.d. 15.0%; integrative *Mth*UPO: median = 44, s.d. 83.3%; episomal (-): median = 10, s.d. 28.2%; integrative (-): median = 6, s.d. 16.4%). **D** Comparison of relative activities of NBD conversion for different episomal *P. pastoris* constructs (6 biological replicates each) using the indicated signal peptide-UPO combinations as well as a TwinStrep-GFP11 tag. *P*_GAP_ (yellow bars) and *P*_CAT1_ (blue bars). The highest expression is set to 100%, and all data are normalised accordingly. Data are mean ± of biological replicates (*n* = 6). **E** Direct comparison of episomal UPO production of the two best signal peptide-UPO combinations for *Mth*UPO and *Tte*UPO as identified by a previously performed signal peptide shuffling approach in both yeast species. Episomal *P. pastoris* expressions utilising *P*_CAT1_. The highest mean expression and activity for each enzyme is set to 100%, and all data are normalised. Data are mean ± s.d. of biological replicates (*n* = 6). NBD conversion activity (orange) and relative fluorescence units (green). All primary data are displayed within the Source data file.

The methylotrophic yeast *P. pastoris* (syn. *Komagataella phaffii*) constitutes an attractive heterologous production host with a steadily growing toolbox of valuable synthetic biology parts such as plasmids, promoters and signal peptides^44,45^. *P. pastoris* can reach high cell densities, efficiently perform post-translational modifications such as glycosylation and disulfide-linkage and offers a set of strong and tightly regulated promoters for target gene expression. Amongst other factors, these properties render *P. pastoris* a widely used eukaryotic host for the large-scale industrial production of therapeutic proteins and enzymes^46^. We investigated the adaptation of the modular system for use in *P. pastoris*. Therefore, two novel episomal *P. pastoris* expression plasmids were designed and assembled. They contain a previously described autonomously replicating sequence coined panARS, which confers episomal stability and a hygromycin B marker gene for antibiotic selection^47–49^. The constructed episomal plasmids differ by the employed promoter: the strong constitutive glyceraldehyde-3-phosphate dehydrogenase promoter (*P*_GAP_, plasmid pPAP001) and the recently described strong methanol inducible catalase 1 promoter (*P*_CAT1_, pPAP002)^50^. The plasmids were constructed to allow direct implementation of the tripartite modular UPO secretion system, consisting of Module 1 (signal peptide), Module 2 (UPO gene) and Module 3 (*C*-terminal tag, Fig. 4A; left). To further allow the genomic integration to generate stable *P. pastoris* cell lines for antibiotic-free large-scale enzyme production in shake flasks or fermenters, a third plasmid (pPAP003) was constructed. The episomal plasmids are designed to enable direct transfer of the identified best transcription unit (promoter-signal peptide-gene-tag-terminator) combination to the integration plasmid. This transfer requires only an additional Golden Gate assembly reaction using the restriction enzyme BbsI (Fig. 4A; right).

We tested all *P. pastoris* plasmids using the newly discovered peroxygenase *Mth*UPO in combination with the *Sce*-α Galactosidase signal peptide. The constructs proved to be functional and led to an NBD conversion signal (Fig. 4B). *P*_GAP_-based secretion was generally lower in comparison to *P*_CAT1_, and the episomal *P*_GAP_ *Mth*UPO activity was not distinguishable from the negative control. The integrative plasmids outperformed their episomal counterparts significantly with a factor of approx. 5 for *P*_CAT1_. A similar observation however in a varying degree was made testing the enzymes *Aae*UPO* and *Tte*UPO (Supplementary Fig. 10), indicating that the integrative constructs are in general promoting higher UPO secretion levels than their episomal counterparts.

To gain insights into interclonal variabilities of UPO secretion, episomal and integrative plasmids were transformed into *P. pastoris*. Individual colonies were cultivated and tested for UPO secretion. The episomal construct showed diminished mean activity but a substantially lower clonal variability than the integrative plasmid when tested with NBD (Fig. 4C). This high variability of the secretion level for the integrative plasmid is presumably due to divergent numbers of copy insertions into the *P. pastoris* genome, which is a commonly occurring feature in this organism^51–53^, and might also lead based on the Hygromycin B selection marker to different colony sizes (Supplementary Fig. 11).

To investigate and compare the secretion levels of episomal *P*_GAP_ and *P*_CAT1_ harbouring plasmids, twelve constructs were generated harbouring the peroxygenases *Aae*UPO*, *Mth*UPO and *Tte*UPO. All promoter combinations (*P*_GAP_ and *P*_CAT1_) and the two previously identified signal peptides were constructed in combination with the respective UPO gene and analysed for NBD activity. All constructs resulted in a significant NBD conversion (Fig. 4D). The previously observed 220% improved *Aae*UPO* secretion in *S. cerevisiae* by combining *Aae*UPO* with the signal peptide *Gma-*UPO was found to be even more pronounced using the episomal *P. pastoris* constructs (*P*_CAT1_: 620%). Besides the striking influence of the promoter on the secretion level, also the combination of the signal peptide and the promoter proved to be pivotal. For *Tte*UPO, using the promoter *P*_CAT1_ in combination with the *Sce*-Prepro signal peptide led to the highest detected activity with a 20-fold higher signal compared to the *Cci*-UPO signal peptide. The same signal peptide variations employing the *P*_GAP_ promoter, however, resulted in similar secretion levels. This demonstrates besides the crucial role of the chosen signal peptide (Figs. 1C and 2A) an additionally pivotal synergistic influence of the promoter/signal peptide combination on the UPO secretion.

To gain insights into the different signal peptide preferences for secretion in *P. pastoris*, the signal peptide shuffling approach was repeated in *P. pastoris* using *Mth*UPO and *Tte*UPO and choosing the episomal *P*_CAT1_ bearing plasmid (Supplementary Figs. 12 and 13). For *Mth*UPO the signal peptides *Sce*-α Galactosidase and *Ani*-α Amylase proved to be most suitable, and *Sce*-Prepro and *Sce*-Invertase 2 were identified as top hits for *Tte*UPO. Interestingly, *Sce*-Invertase 2 has not been identified amongst the top hits in *S. cerevisiae* whereas the best signal peptide (*Cci*-UPO) for secretion in *S. cerevisiae* (Fig. 4D) was not identified in the *P. pastoris* screen.

To compare episomal *S. cerevisiae* and *P. pastoris* secretion, the two best performing constructs for *Mth*UPO and *Tte*UPO were selected. This species comparison (Fig. 4E) indicates that the episomal *S. cerevisiae* secretion is superior to the episomal *P. pastoris* production. In the case of *Mth*UPO, both *P. pastoris* constructs led to approx. 60% of NBD conversion in comparison to the most suitable *S. cerevisiae* construct, while already exhibiting higher NBD conversion rates than the second most suitable signal peptide for secretion in *S. cerevisiae* (*Sce*-Acidic Phosphatase). The split-GFP fluorescence assay revealed a diminished response for the *P. pastoris* setup relative to the *S. cerevisiae* constructs. Regarding *Tte*UPO, the best *P. pastoris* construct (*Sce*-Invertase 2) led to approx. 40% of relative NBD conversion when compared to the best *S. cerevisiae* construct (*Cci*-UPO). For *Tte*UPO the split GFP assay followed a linear pattern when comparing species, without revealing a diminished response for *P. pastoris*.

### Comparison of shake flask UPO production in *P. pastoris* and *S. cerevisiae*

**Fig. 5:**
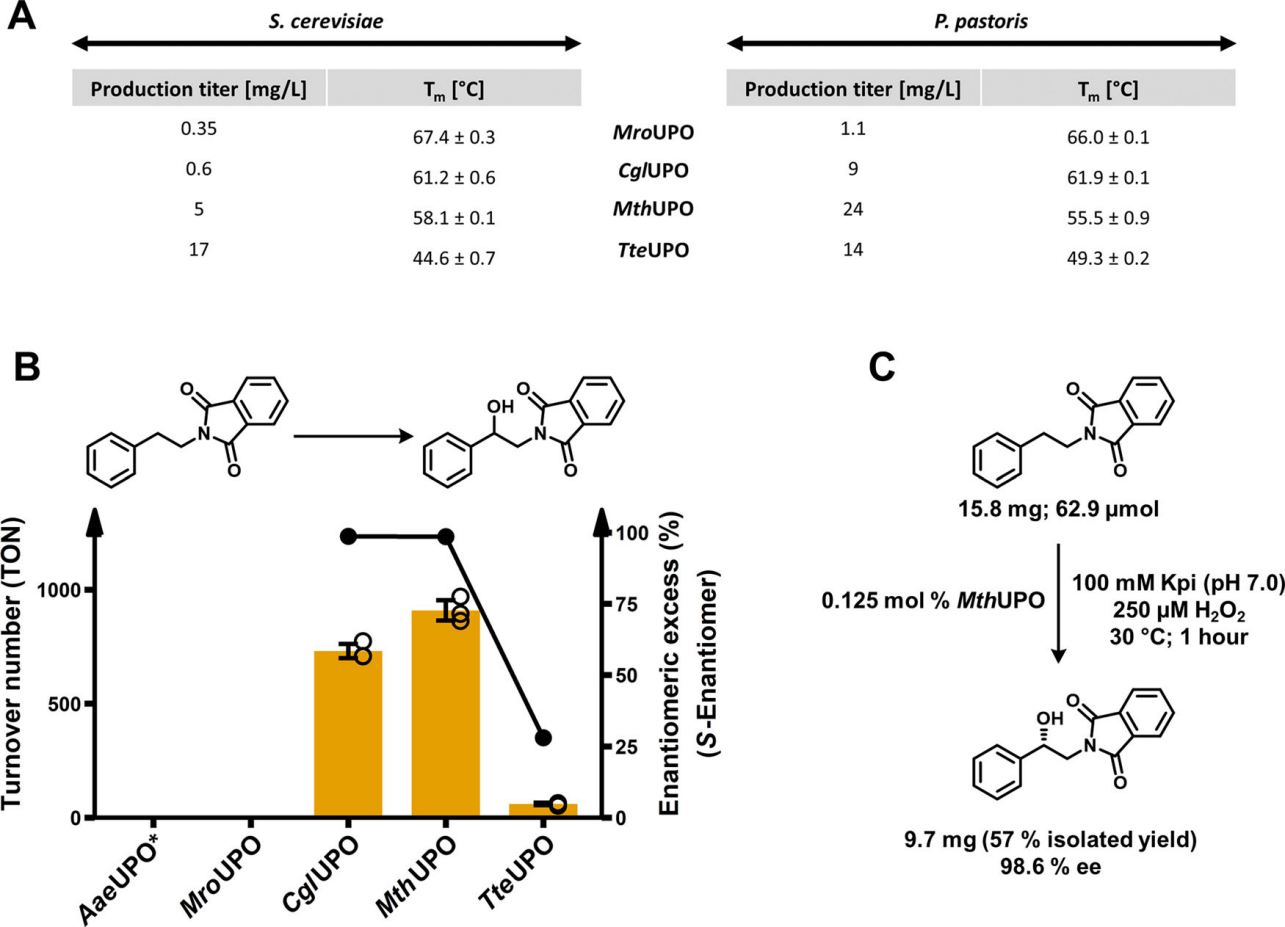
Expression yields and thermostabilities of UPOs derived from the different yeast systems and conversion of a phenethylamine derivative on analytical and preparative scale. **A** Comparison of volumetric production titre of recombinant UPOs in shake flask scale (1 L) between *S. cerevisiae* (episomal construct) and *P. pastoris* (integrative construct) as obtained after ultrafiltration of the respective culture supernatant. UPOs were produced and secreted utilising their natural signal peptide (*Mro*UPO and *Cgl*UPO) or a previously identified suitable exogenous signal peptide *Mth*UPO (*Sce*- α Galactosidase) and TteUPO (*S. cerevisiae*: *Cci*-UPO; *P. pastoris*: *Sce*-Prepro). For all *P. pastoris* production setups the methanol inducible promoter *P*_CAT1_ was utilised. Thermal denaturation midpoints (*T*_m_) for the four UPOs produced in both organisms were determined in biological triplicates using purified protein samples (in 100 mM potassium phosphate; pH 7.0) using differential scanning fluorimetry (DSF). **B** Bar chart showing turnover number within one hour for the benzylic hydroxylation of *N*-phthaloyl-phenethylamine by *P. pastoris* produced *Aae*UPO*, *Mro*UPO, *Cgl*UPO, *Mth*UPO and *Tte*UPO. Turnover data are mean ± s.d. of measurements made in triplicates. TON determined by GC-MS and *ee*% by chiral HPLC (Supplementary Figs. 18–20). **C** Preparative scale conversion of *N*-phthalimide protected phenethylamine using *P. pastoris* produced *Mth*UPO.

By using the constructed integrative plasmid pPAP003 and the *P*_CAT1_ promoter, stable *P. pastoris* cell lines were constructed to produce five UPOs: *Aae*UPO*, *Mro*UPO, *Cgl*UPO, *Mth*UPO and *Tte*UPO (Fig. 5A). Utilising *P. pastoris* as host led to substantially higher production titres in all cases, except for *Tte*UPO. The rather low yields of *Mro*UPO and *Cgl*UPO produced in *S. cerevisiae* could be increased substantially when using *P. pastoris* (*Mro*UPO: 3-fold, *Cgl*UPO: 15-fold). The *Mth*UPO production yield was improved 5-fold. Regarding *Tte*UPO, the product titre was decreased in *P. pastoris* by approx. 20%, however, still maintaining an overall high yield. The production titres of *S. cerevisiae* derived *Tte*UPO (17 mg/L), and *P. pastoris* derived *Mth*UPO (24 mg/L) are the highest yields for shake flask cultivation of recombinant fungal peroxygenases reported thus far. The transfer of the expression system to a fed-batch bioreactor might further elevate protein titres due to the higher cell densities achievable. In previous work, this transfer into a bioreactor resulted in 27-fold improved product titre of 217 mg/L for *Aae*UPO*^15^. All proteins were purified using the attached TwinStrep-tag and analysed by SDS-PAGE (Supplementary Fig. 14). Highly pure enzyme preparations were obtained after one-step TwinStrep purification. Based on the successful production in both organisms, thermostability values (denaturation midpoint; *T*_m_) of the four UPOs were assessed using differential scanning fluorimetry (Fig. 5A). The obtained values of the respective UPOs derived from both organisms proved to be alike to a variation of 0.7 to 4.7 °C. The highest thermostability values were obtained for *Mro*UPO with 67.4 and 66.0 °C produced by *S. cerevisiae* and *P. pastoris*, respectively. The two UPOs derived from thermophilic fungi, *Mth*UPO and *Tte*UPO, exhibited no superior thermostability when compared to the closest related enzyme *Cgl*UPO. *Tte*UPO revealed the lowest thermostability in the tested group with 44.6 and 49.3 °C for *S. cerevisiae* and *P. pastoris*, respectively.

### Enantioselective hydroxylation of an *N*-protected phenethylamine on a preparative scale

To gain insights into the ability of the enzymes to convert industrially relevant molecules in an enantioselective manner, we selected *N*-protected phenethylamine as a substrate. The hydroxylation of phenethylamine derivatives at the benzylic position provides access to a plethora of pharmaceutically important classes like beta-blockers and sympathomimetics^54^.

The peroxygenases *Aae*UPO*, *Mro*UPO, *Cgl*UPO, *Mth*UPO and *Tte*UPO were produced in *P. pastoris*, purified, and assessed for their activity on *N*-phthaloyl-phenethylamine. *Aae*UPO* and *Mro*UPO exhibited no formation of the benzylic alcohol product, and *Tte*UPO performed 60 TON within an hour while achieving an enantioselectivity of 28% *ee* (Fig. 5B, Supplementary Figs. 18 and 20). *Cgl*UPO and *Mth*UPO revealed the highest activities with 730 and 908 TON, respectively, within the 1 h reaction setup (Supplementary Fig. 18). *Mth*UPO showed an over-oxidation to the ketone amounting to 222 TON (Supplementary Fig. 19). The enantioselectivity of alcohol formation proved to be excellent for *Cgl*UPO and *Mth*UPO with 98.7% *ee* and 98.6% *ee* (Supplementary Fig. 20).

Harnessing the high production titre of *Mth*UPO in *P. pastoris* (24 mg/L) in combination with the previously observed good substrate conversion and high enantioselectivity we aimed for the proof-of-concept enantioselective synthesis of (*S*)-( + )-2-*N*-Phthaloyl-1-phenylethanol on a preparative scale (Fig. 5C). In a first upscaling reaction (300 mL total volume) 0.125 mol% of *Mth*UPO derived without further purification from concentrated *P. pastoris* supernatant were used as catalyst loading. The upscaled reaction (30 °C; 1 h) led to the synthesis of 9.70 mg (S)-( + )-2-*N*-Phthaloyl-1-phenylethanol (57% purified yield) and an enantiomeric excess of 98.6% (Supplementary Fig. 20).

## Discussion

Fungal unspecific peroxygenases (UPOs) have gained substantial interest as versatile hydroxylation catalyst since their initial discovery 16 years ago^2^. The most significant limitation for the wider application of UPOs arguably remains their heterologous production utilising a fast-growing host. Thus far, only one UPO could be produced and engineered within a system amenable to high throughput: the *S. cerevisiae* secretion variant *Aae*UPO*^10^.

Building on the therein developed expression setup, we started our endeavour to construct a versatile UPO secretion system. The constructed Golden Gate-based platform consists of a signal peptide library (Module 1), UPO genes (Module 2) and protein-tags (Module 3). This format enabled the first report of successful yeast secretion of six UPOs—two of them (*Mth*UPO and *Tte*UPO) derived from genome and secretome data had not been characterised as UPOs before^43^. The whole expression platform could be subsequently transferred to *P. pastoris*, resulting in excellent UPO expression yields allowing for preparative scale hydroxylation reactions.

Since the only enzyme out of the panel that could not be produced (*Cci*UPO) belongs to the group of long-type UPOs, and it previously took considerable effort to engineer the long-type UPO *Aae*UPO towards secretion in yeast, one could argue that the heterologous production of long-type UPOs seems to be more challenging^10^. In fact, *Mro*UPO, *Cgl*UPO, *Mth*UPO and *Tte*UPO, which could be initially produced as non-engineered wild-type enzymes in yeast and characterised within the scope of this work, all belong to the class of short-type UPOs. Recent work in our laboratory suggests that gene shuffling of long-type UPOs can offer a viable option to obtain a library of active and structurally diverse long-type UPOs expanding the panel of available recombinant peroxygenases^55^.

The hypothesised pivotal role of the employed signal peptides for the successful secretion of UPOs was manifested within this study. Yeast secretion signal peptides generally consist of three main parts: (1) an *N*-terminal positively charged domain, (2) a hydrophobic core and (3) a cleavage site region exhibiting mostly uncharged residues^56^. The length and amino acid composition of the signal peptides, however, can vary substantially. This variation is also reflected by the selected, diverse signal peptide pool in the present paper, which ranges from several predicted and reported UPO signal peptides, commonly utilised endogenous *S. cerevisiae* signal peptides up to human (*Hsa*-Serum Albumin) and bird-derived (*Gga*-Lysozym) sequences. This set includes peptides ranging from 17 to 87 amino acids in lengths and exhibits substantial sequence alterations to provide a high diversity. Apart from the UPO-derived signal peptides, this signal peptide pool was selected based on previous reports on recombinant protein secretion in *S. cerevisiae*^56,57^. In case of *Aae*UPO* the native signal peptide was evolved by means of MORPHING^36^ resulting in a 27-fold increased total secretion through addition of the four mutations F12Y/A14V/R15G/A21D^10^.

Exchanging this evolved signal peptide sequence with the corresponding *Gma*-UPO sequence resulted in a further 2.2-fold improved secretion in *S. cerevisiae* and even 6-fold enhancement in *P. pastoris*. The signal peptides *Aae-*UPO* and *Gma-*UPO display a high sequence identity and similarity of 50 and 73%, respectively. *Gma*-UPO exhibits two additional amino acids, and these insertions are the two hydrophobic amino acids alanine and leucine before position 12. These amino acid additions might be pivotal for the increased secretion level. Interestingly, the overall suitability of other signal peptides based on specific enzyme activity proved to be low (Fig. 1C), even though in many cases comparable split-GFP values were obtained when comparing respective signal peptides to the evolved *Aae*-UPO* signal peptide. This low promiscuity towards functional signal peptides points towards the occurrence of differing *N*-termini of the mature enzyme. A hypothesis, that has been indirectly stated before in the context of this evolved enzyme^58^ as introduced mutations within the mature protein (V57A and V75I) are argued to preserve the natural *N*-terminus (EPGLPPPGPL) of the mature protein when produced in yeast in connection with the evolved signal peptide. This is contrary to the wild type *Aae*UPO enzyme, where *N*-terminal proteolysis (EPG↓LPPPGPL) occurs^59^. Using the *Gma*-UPO signal peptide for production, we could verify the natural *N*-terminus occurrence (starting AEPGLPP) by peptide analysis (Supplementary Table 8); therefore the cleavage pattern of this signal peptide in yeast seems to be comparable to the evolved signal peptide used in previous studies^10,13,15,58,60^. The hypothesis of low promiscuity also holds in the episomal production attempts in *P. pastoris*, where all identified active constructs exhibited the signal peptide *Aae*-UPO* or *Gma-*UPO, respectively.

The case of *Aae*-UPO* might suggest that the closest related signal peptide in terms of sequence and length demonstrates the highest secretion rates. Regarding *Mth*UPO this only might be true in terms of sequence length. The wild-type signal peptide consists of 17 amino acids, and the most suitable orthologous SPs have a similar length: *Sce*-α Galactosidase (19 aa), *Ani*-α Amylase (20 aa) and *Sce*-Acid Phosphatase (18 aa). However, the sequence similarity of the most suitable signal peptides *Sce*-α Galactosidase and *Ani*-α Amylase is solely 3 and 29%, respectively. In general, a high promiscuity of *Mth*UPO towards a diverse set of signal peptides was observed, leading to the identification of five (*S. cerevisiae*) and eight (*P. pastoris*) suitable signal peptides (Supplemental Figs. 5 and 12). In case of *Tte*UPO, no wild-type signal peptide was retrieved, and this UPO proved to be highly promiscuous, showing no preference for a signal peptide length (17–87 amino acids) or sequence composition whatsoever. Out of the pool of 17 signal peptides, nine proved to be highly suitable (Supplementary Figs. 6 and 13), when probing *S. cerevisiae* and *P. pastoris*.

The subsequent transfer of beneficial signal peptide-gene combination to *Pichia pastoris* proved successful. Nonetheless, subtle differences and preferences were shown by a signal peptide shuffling approach for *Mth*UPO and *Tte*UPO in *P. pastoris*. Interestingly, the α-factor leader signal peptide (*Sce*-Prepro), which is often used as a gold standard signal peptide for target protein secretion in *P. pastoris*^45,46^, was only identified among the top hits in combination with *Tte*UPO.

In summary almost all the 17 tested signal peptides proved to be highly functional in combination with at least one UPO gene. However, predicting a suitable signal peptide for recombinant protein secretion remains challenging. Previous work reported on the engineering of improved signal peptides by means of directed evolution to produce UPOs^10^, laccases^19^, aryl-alcohol oxidases^21^ and single-chain antibodies^18^ in *S. cerevisiae*. These improvements seem to be highly depended on the attached protein and are therefore not per se applicable to other non-related proteins. Novel approaches such as machine learning-based design of signal peptides might help to rationalise the use of SPs, but still need to be transferred to eukaryotic systems^61^. We therefore decided to build a rather diverse signal peptide panel, which can be rapidly assembled using the modular Golden Gate system and tested in a high-throughput manner in a 96-well plate setup. By employing our envisioned modular signal peptide shuffling system, we were able to further improve the production of previously described UPOs (*Aae*UPO*) and obtain multiple suitable signal peptide-gene combinations to produce novel wild-type UPOs (*Mth*UPO and *Tte*UPO).

The GFP11 detection tag proved to be an indispensable asset to distinguish secretion from activity^35^. Between different UPOs, the variation in fluorescence could be further pronounced based on different accessibilities of the split-GFP-tag. This tendency was shown for *Aae*UPO* where the TwinStrep-GFP11 tag (59 amino acids) yielded a 4-fold increased signal intensity relative to the shorter GFP11 tag (27 amino acids). In some cases, like *Cgl*UPO (Fig. 2A), the fluorescence response greatly differed from the activity depending on the employed signal peptide. This observation might be explained by different cleavage patterns at the N-terminus depending on the respective signal peptide leading to slightly altered overall folds and structures and hence activities of the mature enzyme. In the case of *Cgl*UPO, this hypothesis was strengthened by the occurrence of multiple SDS-Gel bands after enzymatic deglycosylation suggesting multiple patterns of signal peptide cleavages (Supplementary Fig. 8).

In the case of *Aae*UPO* the substrate entrance is among other motifs substantially shaped by the C-terminal helix/loop region, which also contains a crucial stabilising C278-C319 disulfide linkage^58^. Therefore, the attachment of a protein tag to the C-terminus, which we performed in all setups based on the modular design, might be detrimental to the activity. Indeed, using an *Aae*UPO* construct without the C-terminal tag resulted in an improved UPO activity by approx. 40% (Supplementary Fig. 4). The successful placement of an N-terminal tag rather than a C-terminal-modification, however, is challenging. This difficulty is due to the varying cleavage patterns at the N-terminus of the secreted proteins (see above) and also an extra Golden Gate module would be required (signal peptide - N-tag - gene) to preserve the compatibility of the system. Testing an N-terminal Strep II tag resulted in a nearly complete loss of activity (−95%) within the 96 well screening setup (Supplementary Fig. 4). Although a decreased activity is detrimental for the discovery and engineering of enzymes, the observed loss (−40%) when attaching a C-terminal tag is still tolerable, as the split-GFP signal would even in case of a loss of activity provide the signal of successful secretion and allow further enzyme characterisation. To overcome possible limitations, we constructed an additional C-terminal tag module (pAGM9121_TEV-His-GFP11), which includes a TEV protease cleavage site located in front of the His_6_-GFP11 detection and purification tag, thereby enabling the removal of the C-terminal appendix after purification and prior to activity measurements.

The utilisation of the GFP11-tag also led to the discovery and verification by subsequent peptide analysis of the peroxygenases *Gma*UPO and *Mwe*UPO—even though no enzymatic activity could be determined. Repeated shake flask production attempts in *S. cerevisiae* and *P. pastoris* did not lead to any specific absorption spectra (native and CO differential spectra) or activities. This points towards occurring problems such as extremely low secretion rates, incorrect haem incorporation or protein misfolding.

The adaptation to episomal plasmid expression in *P. pastoris* proved that the entire modular signal peptide shuffling system could be readily transferred to another yeast organism. The applicability in *P. pastoris* furthermore paves the way towards future-directed evolution enterprises entirely performed in *P. pastoris*, further streamlining the workflow from gene discovery to construct identification and large-scale protein production. In comparison to the *S. cerevisiae*-based episomal system, the *P. pastoris*-based episomal plasmid expression of *Mth*UPO retained 60% of the activity (Fig. 4E). However, there is still plenty of potential for *P. pastoris* production optimisation utilising different promoters, carbon sources, induction and co-feeding strategies^50,62^. A substantial synergistic influence of the promoter−signal peptide combination was observed, as underlined for *Tte*UPO in Fig. 4D. This observation represents an aspect that should be further investigated in detail, for example, by expanding the modular system, including an additional shuffling module for a set of promoters to achieve simultaneous signal peptide and promoter shuffling. Production of *Mth*UPO utilising the integrative plasmid led to substantially improved production when compared to the episomal counterpart. Nevertheless, the obtained interclonal variation within the integrative system is substantial, rendering the episomal plasmid expression more suitable for performing reliable high-throughput endeavours (Fig. 4C). In the case of *Mth*UPO, we observed a diminished split-GFP response compared to the respective *S. cerevisiae* construct, which might be explained through differing glycosylation patterns, as observed by SDS Gel analysis (Supplementary Figs. 8 and 14). We hypothesise that this heterogeneous glycosylation pattern might mask the C-terminal protein tag within a proportion of enzymes in a greater extent than *S. cerevisiae*, thus impeding successful interaction with the GFP 1–10 counter fragment for GFP reconstruction. Also, the removal of the C-terminal tag by endogenous proteases is a possible scenario.

UPOs have been reported to be homologously produced over the course of several weeks in bioreactors to yield *Aae*UPO, *Cra*UPO (*Coprinus radians*), *Cgl*UPO and *Mro*UPO in production titres of 9 mg/L^2^, 19 mg/L^63^, 40 mg/L^9^ and 445 mg/L^41^, respectively. Besides the time-consuming production, only the wild-type enzyme can be produced, and the overall recovery of pure protein after several purification steps are reported to be below 20%^2,41,63^. Using a heterologous yeast expression system in a shake flask format, the highest reported production titres are obtained with the engineered *Aae*UPO* in *S. cerevisiae* and *P. pastoris* (each 8 mg/L)^10,15^.

The newly discovered peroxygenases *Tte*UPO displayed unprecedented UPO expression titres in S. cerevisiae of 17 mg/L in *S. cerevisiae*. *Mth*UPO revealed a good production titre in *S. cerevisiae* (5 mg/L) and after transfer to *P. pastoris* the overall highest expression yields in *P. pastoris* with 24 mg/L could be achieved. Additionally, we could acquire high recovery of highly pure protein after one-step TwinStrep-based affinity purification^38,40^ (Supplementary Figs. 8 and 14).

To compare the results of the bioconversion setups to literature-derived data, in all experiments the secretion variant *Aae*UPO* was included, which exhibits comparable catalytic properties to the fungal wild-type enzyme *Aae*UPO and therefore allows the comparison with previously obtained analysis in the literature^10^. As there are no data available for the homologously expressed *Mth*UPO, *Tte*UPO and *Cgl*UPO, this setup was the best way to allow comparative analysis of enzymatic performances in previous heterologous and homologous setups.

The relevance of expanding the set of recombinant UPOs is reflected by the fact that *Cgl*UPO, *Mth*UPO and *Tte*UPO displayed a different substrate specificity when compared to *Aae*UPO* (Fig. 3). When testing the conversion of naphthalene within our reaction setup, *Aae*UPO* showed the highest activities with TONs of 2950, yielding 92% of 1-naphthol and generally low overoxidation to para-naphthoquinone. This 1-naphthol to naphthoquinone product distribution is in accordance with previously obtained data^60^. *Cgl*UPO, *Tte*UPO and *Mth*UPO showed lowered TONs between 720 and 1260 for 1-naphthol formation and an elevated ratio of 49–55% of the naphthoquinone product. This work on *Mth*UPO catalysed naphthalene oxidation was recently expanded by performing protein engineering and testing a range of naphthalene derivatives^64^.

The epoxidation experiments of styrene using *Aae*UPO* led in our setup to 4580 TONs and a 2% *ee*.

Previously reported data for *Aae*UPO revealed up to 10000 TONs with 4.6% *ee* when using a light-driven in situ hydrogen peroxide formation^65^. Combining *Aae*UPO* and tert-butylhydroperoxide added via a syringe pump setup resulted in 3200 TONs and 12% *ee*^66^. Within our setup, when using *Cgl*UPO similar epoxidation activities with TONs of 4120 were obtained. Most interestingly, *Mro*UPO, *Cgl*UPO and *Mth*UPO exhibited substantially higher enantioselectivities of 30, 44 and 45 % *ee*, respectively, than the reported data for *Aae*UPO and *Aae*UPO*. This indicates differences in the active site geometry of the diverse UPO set and therefore provides an interesting point for further protein engineering towards higher stereoselectivities of styrene epoxidations.

For the benzylic hydroxylation of the homologous phenylalkane row, ranging from phenylethane to phenylpentane *Aae*UPO* displayed the highest activities and selectivities using phenylethane (4540, 98% *ee*) and phenylpropane (1670, 99% *ee*) as substrates, but only traces of product for benzylic hydroxylation of phenylbutane and phenylpentane. Wild-type *Aae*UPO was previously reported to achieve TONs of 10600 and 7100 for phenylethane and phenylpropane, respectively, and excellent selectivities (>99% *ee*) in both cases^67^. Additionally, verifying the observed negative tendency of decreasing benzylic hydroxylation activity for increasing alkyl chain length *Aae*UPO* in combination with an enzymatic cascade for the in situ production of hydrogen peroxide even led up to 294700 TONs^1^.

The enzymes *Cgl*UPO, *Mth*UPO and *Tte*UPO showed an opposite selectivity when compared to *Aae*UPO* regarding benzylic hydroxylation and displayed the lowest activities for phenylethane and highest for phenylbutane and –pentane conversion, respectively. *Tte*UPO furthermore catalyses the formation of the opposite alcohol enantiomer (S) compared to the other enzymes for the conversion of phenylpropane to phenylbutane.

Good activities and excellent enantioselectivities could also be achieved when challenging the enzymes for the benzylic hydroxylation of *N*-phthalimide protected phenethylamine in case of the enzymes *Cgl*UPO and *Mth*UPO. This observation is vastly different from *Aae*UPO*, displaying no product formation and no known enantioselective conversion of substrates of similar structure.

The high UPO production yields in *P. pastoris* of *Mth*UPO enabled a proof-of-concept approach to yield the chiral alcohol product on the preparative scale with a challenging phenethylamine derivative and yielded 57% yield and 98.6% *ee*. The direct benzylic hydroxylation of phenethylamine compounds was previously reported for copper-dependent dopamine b-hydroxylases (DbH), but not on a preparative scale^68,69^. As DbHs suffer from difficult expression, their engineering towards similar substrates and higher activities is currently hampered.

To allow other researchers to harness the modular yeast system, we deposited all relevant plasmids (signal peptides, protein tags and expression plasmids) as a kit with the non-profit plasmid repository Addgene called *Yeast Secrete and Detect* Kit (Kit # 1000000166). The herein developed overall workflow for functional UPO secretion and detection can be performed within a minimal period of 15 days (Supplementary Fig. 2). Within this period, beneficial episomal constructs are identified in a 96-well high throughput system, exploiting activity measurements and protein quantification by the split-GFP assay^34,35^. Identified constructs can then be directly used for upscaling to shake flasks, one-step affinity target protein purification and subsequent bioconversion testing.

In summary, the obtained data of this study proves that the built workflow starting from a putative UPO gene, followed by identification of suitable expression constructs via signal peptide shuffling in combination with high-throughput screening in *S. cerevisiae* as well as *P. pastoris* and subsequent production upscaling can lead to highly enantioselective preparative product formations of pharmaceutically valuable building blocks.

In the future, this workflow could be applied to other UPO genes or generally genes of interest, which are suitable for production in yeast, especially for proteins that might require efficient post-translational modifications such as glycosylation and disulfide linkage. Besides target protein secretion, the constructed expression plasmids also allow for intracellular production when no signal peptide is attached. During the submission and revision process of this publication, two papers demonstrated the engineering of *Mth*UPO using the herein developed *S. cerevisiae* setup^64,70^ and one publication expanded the episomal *P. pastoris* system to a range of promoters and new UPO enzymes^71^.

## Methods

### Chemicals

Solvents were used as received without further purification. Ethyl acetate and acetone were utilised in GC ultra-grade (≥99.9%) from Carl Roth (Karlsruhe, DE). Acetonitrile was purchased from Merck (Darmstadt, DE) in gradient grade for LC ( ≥ 99.9%). Deuterated solvents for NMR spectroscopy were purchased from Deutero (Kastellaun, DE). All further reaction chemicals were purchased either from Sigma-Aldrich (Hamburg, DE), TCI Chemicals (Tokyo, JP), Merck (Darmstadt, DE), abcr (Karlsruhe, DE) or Fluka Chemika (Buchs, CH) and used as received.

### Enzymes and cultivation media

For cultivation of *E. coli* cells terrific broth (TB) media from Carl Roth (Karlsruhe, DE) was used. For cultivation of *S. cerevisiae* cells d-Galactose, Peptone and Synthetic Complete Mixture (Kaiser) Drop-Out (-URA) were purchased from Formedium (Hunstanton, GB). Yeast nitrogen base (without amino acids) and Yeast extract were purchased from Carl Roth (Karlsruhe, DE). For *P. pastoris* cultivation methanol (99.9% Chromasolv purity grade) purchased from Honeywell Chemicals (Seelze, DE) was used as additional carbon source. PNGaseF and BsaI were purchased from New England Biolabs (Ipswich, US). BbsI and FastDigest AscI were purchased from ThermoFisherScientific (Waltham, US) and T4 DNA Ligase from Promega (Madison, US).

### Oligonucleotides and gene parts

All oligonucleotides were purchased in the lowest purification grade “desalted” and minimal quantity at Eurofins Genomics (Ebersberg, DE). The *Pichia pastoris* CAT1 promoter was purchased as a gene part from Twist Bioscience (San Francisco, US). The genes of the *Aae*UPO variant *Aae*UPO*, *Gma*UPO, *Mwe*UPO and the sfGFP 1-10 gene were purchased as plasmid-cloned genes from Eurofins Genomics (Ebersberg, DE). The genes of *Cgl*UPO, *Mth*UPO and *Tte*UPO were retrieved as codon-optimised (*S. cerevisiae* codon usage) gene strands from Eurofins Genomics.

### Expression plasmid construction for *S. cerevisiae*

A Level 1 Golden Gate-based shuttle expression plasmid was constructed using a pAGT572 plasmid as backbone structure^72^, which can be propagated in *E.coli* and *S. cerevisiae*. It enables antibiotic selection (Ampicillin resistance) and yeast auxotrophy selection (URA3 marker). To enable expression of a target gene a Gal 1.3 Promoter—a truncated, modified version of the widespread GAL1 Promoter is integrated upstream and a strong DIT1 terminator downstream of the cloning acceptor site. As placeholder for a target gene sequence a lacZ cassette (approx. 600 bp) is integrated, which enables β-galactosidase-based blue/white selection of transformants based on the conversion of X-Gal. Upon digestion with BsaI the lacZ cassette is released, and a fitting open reading can be integrated in frame (e.g. Signal Peptide-Gene-C-terminal Tag) into the plasmid, thereby reconstituting a fully functional expression plasmid. The constructed expression plasmid was coined pAGT572_Nemo_2.0. Using the pAGT572 plasmid backbone and the GAL1 Promoter as units a second expression plasmid coined pAGT572_Nemo was constructed that follows the same functionality and principle but exhibits the original GAL1 promoter.

### Expression plasmid construction for *Pichia pastoris*

Two level 1 Golden Gate-based shuttle expression plasmids were constructed, which can be propagated in *E. coli* (Amp^R^) as well as *P. pastoris* (Hygromycin B^R^). To enable episomal plasmid propagation in *P. pastoris*, the plasmids were equipped with a previously described functional ARS sequence^47,73^, which was PCR amplified from *Kluveromyces lactis* genomic DNA. The plasmids exhibit the strong constitutive GAP promoter (pPAP 001) or the strong methanol inducible promoter CAT1 (pPAP002), both in combination with a strong GAP terminator (tGAP). As placeholder for a target gene sequence, a lacZ cassette is used. For the stable integration of transcription units into the *P. pastoris* genome, a third universal integrative plasmid (pPAP003) was designed. A shuttle plasmid was constructed, which can be propagated in *E. coli* (Kanamycin^R^) as well as *P. pastoris* (Hygromycin^R^). As placeholder for a target transcription unit a lacZ cassette is integrated. Upon digestion with BbsI the lacZ cassette is released and a fitting transcription unit (Promoter- ORF- Terminator) can be integrated (derived from respective pPAP001 and pPAP002 episomal plasmids as donors) into the plasmid, thereby reconstituting a fully functional integration plasmid. Several parts (GAP promoter, GAP terminator, AOX integration marker and Hygromycin B resistance marker) of the constructed plasmids were PCR amplified and derived from a previously introduced Golden Gate based *P. pastoris* assembly system, coined GoldenPiCS^44^.

### Golden Gate cloning of Level 0 standard parts

All genetic parts were cloned as individual Level 0 standard modules into the universal Level 0 acceptor plasmid pAGM9121 (Spectinomycin^R^). Therefore, three functional units were pre-defined: (a) signal peptide (contains start codon); (b) gene (lacking start and stop codon) and (c) C-terminal Protein-tag (contains stop codon). 4 bp sticky overhangs that are released upon Type II s enzyme treatment (BsaI and BbsI) and guide subsequently a correct reassembly were chosen accordingly to the nomenclature of gene assembly as described within the ModularCloning (MoClo) system^33^. An overview of the reassembly concept is provided in Supplementary Fig. 1. For the cloning of the individual modules suitable oligonucleotides were designed to allow for cloning into pAGM9121. Primers followed a general scheme (Supplementary Fig. 1). Fragments were amplified by PCR from a suitable template sequence or generated by hybridisation of two complementary oligonucleotides. PCR products were analysed as small aliquot (5 µL) by agarose gel electrophoresis for occurrence of the expected size and the remaining sample subsequently recovered and purified using a NucleoSpin^®^ Gel and PCR Clean-up Kit (Macherey-Nagel, Düren, DE). Golden Gate reactions were performed in a total volume of 15 µL. The final reaction volume contained 1-fold concentrated T4 ligase buffer (Promega, Madison, US). Prepared reaction mixtures containing ligase buffer, acceptor plasmid (20 fmol) and the corresponding insert (20 fmol) was adjusted to 13.5 µL with ddH_2_O. In a final step, the corresponding enzymes were quickly added. First, a volume of 0.5 μL of the respective restriction enzyme BbsI (5 units/µL) was added and then 1 μL (1–3 units/µL) of T4 ligase. Golden Gate reactions were performed for 3 h (37 °C) and concluded by an additional enzyme inactivation step (80 °C; 20 min). The whole Golden Gate reaction volume was used to transform chemically competent *E. coli* DH10B cells. After heat shock transformation and recovery, the mixture was plated in different quantities on selective LB Agar plates (50 μg × mL^-1^ X-Gal; 100 μg × mL^−1^ Spectinomycin; 150 μM IPTG). Based on the occurrence of the lacZ selection marker one can easily distinguish between white colonies (recombined plasmid) and empty plasmid (blue). In general, the described protocol led to several thousand recombinant colonies with a nearly absolute proportion (>99%) of recombined, white colonies. Single colonies were checked for correct insert sizes by means of colony PCR (pAGM9121 sequencing primer; Supplementary Table 1). Positively identified clones were inoculated into 4 mL of TB-Medium (100 μg × mL^−1^ Spectinomycin) and corresponding plasmid DNA prepared (NucleoSpin Plasmid Kit (Macherey-Nagel, Düren, DE)). After verification of the correct, intended insert sequence by Sanger Sequencing (Eurofins Genomics, Ebersbach, DE) respective plasmids were included for further use within the modular Golden Gate cloning approaches.

### Golden Gate cloning of expression plasmids

The expression plasmids (*S. cerevisiae*: pAGT572_Nemo and pAGT572_Nemo 2.0; *P. pastoris*: pPAP001 and pPAP002) were used as respective acceptor plasmid for the assembly of the individual tripartite open reading frames (**5´** Signal Peptide-Gene-C-terminal Tag **3´**). The individual parts were thereby derived as parts from standard level 0 plasmids (pAGM9121 backbone), which can be released from the pAGM9121 backbone upon BsaI restriction digest. Golden Gate reactions were performed in a total volume of 15 µL. The final reaction volume contained 1-fold concentrated T4 ligase buffer. Prepared reaction mixtures containing ligase buffer, the acceptor plasmid (20 fmol) and the corresponding inserts as level 0 modules (Signal Peptide, Gene, C-terminal Tag) were added to 20 fmol each and the overall volume adjusted to 13.5 µL with ddH_2_O. In the case of a signal peptide shuffling approach 17 different pAGM9121- Signal Peptide combinations were added in equimolar ratios (1.2 fmol each). In a final step, the corresponding enzymes were quickly added. First, a volume of 0.5 μL of the restriction enzyme BsaI (10 units/µL) was added and then 1 μL (1–3 units/µL) of T4 ligase. Golden Gate reactions were performed using a temperature cycling program (50x passes) between 37 °C (2 min) and 16 °C (5 min) and concluded by an additional enzyme inactivation step (80 °C; 20 min). The whole Golden Gate reaction volume was used to transform chemically competent *E. coli* DH10B cells. After heat shock transformation and recovery the mixture (approx. 320 µL) was split into two fractions, 50 µL were plated on selective LB Agar plates (+ X-Gal; 100 μg × mL^−1^ Ampicillin; + IPTG) and the remaining volume used to directly inoculate 4 mL TB Medium (+ Amp) to preserve the genetic diversity of the shuffling library. The following day the success of the Golden Gate reaction was evaluated based on the performed blue/white screening, discriminating the empty plasmid (lacZ; blue) from recombined, white colonies. In general, the described protocol for ORF assembly and signal peptide shuffling as special case led to several hundred recombinant colonies with a high proportion (>90%) of recombined, white colonies. In the case of single defined, “unshuffled” constructs single colonies were checked for correct insert sizes by means of colony PCR (using respective plasmid sequencing primer). Positively identified clones were inoculated into 4 mL of TB-Medium (+Amp) and corresponding plasmid DNA prepared (NucleoSpin Plasmid Kit (Macherey-Nagel, Düren, DE)). In the case of shuffled signal peptide constructs, plasmid DNA was prepared as a library by direct inoculation of the transformation mixture into the liquid culture and subsequent DNA isolation (see above).

### Plasmid transformation into *S. cerevisiae*

Respective single plasmids or plasmid mixtures (pAGT572_Nemo or pAGT572_Nemo 2.0 backbone) were used to transform chemically competent *S. cerevisiae* cells (INV*Sc*1 strain) by polyethylene glycol/lithium acetate transformation. INV*Sc*1 cells were prepared and stored at –80 °C in transformation buffer (15% (v/v) glycerol; 100 mM lithium acetate; 500 µM EDTA; 5 mM Tris-HCl pH 7.4) as 60 µL aliquots until usage. For transformation, an amount of 100 ng of the plasmid preparation was added to 10 µL of lachssperm DNA (10 mg/mL; Sigma Aldrich, Hamburg, DE) and mixed. This mixture was then added to a thawed aliquot of INV*Sc*1 cells on ice. 600 µL of transformation buffer (40% (v/v) polyethylene glycol 4000; 100 mM lithium acetate; 1 mM EDTA; 10 mM Tris-HCl pH 7.4) were added and the cells incubated under rigid shaking (30 °C; 850 rpm) for 30 min. Afterwards, 70 µL of pure DMSO was added and the cells incubated for a further 15 min at 42 °C without shaking. Finally, the cells were precipitated by short centrifugation, the supernatant discarded, and the cell pellet resuspended in 350 µL sterile ddH_2_O. Different volumes were plated on Synthetic Complement (SC) Drop Out plates supplemented with 2% (w/v) glucose as carbon source and lacking Uracil as an auxotrophic selection marker. Plates were incubated for at least 48 h at 30 °C till clearly background distinguishable white colonies appeared.

### Plasmid transformation into *P. pastoris*

Respective single plasmids or plasmid mixtures (pPAP001 or pPAP002 backbone) were used to transform *P. pastoris* cells (X-33 strain) by means of electroporation. Electrocompetent X-33 cells were prepared according to a condensed transformation protocol for *P. pastoris*^74^. Cells were stored in BEDS solution (10 mM bicine-NaOH pH 8.3, 3% (v/v) ethylene glycol, 5% (v/v) DMSO and 1 M sorbitol) as 60 µL aliquots (−80 °C) till further use. For the transformation of episomal plasmids 20 ng of the circular plasmid were added to one aliquot of thawed competent X-33 cells. The cell-plasmid mix was transferred to an electroporation cuvette (2 mm gap) and cooled for 10 min on ice prior to the transformation. Electroporation was performed using a Micropulser Device (Bio-Rad, Hercules, US) and using manual implemented, standardised settings (1.5 kV, 1 pulse) for all transformation setups, leading to a general pulse interval of 5.4–5.7 ms. Immediately after electroporation cells were recovered in 1 mL of ice-cold YPD-Sorbitol solution (10 g/L peptone, 5 g/L yeast extract, 500 mM sorbitol), transferred to a new reaction tube and incubated for one hour under rigid shaking (30 °C, 900 rpm) in a Thermomix device (Eppendorf, Hamburg, DE). After incubation, cells were precipitated by centrifugation (5.700 rpm, 5 min). The supernatant was discarded, and the cells resuspended in 200 µL of fresh YPD medium. 100 µL of the suspension was then plated on selective YPD Agar plates supplemented with 150 µg/mL Hygromycin B. Plates were incubated at 30 °C for at least 48 h till clearly visible colonies appeared. In general, the described setup led to the occurrence of several hundred colonies per plate. For the transformation of integrative plasmids (pPAP003 backbone) the setup was slightly modified as linearised plasmid is used for transformation. Therefore, previously prepared circular plasmid DNA was digested with AscI (Isoschizomer: SgsI). 2.5 µg of the respective plasmid DNA were mixed with 3 µL of 10x fold FastDigest Buffer, the volume adjusted to 29.5 µL using ddH_2_O and in the last step, 0.5 µL of FastDigest SgsI added. Digestion was performed overnight (16 h, 37 °C) and terminated by an enzyme inactivation step (20 min, 80 °C). Linearised plasmid DNA was then subsequently prepared according to the manufacturer instruction using a Nucleospin ^®^ Gel and PCR clean up Kit (Macherey-Nagel, Düren, DE). The transformation of *P. pastoris* was performed in a congruent manner as described before, except for using 100 ng linearised plasmid for transformation, since the overall transformation efficiency is substantially reduced in comparison to the transformation of the circular, episomal plasmid.

### Microtiter plate cultivation of *S. cerevisiae*

For peroxygenase production in microtiter plate format specialised 96 half-deep well plates were utilised. The model type CR1496c was purchased from EnzyScreen (Heemstede, NL) and plates were covered with fitting CR1396b Sandwich cover for cultivation. Plates and covers were flushed before every experiment thoroughly with 70% ethanol and air-dried under a sterile bench until usage. In each cavity, 220 µL of minimal expression medium were filled and inoculated with single, clearly separated yeast colonies using sterile toothpicks. The minimal selective expression medium (1x concentrated Synthetic complement Drop out stock solution lacking uracil; 2% (w/v) galactose; 71 mM potassium phosphate buffer pH 6.0; 3.2 mM magnesium sulfate; 3.3% (v/v) ethanol; 50 mg/L haemoglobin; 25 µg/L chloramphenicol) was freshly prepared out of sterile stock solutions immediately before each experiment, mixed and added to the cavities. After inoculation of the wells the plates were covered, mounted on CR1800 cover clamps (EnzyScreen) and incubated in a Minitron shaking incubator (Infors, Bottmingen, SUI) for 72 h (30 °C; 230 rpm). After cultivation, the cells were separated from the peroxygenase containing supernatant by centrifugation (3400 rpm; 50 min; 4 °C).

### Microtiter plate cultivation expression in *P. pastoris*

General experimental setup as before with *S. cerevisiae*. Each cavity was filled with 220 µL of buffered complex medium (BM) and inoculated with single, clearly separated yeast colonies using sterile toothpicks. Basic BM (20 g/L peptone; 10 g/L yeast extract; 100 mM potassium phosphate buffer pH 6.0; 1x YNB (3.4 g/L yeast nitrogen base without amino acids; 10 g/L ammonium sulfate); 400 µg/L biotin; 3.2 mM magnesium sulfate; 25 µg/L chloramphenicol; 50 mg/L haemoglobin; 150 µg/L Hygromycin B) was freshly prepared out of sterile stock solutions immediately before each experiment, mixed and added to the cavities. Depending on the type of utilised promoter (pPAP001: *P*_*GAP*_ and pPAP002: *P*_*CAT1*_), the BM medium was supplemented with different carbon sources for cultivation and induction, respectively. pPAP001 constructs were cultivated utilising 1.5% (w/v) of glycerol or glucose as sole carbon source. In the case of the methanol inducible CAT1 promoter, a mixed feed strategy was employed combining 0.5% (w/v) of glycerol with 1.5% (v/v) methanol. Cultivation and centrifugation was as described before for *S. cerevisiae*.

### Shake flask cultivation *S. cerevisiae*

A preculture of 50 mL of SC Drop out selection media (+ 2% (w/v) raffinose and 25 µg/L chloramphenicol) was inoculated with one single colony derived from a selection plate (SC Drop; -Uracil) and grown for 48 h (30 °C; 160 rpm; 80% humidity). This incubation typically resulted in a final OD_600nm_ of approx. 12 to 13. The main expression culture was inoculated with a starting optical density of 0.3. For large-scale peroxygenase production rich non-selective expression medium (20 g/L peptone; 10 g/L yeast extract; 2% (w/v) galactose; 71 mM potassium phosphate buffer pH 6.0; 3.2 mM magnesium sulfate; 3.3% (v/v) ethanol; 25 µg/L chloramphenicol) was utilised. Cultivation was performed in 2.5 L Ultra yield flasks (Thomson Instrument, Oceanside, US) in a final culture volume of 500 mL per flask after sealing the flask with breathable Aeraseal tape (Sigma Aldrich, Hamburg, DE) allowing for gas exchange. The main cultures were incubated for further 72 h (25 °C; 110 rpm; 80 % humidity). After cultivation, the cells were separated from the peroxygenase containing supernatant by centrifugation (4300 rpm; 35 min; 4 °C).

### Shake flask cultivation *P. pastoris*

For the large-scale protein production using shake flasks genomically integrated single constructs (pPAP 003 backbone; integration into chromosomal 3′ region of *P. pastoris* AOX1 gene) were chosen. These constructs were previously identified by screening at least 4 different colonies per individual construct within an MTP screening setup and choosing a respective production strain based on a high as possible, clearly distinguishable NBD conversion in comparison to the background control (pPAP003 empty plasmid).

Precultures were prepared in 50 mL YPD medium (+ 25 µg/L chloramphenicol) and cultivated for 48 h (30 °C; 160 rpm; 80% humidity), typically resulting in a final OD_600nm_ of approx. 17 to 19. The main expression culture was inoculated with a starting optical density of 0.3. For large-scale peroxygenase production BM-based expression media (20 g/L peptone; 10 g/L yeast extract; 100 mM potassium phosphate buffer pH 6.0; 1x YNB (3.4 g/L yeast nitrogen base without amino acids; 10 g/L ammonium sulfate); 400 µg/L biotin; 3.2 mM magnesium sulfate; 25 µg/L chloramphenicol) was utilised. In the case of constitutively expressing GAP constructs 2% (w/v) Glucose was added (BMG media) as a carbon source for Pichia growth. In the case of the methanol inducible CAT1 promoter a two-phase feeding was applied, firstly inoculating the cells into BM medium (see above) supplemented with 0.5% (w/v) glycerol as carbon source. 24 h and 48 h after inoculation 0.8% (v/v) of methanol were added as an inducer of the CAT1 promoter. Cultivation and final centrifugation were performed as described for *S. cerevisiae*.

### Supernatant ultrafiltration and protein purification

The supernatant was concentrated approx. 20-fold by means of ultrafiltration. Therefore, a Sartocon Slice 200 membrane holder (Sartorius, Göttingen, DE) was equipped with a Sartocon Slice 200 ECO Hydrosart Membrane (10 kDa nominal cut-off; Sartorius) within a self-made flow setup. The flow system for ultrafiltration was operated by an EasyLoad peristaltic pump (VWR International, Darmstadt, DE). Firstly, the cleared supernatant (1 L) was concentrated approx. 10-fold to a volume of 100 mL and 900 mL of purification binding buffer (100 mM Tris-HCl pH 8.0, 150 mM NaCl) were added as a buffer exchange step. This sample was then concentrated to a final volume of 50 mL. Protein purification was implemented utilising the C-terminal attached double Strep II Tag (WSHPQFEK), coined TwinStrep^®^ (Iba Lifesciences, Göttingen, DE). As column material, Strep-Tactin^®^XT Superflow^®^ columns (1 mL or 5 mL; Iba Lifesciences) were chosen and the flow system operated by an EasyLoad peristaltic pump (VWR). In a first step, the column was equilibrated with 5 column volumes (CVs) binding buffer. The concentrated sample (50 mL) was filter sterilised (0.2 µm syringe filter) and applied to the column with an approximate flow rate of 1 mL/min. After application, the column was washed with 7 CVs binding buffer. Elution was performed based on binding competition with biotin, therefore approx. 2 CV of elution buffer (100 mM Tris-HCl pH 8.0, 150 mM NaCl; 50 mM biotin) were applied to the column. The pooled elution fraction was then dialysed overnight (4 °C) against 5 L of storage buffer (100 mM potassium phosphate pH 7.0) using ZelluTrans dialysis tubing (6–8 kDa nominal cut-off; Carl Roth) and the recovered, dialysed sample stored at 4 °C till further use.

### Plasmid preparation of episomal plasmids from yeast

Yeast plasmids of identified clones were recovered by means of digestive Zymolase cell treatment and alkaline cell lysis. Therefore, clones were inoculated and cultivated for 48 h (30 °C; 250 rpm) in 4 mL of selection medium, in case of *S. cerevisiae* SC Drop out medium (-Uracil; 2 % (w/v) Glucose) was used and in the case of *P. pastoris* single colonies were inoculated into 4 mL of YPD ( + 150 µg/mL Hygromycin) to preserve the selection pressure. After cultivation cells were pelleted by centrifugation and 1 mL of washing buffer (10 mM EDTA NaOH; pH 8.0) added and the pellet resuspended by light vortexing. Cells were subsequently pelleted (5000 × *g*; 10 min) and the supernatant discarded. Afterwards, cells were resuspended in 600 µL of Sorbitol Buffer (1.2 M sorbitol, 10 mM CaCl_2_, 100 mM Tris-HCl pH 7.5, 35 mM β-mercaptoethanol) and 200 units of Zymolase (Sigma Aldrich, Hamburg, DE) added followed by an incubation step for 45 min (30 °C; 800 rpm) for cell wall digestion. After incubation cells were pelleted by centrifugation (2000 × *g*; 10 min) the supernatant discarded, and the plasmid preparation started with an alkaline lysis step following the manufacturer’s instructions (NucleoSpin Plasmid Kit, Macherey Nagel). In the final step, yeast-derived episomal plasmids were eluted in 25 µL elution buffer (5 mM Tris-HCl pH 8.5), and the whole eluate used to transform one aliquot of *E. coli* DH10B (transformation as described above), plating the whole transformation mix on a selective LB-Agar plate (Amp^R^). On the following day, single colonies were picked, inoculated into 4 mL of TB medium (+Amp), plasmid prepared and sent for Sanger Sequencing (Eurofins Genomics) to elucidate the respective sequence of the open reading frame.

### Thermostability measurements

Thermostability measurements of the purified enzymes were performed by Differential Scanning fluorimetry (DSF) on a Prometheus NT.48 nanoDSF instrument (NanoTemper Technologies GmbH, München, DE) in storage buffer (100 mM Tris-HCl pH 7.0). Approximately 10 µL of sample volume were loaded into a Prometheus NT.48 High Sensitivity Capillary (NanoTemper Technologies GmbH). Protein unfolding was subsequently monitored by following the ratio of intrinsic protein tyrosine and tryptophan fluorescence at 350 nm to 330 nm over time, increasing the temperature from 20 °C to 95 °C with a heating ramp of 1 °C per minute. The melting temperature corresponds to the maximum of the first derivative of the 350/330 nm ratio. All measurements were performed at least in triplicates.

### Split-GFP assay

Protein normalisation was performed employing the principle of a split GFP normalisation assay as described by Santos-Aberturas et al.^35^ with slight modifications. The GFP fluorescence complementation fragment sfGFP 1–10 was cloned into the Golden Mutagenesis plasmid pAGM22082_cRed^32^ for T7 promoter controlled expression in *E. coli* (BL 21 DE3 strain). The sfGFP 1–10 fragment was prepared as an inclusion body preparation according to the previous reports^35^. For measurement, a 96 well Nunc MaxiSorp Fluorescence plate (ThermoFisherScientific, Waltham, US) was blocked (25 min, light shaking) with 180 µL of BSA blocking buffer (100 mM Tris-HCl pH 7.4, 100 mM NaCl, 10% (v/v) glycerol, 0.5% (w/v) BSA). The blocking solution was discarded and 20 µL of the yeast media supernatant (*S. cerevisiae* or *P. pastoris*) derived from the peroxygenase expression plates added. A 10 mL aliquot of the sfGFP 1–10 complementation fragment (storage: −80 °C) was quickly thawed in a water bath and diluted 1x fold into ice-cold TNG buffer (100 mM Tris-HCl pH 7.4, 100 mM NaCl, 10% (v/v) glycerol) and 180 µL of this screening solution added to each well. Immediate fluorescence values (GFP fluorophore: excitation wavelength: 485 nm; emission wavelength: 535 nm; top read mode) were measured using a 96 well plate fluorescence reader Spark 10 M (TECAN, Grödig, AT), setting an empty plasmid control well as 10% of the overall signal intensity (well calculated gain). After storage of the plate for at least one up to three nights (at 4 °C) final fluorescence values were measured in a comparable manner. Protein quantities were then normalised based on the relative fluorescence increase of each respective well (differential values) and in comparison, to the empty plasmid backbone.

### DMP assay

The use of 2,6-Dimethoxyphenol (DMP) as a suitable microtiter plate substrate for the measurement of peroxygenase catalysed conversion to the colorimetric product coerulignone has been described before^60^. The described conditions have been adapted with slight modifications. In brief, 20 µL of peroxygenase containing supernatant were transferred to a transparent polypropylene 96-well screening plate (Greiner Bio-One, Kremsmünster, AT) and 180 µL of screening solution (final: 100 mM potassium phosphate pH 6.0; 3 mM 2,6-Dimethoxyphenol; 1 mM hydrogen peroxide) added. Absorption values (λ: 469 nm) of each well were immediately measured after addition in a kinetic mode (measurement interval: 30 s) over a duration of 5 min utilising the 96-well microtiter plate reader SpectraMax M5 (Molecular Devices, San José, US). Slope values of absorption increase corresponding to coerulignone formation were obtained, paying special attention to the linearity of the observed slope to obtain reliable relative DMP conversion values for comparison of the respective wells.

### NBD assay

The use of 5-nitro-1,3-benzodioxole (NBD) as a suitable microtiter plate substrate for the measurement of peroxygenase catalysed conversion to the colorimetric product 4-Nitrocatechol has been described before^37,75^. Screening as described above for DMP but adding 180 µL of screening solution (final: 100 mM potassium phosphate pH 6.0; 1 mM NBD; 1 mM hydrogen peroxide; 12% (v/v) acetonitrile). Absorption values (λ: 425 nm) of each well were immediately measured after addition in a kinetic mode (measurement interval: 30 s) over a duration of 5 min. Slope values of absorption increase corresponding to 4-nitrocatechol formation were obtained, paying special attention to the linearity of the observed slope to obtain reliable relative NBD conversion values for comparison of the respective wells.

### Resting-state absorption and haem CO complex measurements

The pooled and dialysed elution fractions (100 mM potassium phosphate pH 7.0) were subsequently used to record absorption spectra of the respective enzymes (*Mro*UPO, *Cgl*UPO, *Mth*UPO, *Tte*UPO) in their native, resting state (ferric iron; Fe^3+^). For all measurements, a QS High precision Quartz Cell cuvette (Hellma Analytics, Müllheim, DE) with a path length of 10 mm was used. Spectra were recorded on a Biospectrometer Basic device (Eppendorf, Hamburg, DE) in the spectral range from 250 to 600 nm (interval: 1 nm) and subtracting the utilised storage buffer (100 mM potassium phosphate pH 7.0) as previous blank measurement. Haem carbon dioxide spectra (CO assay) were recorded after reducing the haem iron to its ferrous form (Fe^2+^). Therefore, a spatula tip of sodium dithionite as the reducing agent was added to 1 mL of a respective enzyme fraction (see above) and mixed thoroughly till complete dissolution. This sample was immediately flushed with a constant carbon dioxide flow for 2 min (approx. 1 bubble/sec) to obtain the thiolate-haem carbon dioxide complex. The sample was immediately transferred to a cuvette and absorption measured as described above. The CO assay was also employed for the measurement of peroxygenase concentrations in the concentrated *P. pastoris* supernatant obtained after ultrafiltration. In this case, the supernatant was 10-fold diluted with potassium phosphate buffer (100 mM, pH 7.0). A spatula tip of sodium dithionite was then added to 2 mL of the diluted supernatant sample. After dividing the respective sample into two parts of 1 mL, one part was treated with carbon monoxide for 2 min as described above, and the CO untreated sample is used as a blank reference. Absorption measurements were performed by UV/Vis spectroscopy using a JASCO V-770 Spectrophotometer (JASCO Deutschland GmbH, Pfungstadt). The CO absorption maximum was measured at 444 nm, and a reference absorption wavelength was measured at 490 nm. For calculation, an extinction coefficient of 91,000 M^−1^ cm^−1^ was used, which appears to be generally valid for most haem-thiolate enzymes according to literature^76^. The enzyme concentration in the supernatant was then calculated using the formula:

$${\rm{c}}[{{\upmu}} {\rm{M}}]={\rm{dilution}}\,{\rm{factor}}\times \frac{{{\rm{A}}}_{444{\rm{nm}}}-{{\rm{A}}}_{490{\rm{nm}}}}{0.091{{\upmu}} {{\rm{M}}}^{-1}{{\rm{cm}}}^{-1}}$$

### pH range of NBD conversion

pH dependency of NBD conversion of the respective enzymes was investigated using different buffer system in the range between pH 2.0 and 11.0 (even numbers only). Each buffer was prepared as a 100 mM stock solution, potassium phosphate buffer was used for the pH values 2.0, 7.0 and 8.0. Sodium citrate was used in the range of pH 3.0 to 6.0 and Tris-HCl was used in the range of pH 8.0 to 11.0. Purified enzyme solutions (100 mM potassium phosphate pH 7.0) were diluted 10 to 20x fold in ddH_2_0 prior to the measurements leading to weakly buffered solutions as screening samples. The NBD assay was then performed as described before, mixing 20 µL of the enzyme dilution with 180 µL screening solution (87 mM corresponding buffer pH x; 500 µM NBD; 1 mM H_2_O_2_). All samples were measured as three biological replicates. Due to the strong pH-dependency of the molar extinction coefficient of the corresponding detected product 4-nitrocatechol a normalisation was performed. Therefore, the product 4-Nitrocatechol was prepared as 10 mM stock solution dissolved in acetonitrile and diluted into 990 µl of the corresponding screening buffer (final concentration: 10 µM) and after 5 min an absorption spectrum in the interval of 400 to 600 nm (Biospectrometer Basic device) recorded. Calculation of the correction factor of the respective samples (pH 2.0 to pH 11.0) was then performed regarding the utilised measurement wavelength of 425 nm. Finally, in consideration of the obtained pH correction factor, individual activity values derived from the respective measured absorption values were calculated.

### Protein concentration determination and purification yield

Protein concentrations of the respective protein samples were determined after dialysis of the elution fractions (storage buffer: 100 mM potassium phosphate pH 7.0). In this regard, the colorimetric BCA assay was utilised, employing a Pierce™ BCA Protein Assay Kit (ThermoFisherScientific, Waltham, US) following the instructions of the manufacturer. Samples were measured in biological triplicates (25 µL of a previously diluted sample) and concentrations calculated based on a previously performed calibration curve using BSA (0–1000 µg/mL) as reference protein. To determine the overall yield of enzyme production per litre of culture volume, the determined concentration in the elution fraction was extrapolated to the overall NBD activity of the sample after ultrafiltration (column load). This calculation is performed since NBD is a highly specific substrate for peroxygenase activity, comparable background samples processed in a similar manner but using empty plasmid controls did not show any measurable conversion of NBD. Samples of every purification step (load, flow-through, wash and elution fraction) were collected, and NBD conversion rates of the respective fractions measured immediately after purification. In the case of non-complete binding of the enzyme fraction (remaining NBD activity in flow-through fraction) this remaining non-bound enzyme amount was taken into consideration for calculation for the overall volumetric production yield. The via BCA assay determined protein concentration of the elution fraction was extrapolated to the activity of the respective non-bond fraction, assuming a constant specific enzyme activity for NBD conversion and considering the volumes of the respective fractions, leading to an approximate enzyme titre per litre.

### SDS gel analysis and PNGaseF treatment

Obtained elution fractions of the respective UPO enzymes were analysed for the apparent molecular weight and overall purity after the performed one step TwinStrep purification by means of SDS PAGE. Therefore, samples of the column load (after ultrafiltration; see above), elution fractions after dialysis and deglycosylated elution fraction samples were analysed on self-casted SDS PAGE (10 or 12% of acrylamide) utilising a Bio-Rad (Hercules, US) Mini-Protean^®^ Gel electrophoresis system. A defined PageRuler Prestained Protein Ladder (ThermoFisherScientific, Waltham, US) was included, covering a MW range between 10 and 180 kDa. Proteins were visualised using a colloidal Coomassie G-250 staining solution. To obtain N-type deglycosylated protein samples, elution fractions were enzymatically treated with Peptide-N-Glycosidase F (PNGaseF) from *Flavobacterium meningosepticum*, which is capable of cleaving Asparagine linked high mannose type glycan structures as typically occurring in *P. pastoris* and *S. cerevisiae* derived glycosylation patterns. Therefore, 45 µL of a respective elution fraction was mixed with 5 µL of denaturing Buffer (final 0.5% SDS; 40 mM DTT) and denatured for 10 min (100 °C). After a cooling step to room temperature 6 µL of NP-40 solution (final: 1 %) and 6 µL of GlycoBuffer2 (500 mM sodium phosphate; pH 7.5) were added and the solution thoroughly mixed. Finally, 1 µL of PNGaseF (New England Biolabs, Ipswich, US) was added and the sample incubated under light shaking (37 °C) for 3 h. After incubation, the sample was prepared for further analysis by adding 5x fold SDS sample buffer and subsequent SDS PAGE analysis executed as described before. In the case of native deglycosylation, 90 µL of enzyme sample were mixed with 10 µL of GlycoBuffer2 (500 mM Sodium Phosphate; pH 7.5) and 1 µL of PNGaseF added. The mixture was incubated at 37 °C in a thermal PCR cycler (24 or 48 h) and subsequently analysed for UPO activity in comparison with an equally treated sample (without PNGAseF addition) by means of the NBD assay (see above).

### Protein identification by MS

Protein samples after protein purification (in 100 mM Tris-HCl pH 8.0, 150 mM NaCl; 50 mM biotin) were enzymatically digested with trypsin and desalted according to ref. ^77^. The resulting peptides were separated using C18 reverse-phase chemistry employing a pre-column (EASY column SC001, length 2 cm, ID 100 μm, particle size 5 μm) in line with an EASY column SC200 with a length of 10 cm, an inner diameter (ID) of 75 μm and a particle size of 3 μm on an EASY-nLC II (all from Thermo Fisher Scientific). Peptides were eluted into a Nanospray Flex ion source (Thermo Fisher Scientific) with a 60 min gradient increasing from 5% to 40% acetonitrile in ddH_2_O with a flow rate of 300 nL/min and electrosprayed into an Orbitrap Velos Pro mass spectrometer (Thermo Fisher Scientific). The source voltage was set to 1.9 kV, the S Lens RF level to 50%. The delta multipole offset was -7.00. The AGC target value was set to 1e06 and the maximum injection time (max IT) to 500 ms in the Orbitrap. The parameters were set to 3e04 and 50 ms in the LTQ with an isolation width of 2 Da for precursor isolation and MS/MS scanning. Peptides were analysed using a Top 10 DDA scan strategy employing HCD fragmentation with stepped collision energies (normalised collision energy 40, 3 collision energy steps, width 15). MS/MS spectra were used to search the TAIR10 database (ftp://ftp.arabidopsis.org, 35394 sequences, 14486974 residues) amended with target protein sequences with the Mascot software v.2.5 linked to Proteome Discoverer v.2.1. The enzyme specificity was set to trypsin, and two missed cleavages were tolerated. Carbamidomethylation of cysteine was set as a fixed modification and oxidation of methionine. Searches were performed with enzyme specificity set to trypsin and semi-trypsin to identify truncated protein N-termini. The precursor tolerance was set to 7 ppm, and the product ion mass tolerance was set to 0.8 Da. A decoy database search was performed to determine the peptide spectral match (PSM) and peptide identification false discovery rates (FDR). PSM, peptide and protein identifications surpassing respective FDR thresholds of *q* < 0.01 were accepted.

### UPO bioconversions for subsequent GC-MS and chiral HPLC analytics

For the tested hydroxylation (naphthalene, phenylethane, -propane, -butane and -pentane) and epoxidation (styrene) reactions, purified UPOs enzyme samples (stored in 100 mM potassium phosphate; pH 7.0) produced in *S. cerevisiae* were used. Respective reactions (total volume: 400 µL) were performed as biological triplicates in 100 mM potassium phosphate (pH 7.0) containing 100 nM of UPO, 1 mM of the respective substrate and 500 µM H_2_O_2_. The substrate was prior dissolved in pure acetone (20 mM stock solution) yielding a 5% (v/v) co-solvent ratio in the final reaction mixture. Reactions were performed for 60 min (25 °C, 850 rpm) and subsequently quenched by the addition of 400 µl ethyl acetate (internal standard: 1 mM ethyl benzoate). Extraction was accomplished by 30 s of vigorous vortexing, followed by brief centrifugation (1 min, 8400 rpm). The organic layer was then utilised for respective GC-MS measurements as described in Supplementary Table 7. In the case of the hydroxylation reaction of *N*-phthaloyl-phenylethyl amine, purified UPOs enzyme samples (stored in 100 mM potassium phosphate, pH 7.0.) previously produced in *P. pastoris* were used. Reactions (total volume: 500 µL) were performed as biological triplicates in 100 mM potassium phosphate (pH 7.0) containing 100 nM of the respective UPO, 250 µM of the substrate *N*-phthaloyl-phenylethyl amine and 250 µM H_2_O_2_. The substrate was prior dissolved in pure acetone (5 mM stock solution) yielding a 5% (v/v) co-solvent ratio in the final reaction mixture. Reactions were performed for 60 min (30 °C, 850 rpm) and subsequently quenched by the addition of 650 µL ethyl acetate (internal standard: 1 mM ethyl benzoate). Extraction was accomplished by 30 s of vigorous vortexing, followed by brief centrifugation (1 min, 8400 rpm). 200 µL of the resulting organic layer were utilised for GC-MS measurements. The remaining organic solvent was evaporated using a mild nitrogen stream, the precipitate resolved in 200 µL isopropanol and utilised for chiral HPLC measurements. For the larger scale hydroxylation reaction of *N*-phthaloyl-phenylethyl amine with *Cgl*UPO general procedures were followed as described above with some slight alterations. In contrast to the previous small-scale reaction (500 µL), within this approach, ten reactions (each total volume: 1 mL) were performed in parallel in 100 mM potassium phosphate (pH 7.0) containing 250 nM *Cgl*UPO, 250 µM substrate and 250 µM H_2_O_2_. Reactions were performed for 60 min (30 °C, 850 rpm) and subsequently quenched by the addition of 1 mL ethyl acetate to each reaction vial. Extraction was accomplished by 30 s of vigorous vortexing, followed by brief centrifugation (1 min, 8400 rpm). The organic layers of all samples were combined, and the solvent was gradually evaporated using a mild nitrogen stream. The precipitate was then resolved in 200 µL isopropanol and utilised for chiral HPLC measurements (Supplementary Figs. 18–20).

### Achiral gas chromatography–mass spectrometry (GC–MS)

Measurements were performed on a Shimadzu GCMS-QP2010 Ultra instrument (Shimadzu, Kyoto, JP) using a SH-Rxi-5Sil MS column (30 m x 0.25 mm, 0.25 µm film, Shimadzu, Kyoto, JP) or OPTIMA 5MS Accent column (25 m x 0.20 mm, 0.20 µm film, Macherey-Nagel, Düren, DE) and helium as carrier gas. 1 µl of each sample was injected splitless with an injection temperature of 280 °C. The split/splitless uniliner inlets (3.5 mm, 5.0 × 95 mm for Shimadzu GCs, deactivated wool) from Restek (Bad Homburg, DE) were utilised and regenerated if needed by CS-Chromatography (Langerwehe, DE). The temperature program was adjusted, as shown in Supplementary Table 7. The interface temperature was set to 290 °C. Ionisation was obtained by electron impact with a voltage of 70 V, and the temperature of the ion source was 250 °C. The MS is equipped with dual-stage turbomolecular pumps and a quadrupole enabling a selected ion monitoring acquisition mode (SIM mode). Calibration and quantification were implemented in SIM mode with the corresponding *m/z* traces, as shown in Supplementary Table 7. The detector voltage of the secondary electron multiplier was adjusted in relation to the tuning results with perfluorotributylamine. The GC–MS parameter was controlled with GCMS Real Time Analysis, and for data evaluation, GCMS Postrun Analysis (GCMSsolution Version 4.45, Shimadzu, Kyoto, JP) was used.

### Chiral gas chromatography–mass spectrometry (GC–MS)

Measurements were performed on a Shimadzu GCMS-QP2020 NX instrument (Shimadzu, Kyoto, JP) with a Lipodex E column (25 m x 0.25 mm, Macherey-Nagel, Düren, DE) and helium as carrier gas. 1 µl of each sample was injected splitless with an OPTIC-4 (Shimadzu, Kyoto, JP) injector utilising a temperature profile in the liner (35 °C, 1 °C/s to 220 °C hold 115 s). The column temperature program was adjusted as shown in Supplementary Table 7. The interface temperature was set to 200 °C. Ionisation was obtained by electron impact with a voltage of 70 V, and the temperature of the ion source was 250 °C. The MS is equipped with dual stage turbomolecular pumps and a quadrupole enabling a selected ion monitoring acquisition mode (SIM mode). Calibration and quantification were implemented in SIM mode with the corresponding *m/z* traces, as shown in Supplementary Table 7. The detector voltage of the secondary electron multiplier was adjusted in relation to the tuning results with perfluorotributylamine. The GC–MS parameters were controlled with GCMS Real Time Analysis, and for data evaluation GCMS Postrun Analysis (GCMSsolution Version 4.45, Shimadzu, Kyoto, JP) was used.

### GC–MS calibration curves

For product quantification, calibration curves were created as depicted in Supplementary Fig. 15. The quantification was achieved in Scan mode (*N*-(2-hydroxy-2-phenylethyl) phthalimide) or SIM mode (all other substrates) whereby each concentration data point was measured as triplicates and correlated to an internal standard (IS). The final product concentration was adjusted in 100 mM potassium phosphate buffer (pH 7.0) with the corresponding stock solutions in acetone yielding to 5% (v/v) final co-solvent proportion in the buffer system. Extraction was achieved adding 650 µL (*N*-(2-hydroxy-2-phenylethyl)phthalimide) or 400 µL (all other substrates) of ethyl acetate (containing 1 mM of the internal standard) and vortexing for 30 s, followed by brief centrifugation (1 min, 8400 rpm). The organic layer was utilised for GC–MS measurements applying the corresponding temperature program as listed in Supplementary Table 7. For enantiomeric product identification corresponding R-enantiomer standards were utilised (Supplementary Fig. 16).

### Preparative work

#### *N*-Phthaloyl-phenylethyl amine

Phthalic anhydride (0.59 g, 4.0 mmol), phenylethyl amine (0.51 mL, 4.0 mmol) were dissolved in dichloromethane (40 mL) at room temperature. Molecular sieves (4 Å pore diameter) and triethylamine (2.0 mL, 14.5 mmol) were added, and the reaction mixture was refluxed for 36 h. After the reaction was completed (TLC control) the mixture was filtered, and the solvent was evaporated under reduced pressure. The residue was dissolved in ethyl acetate, washed with sodium bicarbonate solution and water and dried over sodium sulphate. After filtration, the product was obtained under reduced pressure to yield 0.31 g (80%) as an orange solid. No further purification was necessary.

^**1**^**H-NMR** (400 MHz, CDCl_3_): δ 7.83 (dd, *J* 5.4, 3.1 Hz, 2H), 7.70 (dd, *J* 5.5, 3.0 Hz, 2H), 7.32 – 7.17 (m, 5H), 3.96 – 3.90 (m, 2H), 3.02 – 2.95 (m, 2H) ppm;

^**13**^**C-NMR** (100 MHz, CDCl_3_): δ 168.15, 137.99, 133.88, 132.06, 128.83, 128.53, 126.62, 123.19, 39.27, 34.60 ppm;

**MS** (ESI, MeOH): m/z 274.1 ([M + Na]^+^), calcd: 251.09.

#### (R,S)-2-*N*-Phthaloyl-1-phenylethanol

Phthalic anhydride (0.30 g, 2.0 mmol) and 2-amino-1-phenylethanol (0.27 g, 2.0 mmol) were placed into a microwave vessel under stirring (magnetic). The vessel was heated to 150 °C for 30 min in the microwave reactor. After cooling to room temperature, the product was washed with HCl (1 M, 20 mL) and recrystallised from dichloromethane/n-hexane to yield 0.47 g (89%) as colourless crystals.

^**1**^**H-NMR** (400 MHz, CDCl_3_): δ 7.82 (dd, *J* 5.4, 3.1 Hz, 2H), 7.70 (dd, *J* 5.5, 3.0 Hz, 2H), 7.48 – 7.40 (m, 2H), 7.39 – 7.27 (m, 3H), 5.06 (dt, *J* 8.6, 4.2 Hz, 1H), 4.07 – 3.85 (m, 2H), 3.03 (d, *J* 5.0 Hz, 1H) ppm;

^**13**^**C-NMR** (100 MHz, CDCl_3_): δ 168.69, 141.02, 134.06, 131.81, 128.53, 128.03, 125.83, 123.39, 72.47, 45.67 ppm;

**MS** (ESI, MeOH): m/z 268.1 ([M + H]^+^), 290.0 ([M + Na]^+^), calcd: 267.09.

#### (S)-( + )-2-*N*-Phthaloyl-1-phenylethanol (chemical conversion)

Phthalic anhydride (0.30 g, 2.0 mmol) and (S)-( + )-2-amino-1-phenylethanol (0.27 g, 2.0 mmol) were placed into a microwave vessel under stirring (magnetic). The vessel was heated to 150 °C for 30 min in the microwave reactor. After cooling to room temperature, the product was washed with HCl (1 M, 20 mL) and recrystallised from dichloromethane/n-hexane to yield 0.44 g (82%) as colourless crystals.

^**1**^**H-NMR** (400 MHz, CDCl_3_): δ 7.85 (dd, *J* 5.5, 3.1 Hz, 2H), 7.73 (dd, *J* 5.5, 3.1 Hz, 2H), 7.50 – 7.27 (m, 5H), 5.19 – 4.96 (m, 1H), 4.10 – 3.86 (m, 2H), 2.97 – 2.78 (m, 1H) ppm;

^**13**^**C-NMR** (100 MHz, CDCl_3_): δ 168.75, 141.05, 134.13, 131.88, 128.60, 128.11, 125.86, 123.46, 72.68, 45.76 ppm;

**MS** (ESI, MeOH): m/z 289.9 ([M + Na]^+^), calcd: 267.09;

$${[a]}_{20}^{D}+23.9({\rm{c}}0.75,{\rm{CHCl}}_3).$$

#### (S)-( + )-2-*N*-Phthaloyl-1-phenylethanol (enzymatic conversion)

*N*-Phthaloyl-phenylethyl amine (15.8 mg, 62.9 µmol) was dissolved in acetone (15 mL) and poured into a solution of potassium phosphate buffer (100 mM, 263 mL, pH 7.0), hydrogen peroxide (210 μM, 3.2 mL) and *Mth*UPO (250 nM, 15 mL). The solution (total: 300 mL) was stirred at 30 °C for 1 h. Afterwards the mixture was extracted using ethyl acetate (3 × 60 ml). The organic phase was washed with brine, dried with sodium sulphate, filtered and concentrated under reduced pressure. The crude product was purified by column chromatography on silica gel using dichloromethane/ethyl acetate with 1% formic acid (1/5 → 1/1) obtaining 9.70 mg (57 %) (S)-( + )-2-*N*-Phthaloyl-1-phenylethanol as a pale-yellow solid.

^**1**^**H-NMR** (400 MHz, CDCl_3_): *δ* 7.86 (dd, *J* 5.5, 3.1 Hz, 2H), 7.73 (dd, *J* 5.5, 3.0 Hz, 2H), 7.49 – 7.43 (m, 2H), 7.42 – 7.27 (m, 3H), 5.08 (dd, *J* 8.7, 3.6 Hz, 1H), 4.11 – 3.86 (m, 2H), 2.83 (s, 1H) ppm;

^**13**^**C-NMR** (100 MHz, CDCl_3_): *δ* 168.76, 141.04, 134.14, 131.89, 128.62, 128.13, 125.86, 123.48, 72.72, 45.77 ppm;

**MS** (ESI, MeOH): *m/z* 289.9 ([M + Na]^+^), calcd: 267.09;

$${[a]}_{20}^{D}+21.0({\rm{c}}1.55,{\rm{CHCl}}_3).$$

#### *N*-Phthaloyl-2-oxo-phenylethyl amine

(R,S)-*N*-Phthaloyl-phenylethanol (0.18 g, 0.67 mmol) was dissolved in dimethyl sulfoxide (6 mL) at room temperature. Under ice cooling, acetic anhydride (1.2 mL) was added, and the reaction mixture was stirred for 16 h at room temperature. After the reaction was completed (TLC control) the mixture was quenched with ethyl acetate (20 mL), and the mixture was washed with sodium perchlorate solution (6 %), sodium thiosulfate solution (10 %) and brine and dried over sodium sulfate. After filtration, the product was obtained under reduced pressure to yield 0.15 g (84 %) as a colourless solid. No further purification was necessary.

^**1**^**H-NMR** (400 MHz, CDCl_3_): *δ* 8.06 – 7.98 (m, 2H), 7.91 (dd, *J* = 5.5, 3.0 Hz, 2H), 7.76 (dd, *J* = 5.5, 3.0 Hz, 2H), 7.69 – 7.48 (m, 3H), 5.14 (s, 2H) ppm;

^**13**^**C-NMR** (100 MHz, CDCl_3_): *δ* 190.94, 167.88, 134.43, 134.11, 134.02, 132.25, 128.89, 128.14, 123.55, 44.19 ppm;

**MS** (ESI, MeOH): *m/z* 288.1 ([M + Na]^+^), calcd: 265.07.

### Column and analytic thin layer chromatography

All solvents for column chromatography were purchased from Merck Millipore (Darmstadt, DE) and distilled prior to use. Column chromatography was carried out using Merck silica gel 60 (40–63 µm). For analytic thin layer chromatography, Merck TLC silica gel 60 F254 aluminium sheets were used. Compounds were visualised by using UV light (254/366 nm).

### Nuclear magnetic resonance (NMR)

NMR spectra were recorded using a 400 MHz Agilent DD2 400 NMR spectrometer at 25 °C. The chemical shifts of 1H NMR spectra are referenced on the signal of the internal standard tetramethylsilane (*δ* = 0.000 ppm). Chemical shifts of 13 C NMR spectra are referenced on the solvent residual signals of CDCl_3_ (*δ* = 77.000 ppm).

### Electrospray ionisation mass spectrometry (ESI-MS)

ESI mass spectra were recorded on an API3200 Triple Quadrupole mass spectrometer (AB Sciex) equipped with an electrospray ion source (positive spray voltage 5.5 kV, negative spray voltage 4.5 kV, source heater temperature 400 °C).

### Specific optical rotation

Specific optical rotations of compounds were recorded on a P-2000 Digital Polarimeter (JASCO, Pfungstadt, DE) utilising a wavelength of 589 nm.

### Chiral HPLC

HPLC chromatograms were recorded on an Agilent High Performance LC (Agilent Technologies, Waldbronn, DE). The used chiral column material was Chiralpak AS-H HPLC (Daicel, Tokyo, JP) (25 cm × 4.6 mm). Substances were dissolved in HPLC-grade isopropanol prior to analysis, and a sample volume of 5 µL injected. The eluent (20% isopropanol, 80% *n*-hexane) was used in a flow rate of 1 mL/min with the runtime of 30 min at 30 °C.

### Microwave reactions

Microwave reactions were carried out using an Initiator + device (Biotage, Düsseldorf, DE).

### Reporting summary

Further information on research design is available in the Nature Research Reporting Summary linked to this article.

## Supplementary information

Supplemental Information

Description of Additional Supplementary Files

Supplementary Data 1

Reporting Summary

## Data Availability

The authors declare that the data supporting the findings of this study are available within the paper and its Supplementary Information files. Source data is provided as Supplementary Data 1.
