# Supplementary material for: A modular two yeast species secretion system for the production and preparative application of unspecific peroxygenases
Source: Commun Biol. 2021 May 12;4:562. doi: 10.1038/s42003-021-02076-3 (PMC8115255; doi:10.1038/s42003-021-02076-3)
Supplement: Supplementary file 1 — Supplemental Information [file 42003_2021_2076_MOESM1_ESM.pdf]

Supplementary Information

A modular two yeast species secretion system for the production and preparative application of unspecific peroxygenases

Pascal Püllmann[1], Anja Knorrscheidt[1], Judith Münch[1], Paul R. Palme[1], Wolfgang Hoehenwarter[1], Sylvestre Marillonnet[1], Miguel Alcalde[3], Bernhard Westermann[1,2], Martin J. Weissenborn[*1,2]

*Corresponding author. Email: martin.weissenborn@ipb-halle.de

Table of Contents

I. Supplementary Figures 2

II. Supplementary Tables..26

III. NMR spectra 37

Supplementary Fig. 1 Principle of modular UPO shuffling system

Supplementary Fig. 2 Schematic overview of the developed workflow

Supplementary Fig. 3 Comparison of C-terminal GFP11 and TwinStrep-GFP11 constructs

Supplementary Fig. 4 Comparison of differently tagged and untagged *Gma*-UPO-*Aae*UPO* constructs

Supplementary Fig. 5 Identification of suitable signal peptides for *Mth*UPO secretion in *S. cerevisiae*

Supplementary Fig. 6 Identification of suitable signal peptides for *Tte*UPO secretion in S. cerevisiae

Supplementary Fig. 7 CO absorption measurements

Supplementary Fig. 8 SDS PAGE analysis of UPOs produced in *S. cerevisiae*

Supplementary Fig. 9 Enzymatic activity of UPOs derived from *S. cerevisiae* after deglycosylation

Supplementary Fig. 10 Comparison of UPO production from episomal and integrative constructs

Supplementary Fig. 11 Comparison colony shape and size episomal and integrative constructs

Supplementary Fig. 12 Identification of suitable signal peptides for *Mth*UPO secretion in *P. pastoris*

Supplementary Fig. 13 Identification of suitable signal peptides for *Tte*UPO secretion in *P. pastoris*

Supplementary Fig. 14 SDS PAGE analysis of UPOs derived from *P. pastoris*

Supplementary Fig. 15 Calibration curves GC-MS

Supplementary Fig. 16 Enantiomeric separation 1-phenylethyl alcohol products

Supplementary Fig. 17 Scan Mode measurements of UPO catalysed Phenyl alkane hydroxylation

Supplementary Fig. 18 SIM Mode measurements of UPO catalysed *N*-Phthaloyl-phenylethyl amine conversion

Supplementary Fig. 19 Scan Mode measurements of UPO catalysed *N*-Phthaloyl-phenylethyl amine conversion

Supplementary Fig. 20 Chiral HPLC analysis of UPO catalysed *N*-Phthaloyl-phenylethyl amine conversion

Supplementary Table 1 Overview of oligonucleotides for sequencing of the created plasmids
Supplementary Table 2 Overview of utilised strains for cloning and protein production purposes
Supplementary Table 3 Overview of all created and utilised plasmids within this study
Supplementary Table 4 Employed signal peptides, their origins and amino acid sequences
Supplementary Table 5 Employed C-terminal Tags, their amino acid sequence and purpose
Supplementary Table 6 Protein identification by mass spectroscopy
Supplementary Table 7 Measurement parameters for achiral and chiral GC-MS
Supplementary Table 8 Protein coverage of the protein digest and MS analysis
Supplementary Table 9 Enantiomeric excess determination for UPO catalysed N-Phthaloyl-phenylethyl amine conversion
Supplementary Table 10 Overview of protein sequences and sequence alignments

I. Supplementary Figures

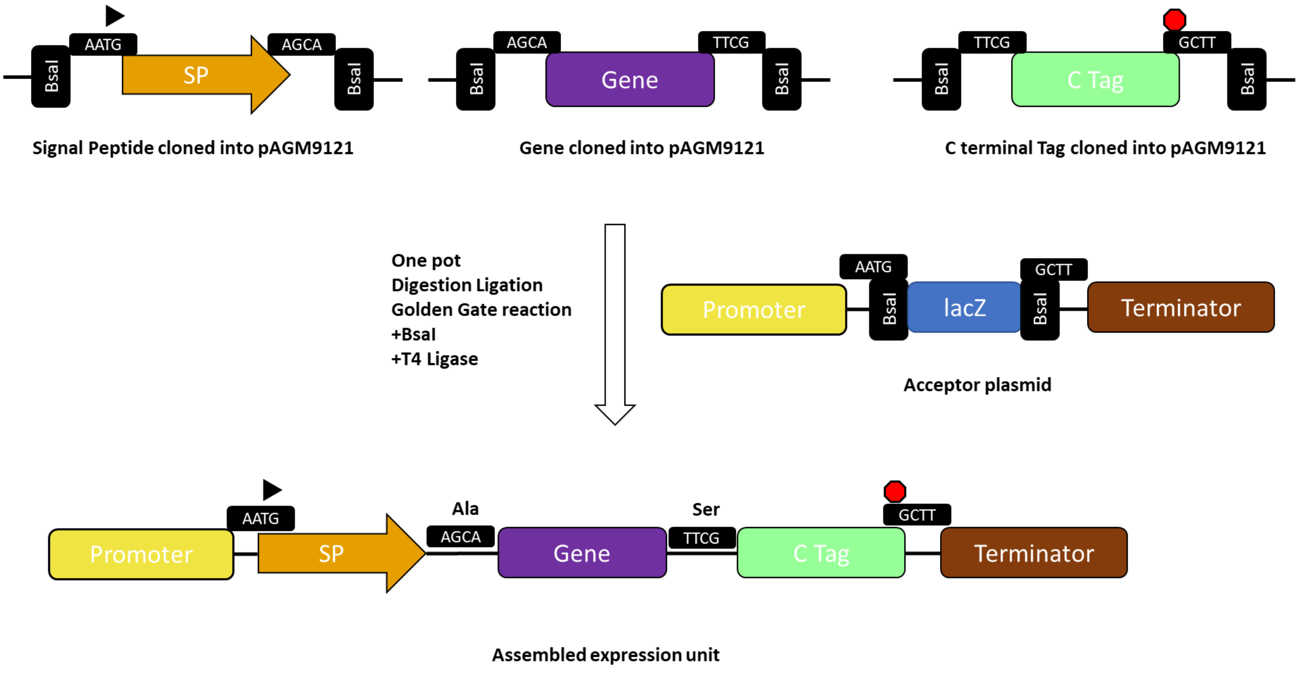

Primer design example for adding a gene unit into the modular *Yeast Secret and Detect* system (clone as Level 0 module into pAGM9121):

Forward Primer:

TTGAAGACAACTCAAGCAXXXXXXXXXXXX

Reverse Primer:

TTGAAGACAACTCGCGAAXXXXXXXXXXXX

TT- Prefix
GAAGAC- BbsI recognition
AA- Suffix (BbsI cleavage pattern)
CTCA- 4 bp overhang complementary to pAGM9121
AGCA- 4 bp overhang defining module 2 position (gene part) in *Yeast Secret and Detect* system
XXXX-Binding sequence of the protein-coding template (target protein)

Supplementary Fig. 1 Principle of modular UPO shuffling system

Design principle of the modular tripartite Golden Gate secretion system consisting of signal peptide, gene and C-terminal protein tag

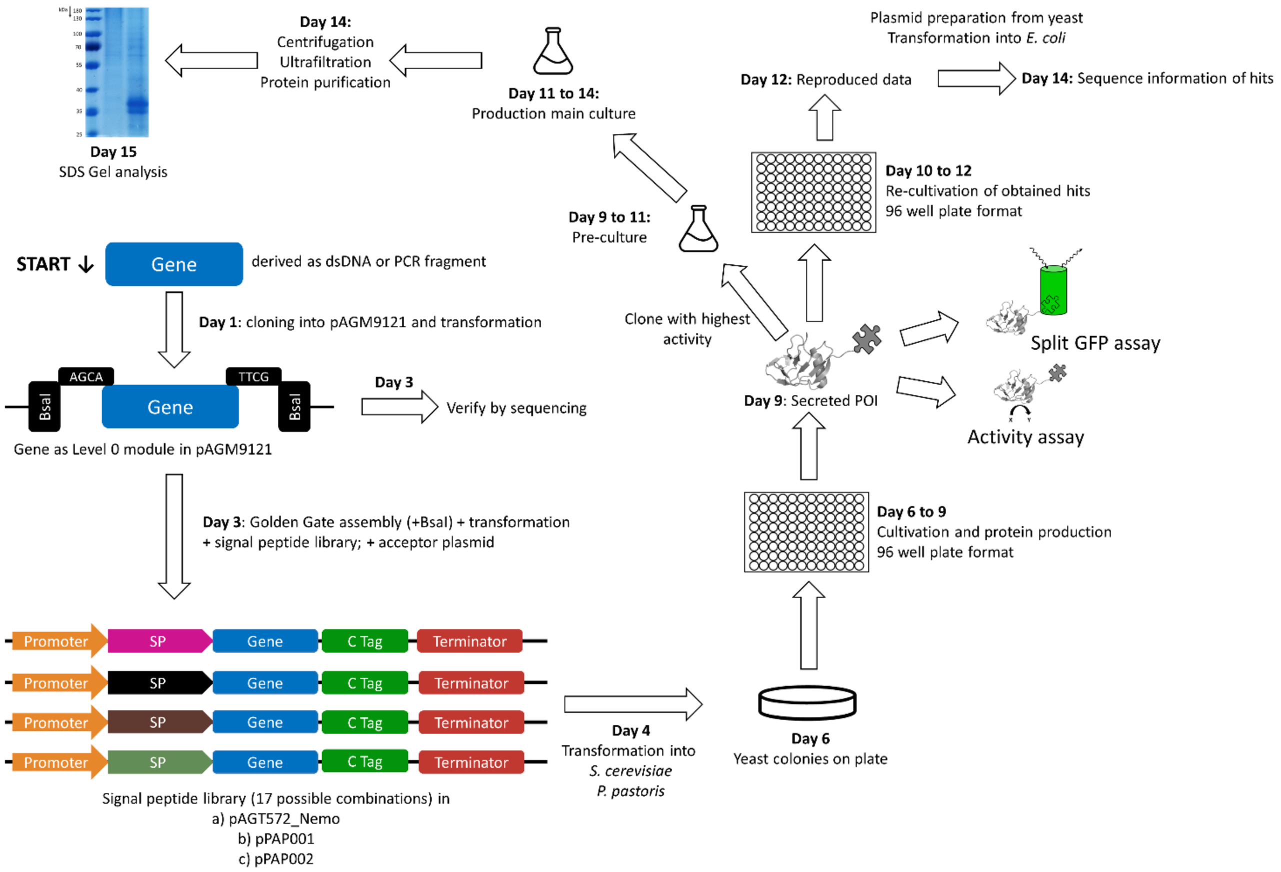

Supplementary Fig. 2 Schematic overview of the developed workflow with indication of minimal time requirements for the respective steps

Workflow of the Yeast Secrete and detect system in *Saccharomyces cerevisiae* with indication of minimal time requirements for the respective steps.

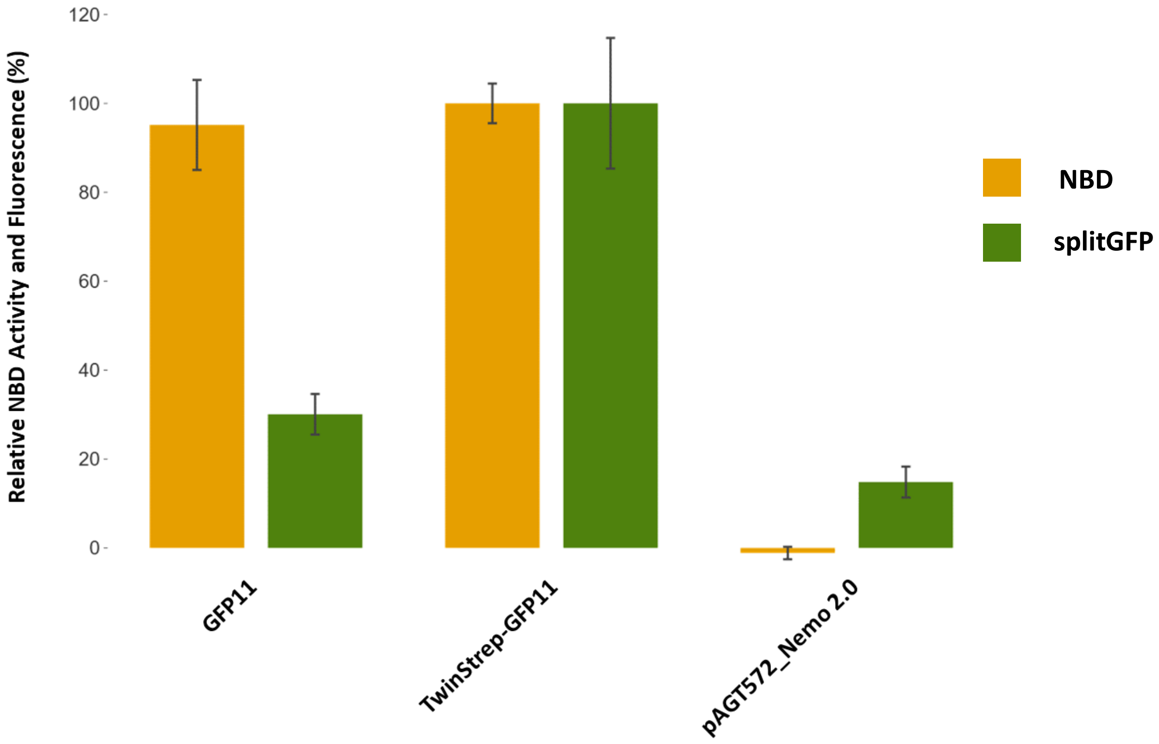

Supplementary Fig. 3 Comparison of C-terminal GFP11 and TwinStrep-GFP11 constructs

27 biological replicates of each construct (pAGT572_Nemo 2.0- *Gma*-UPO *Aae*UPO*) were screened for UPO activity by means of the NBD assay and split GFP assay using 20 µL culture supernatants from 96 well cultivations.

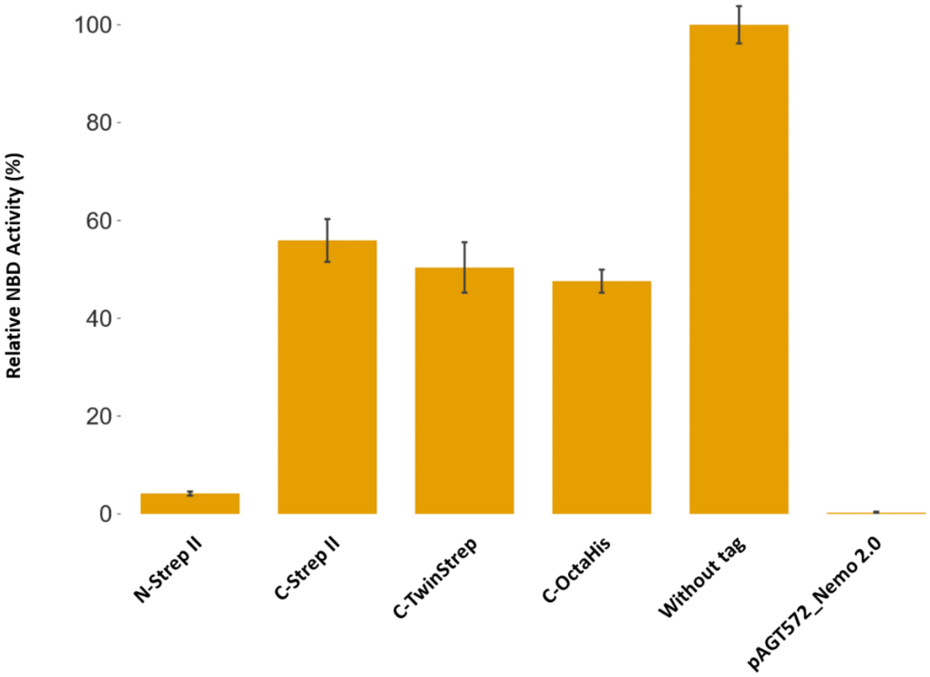

Supplementary Fig. 4 Comparison of differently tagged and untagged *Gma*-UPO-*Aae*UPO* constructs

8 biological replicates of each construct (pAGT572_Nemo 2.0- *Gma*-UPO *Aae*UPO*) were screened by means of the NBD assay using 20 µL culture supernatants from 96 well cultivations. N: N-terminal position; C: C-terminal position; mean ± standard deviation depicted

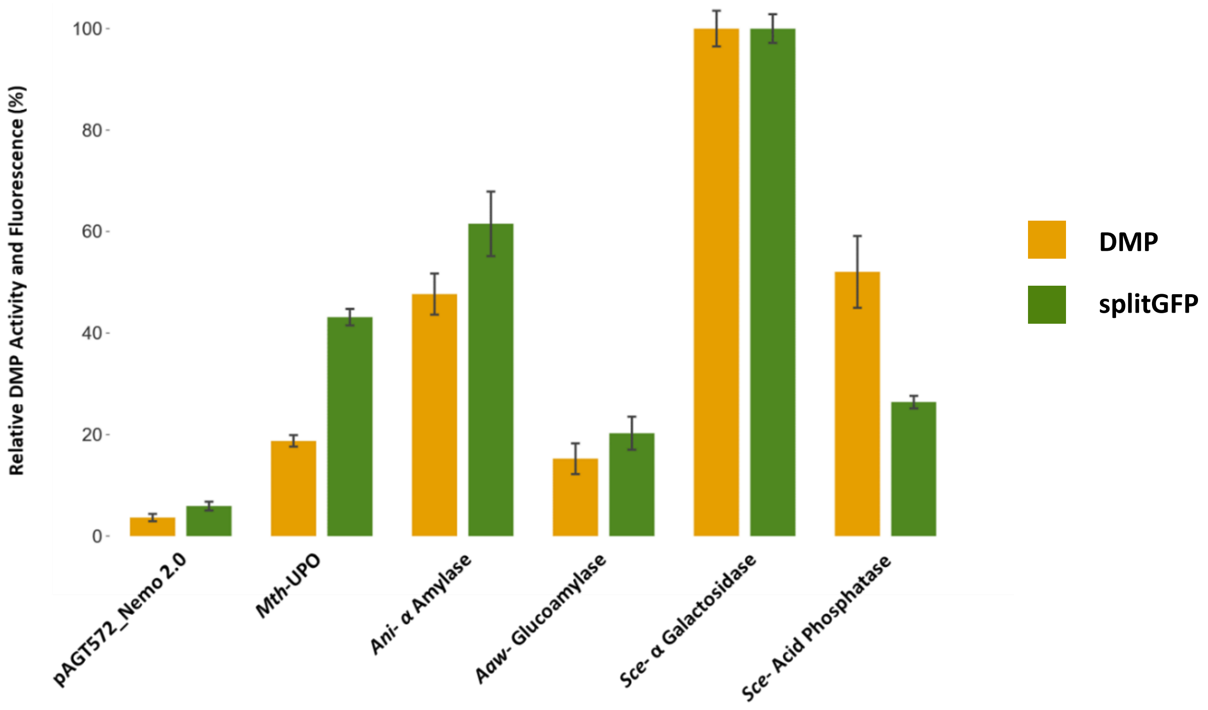

Supplementary Fig. 5 Identification of suitable signal peptides for *Mth*UPO secretion in *S. cerevisiae*

5 biological replicates of each construct were screened by means of the DMP and split GFP assay using 20 µL culture supernatants from 96 well cultivations; mean ± standard deviation depicted

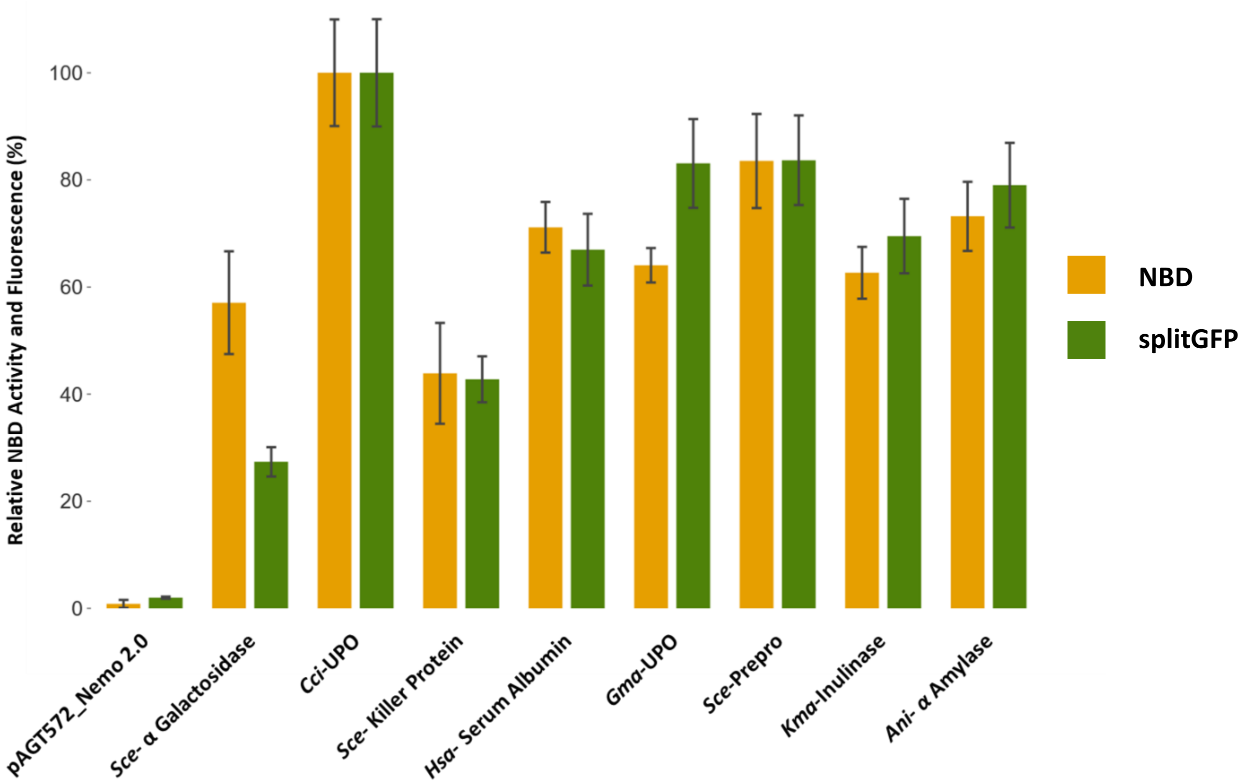

Supplementary Fig. 6 Identification of suitable signal peptides for *Tte*UPO secretion in *S. cerevisiae*

8 biological replicates of each construct were screened by means of the NBD and split GFP assay using 20 µL culture supernatants from 96 well cultivations; mean ± standard deviation depicted

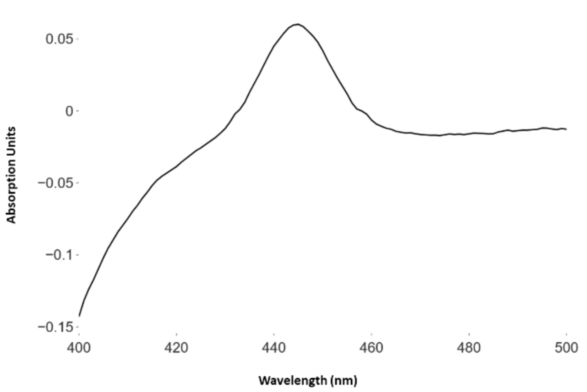

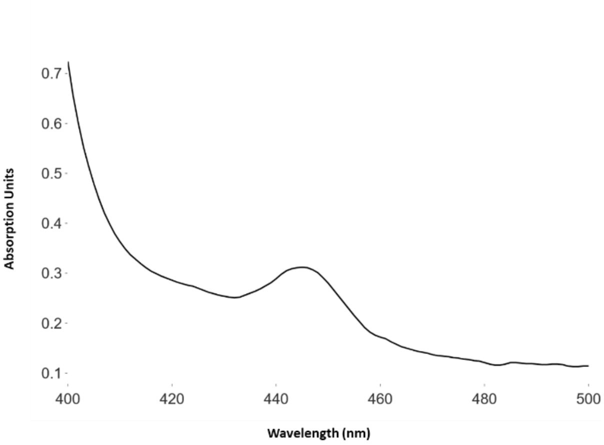

***Mro*UPO**
blanked with reduced elution sample (+$Na_2S_2O_4$)

CglUPO
blanked with unreduced elution sample (-$Na_2S_2O_4$)

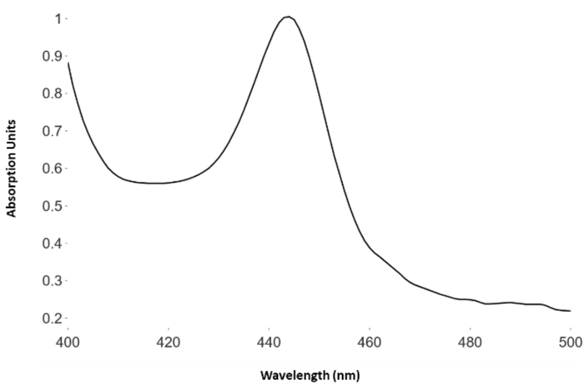

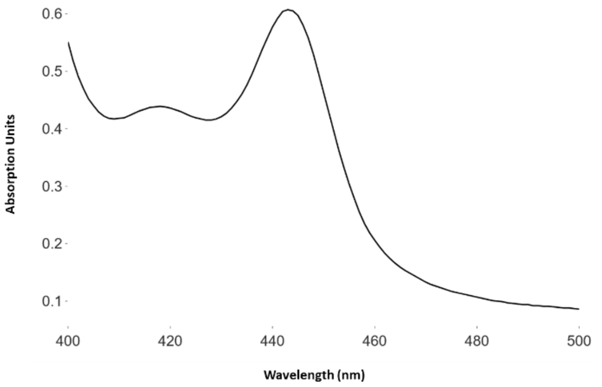

***Mth*UPO**
blanked with unreduced elution sample (-$Na_2S_2O_4$)

TteUPO
blanked with unreduced elution sample (-$Na_2S_2O_4$)

Supplementary Fig. 7 CO absorption measurements

Carbon monoxide differential spectra of UPOs produced in *Saccharomyces cerevisiae*, recorded in 100 mM potassium phosphate pH 7.0

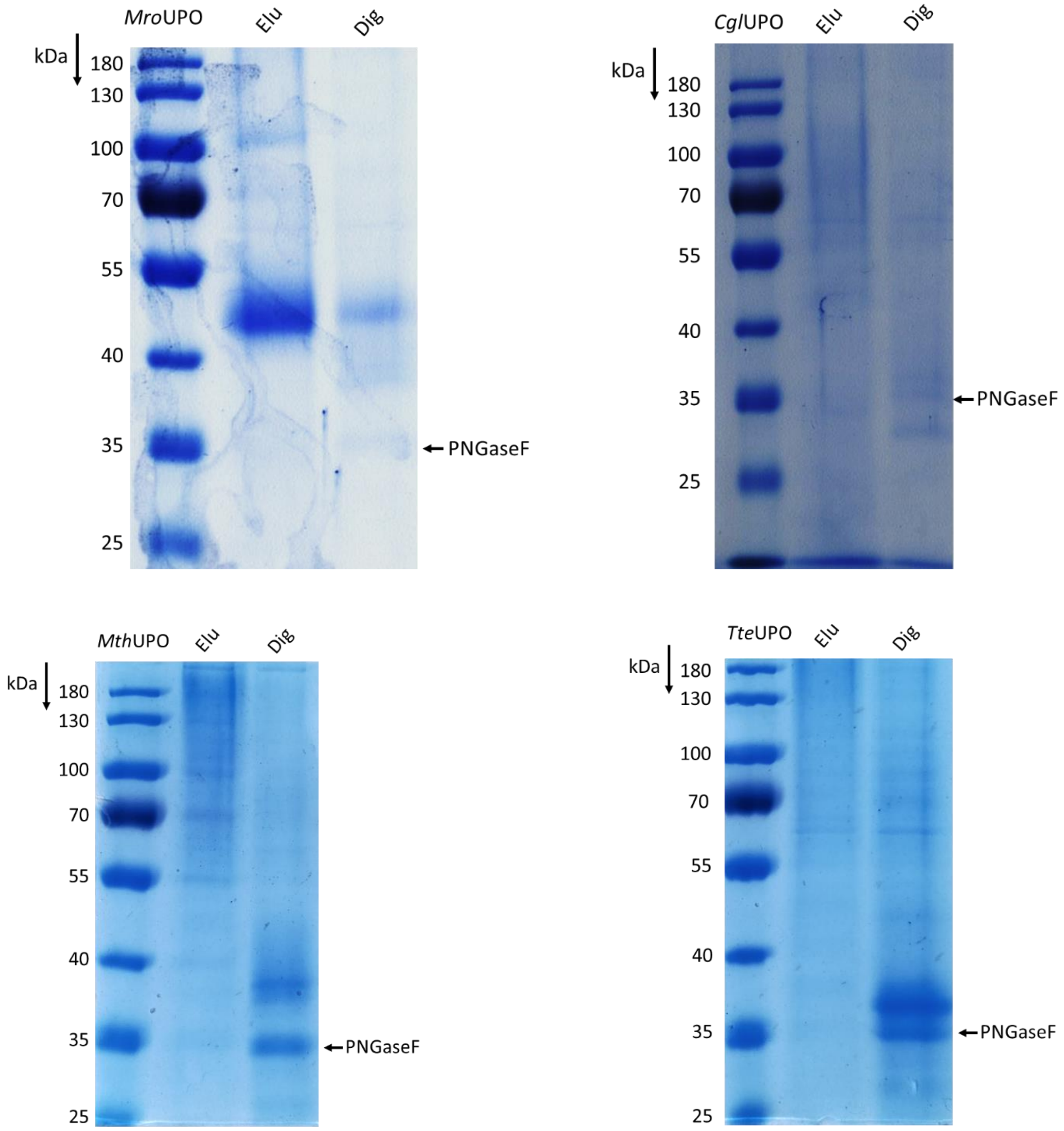
MroUPO
Elu
Dig
kDa
180
130
100
70
55
40
35
25
PNGaseF
CglUPO
Elu
Dig
kDa
180
130
100
70
55
40
35
25
PNGaseF
MthUPO
Elu
Dig
kDa
180
130
100
70
55
40
35
25
PNGaseF
TteUPO
Elu
Dig
kDa
180
130
100
70
55
40
35
25
PNGaseF

*Cgl*UPO partially PNGaseF digested (occurring, different protein species)

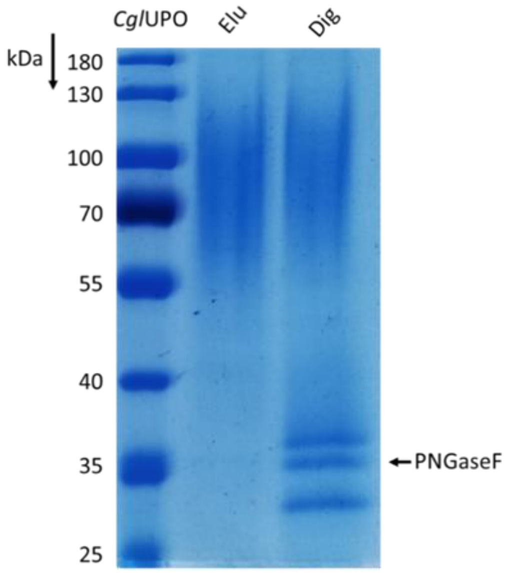

Supplementary Fig. 8 SDS PAGE analysis of UPOs produced in *S. cerevisiae*

SDS PAGE analysis of recombinant UPOs produced in *S. cerevisiae* utilising the elution fraction after dialysis (Elu) and PNGaseF digested, deglycosylated UPO sample (Dig).

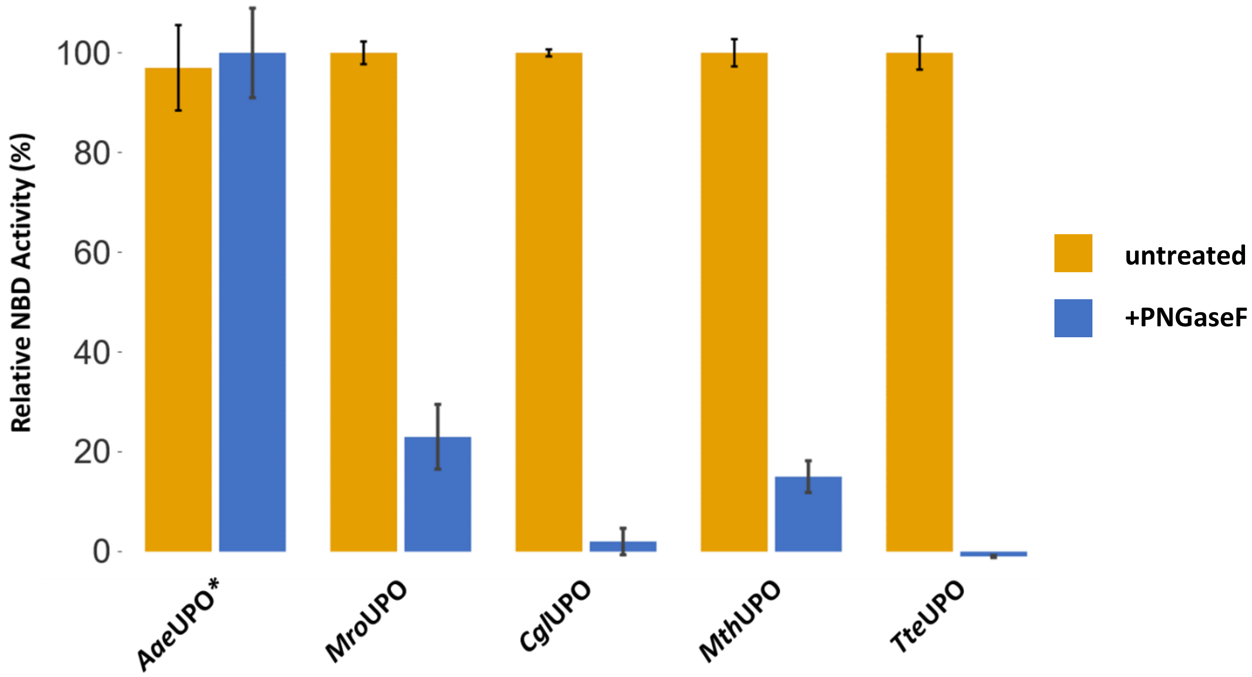

Supplementary Fig. 9 Enzymatic activity of UPOs derived from *S. cerevisiae* after deglycosylation

A sample of 90 µL of purified enzyme was subjected to native deglycosylation by PNGaseF (24 h: *Aae*UPO*, *Mth*UPO, *Tte*UPO and 48 h: *Mro*UPO, *Cgl*UPO). 3 technical replicates (20 µL) were measured for UPO activity by means of the NBD assay; mean ± standard deviation depicted

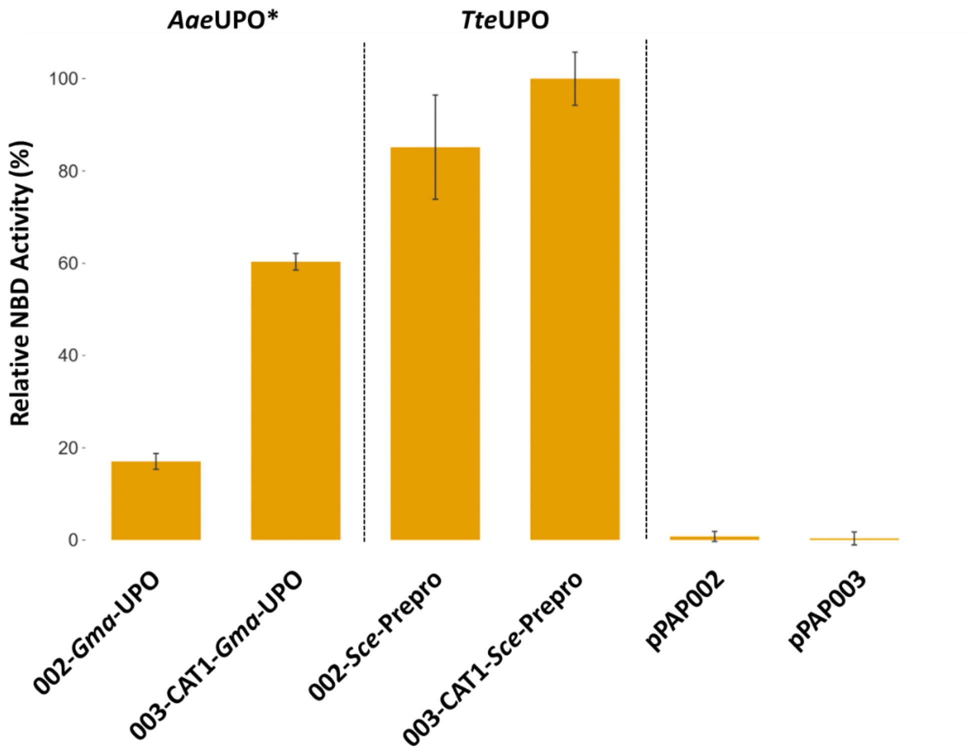

Supplementary Fig. 10 Comparison of UPO production from episomal and integrative constructs

12 biological replicates of each *Pichia pastoris* construct were screened for UPO activity by means of the NBD assay using 20 µL culture supernatants from 96 well cultivations; mean ± standard deviation depicted

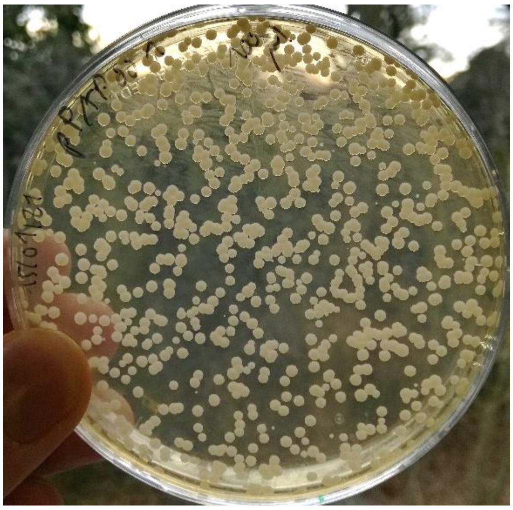

episomal expression plasmid (pPAP001)

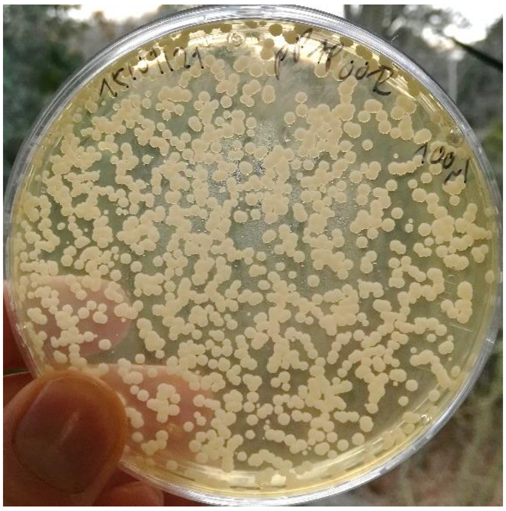

episomal expression plasmid (pPAP002)

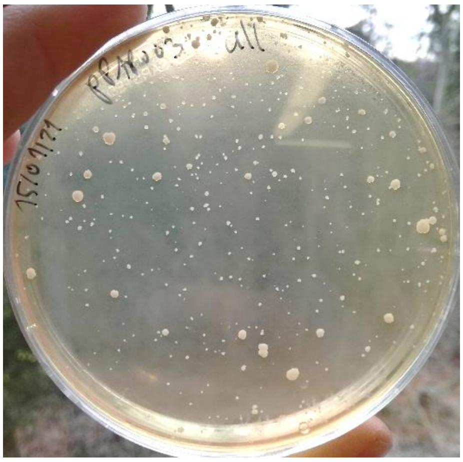

integrative acceptor plasmid (pPAP003)

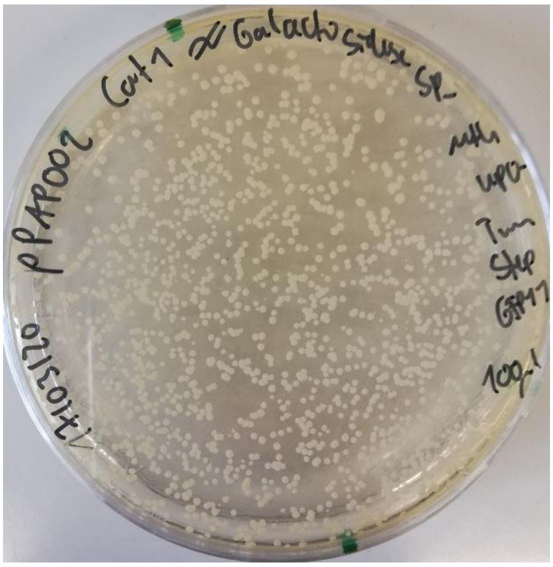

episomal *Mth*UPO construct (pPAP002)

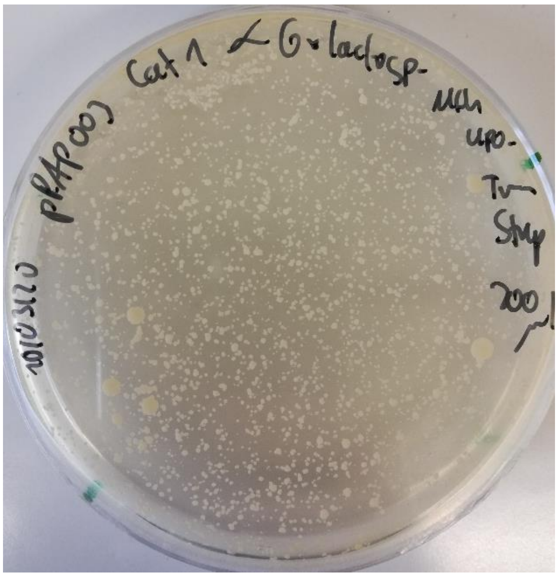

integrative *Mth*UPO construct (pPAP003)

Supplementary Fig. 11 Comparison colony shape and size episomal and integrative constructs

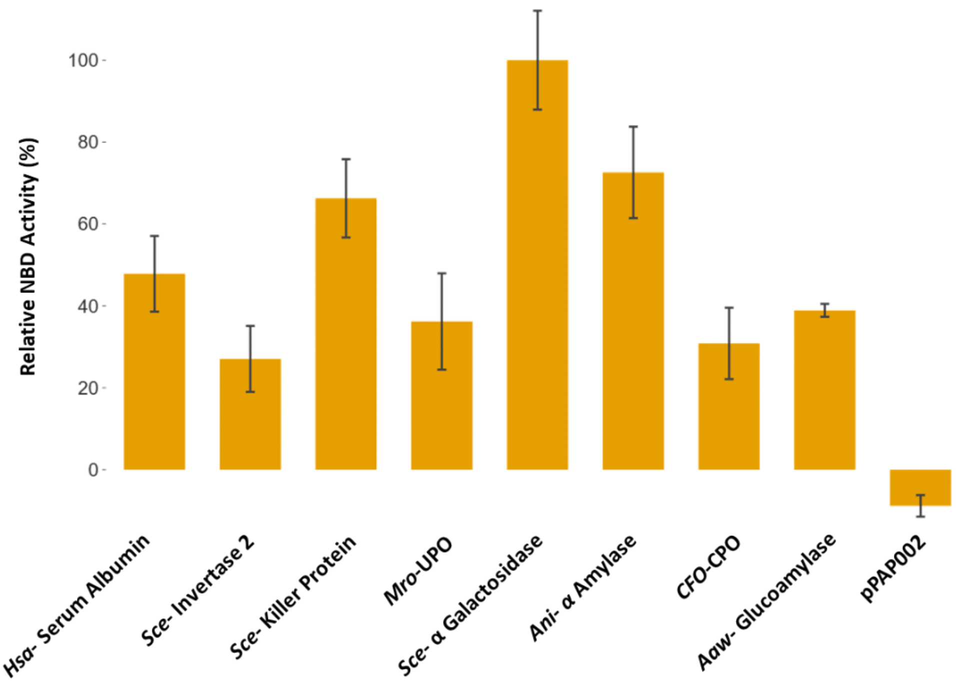

Supplementary Fig. 12 Identification of suitable signal peptides for *Mth*UPO secretion in *P. pastoris*

6 biological replicates of each construct were screened for UPO activity by means of the NBD assay using 20 µL culture supernatants from 96 well cultivations; mean ± standard deviation depicted

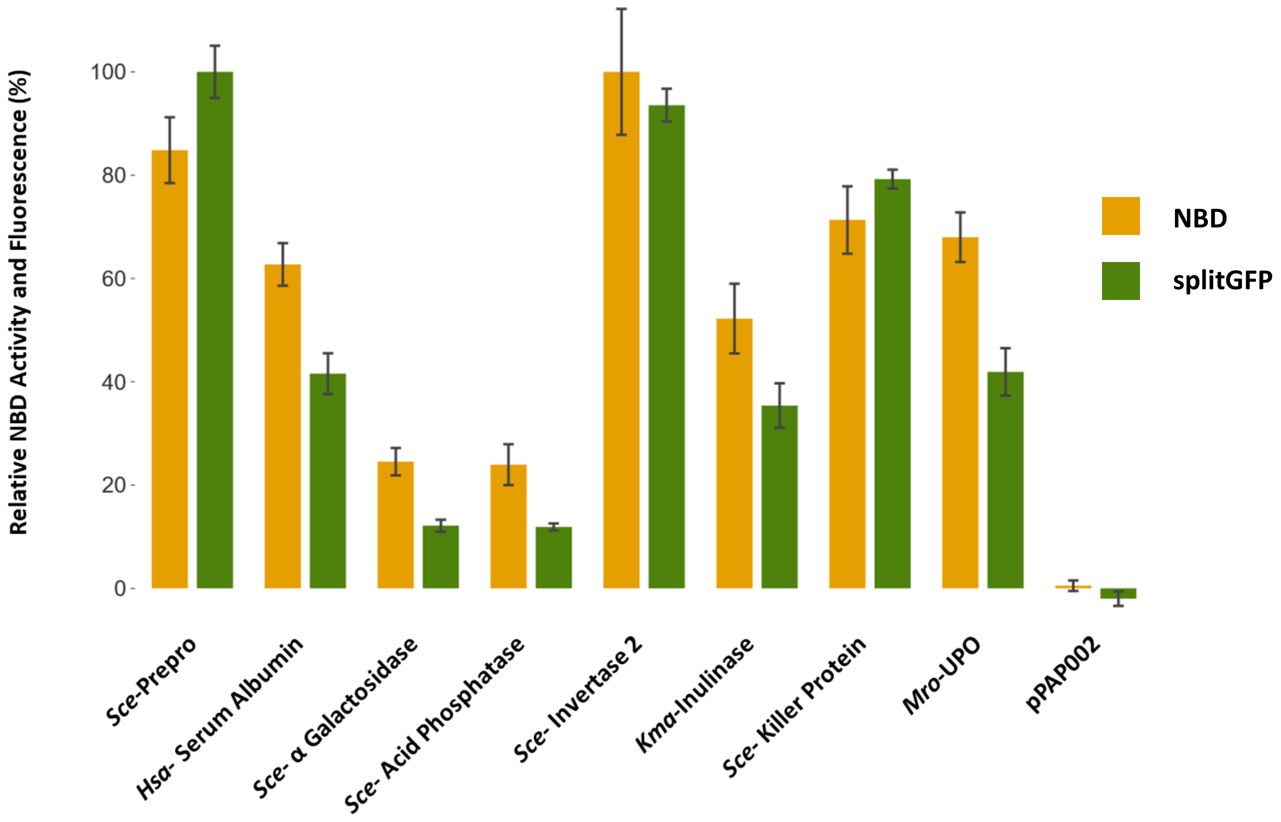

Supplementary Fig. 13 Identification of suitable signal peptides for *Tte*UPO secretion in *P. pastoris*

6 biological replicates of each construct were screened by means of the NBD and split GFP assay using 20 µL culture supernatants from 96 well cultivations; mean ± standard deviation depicted

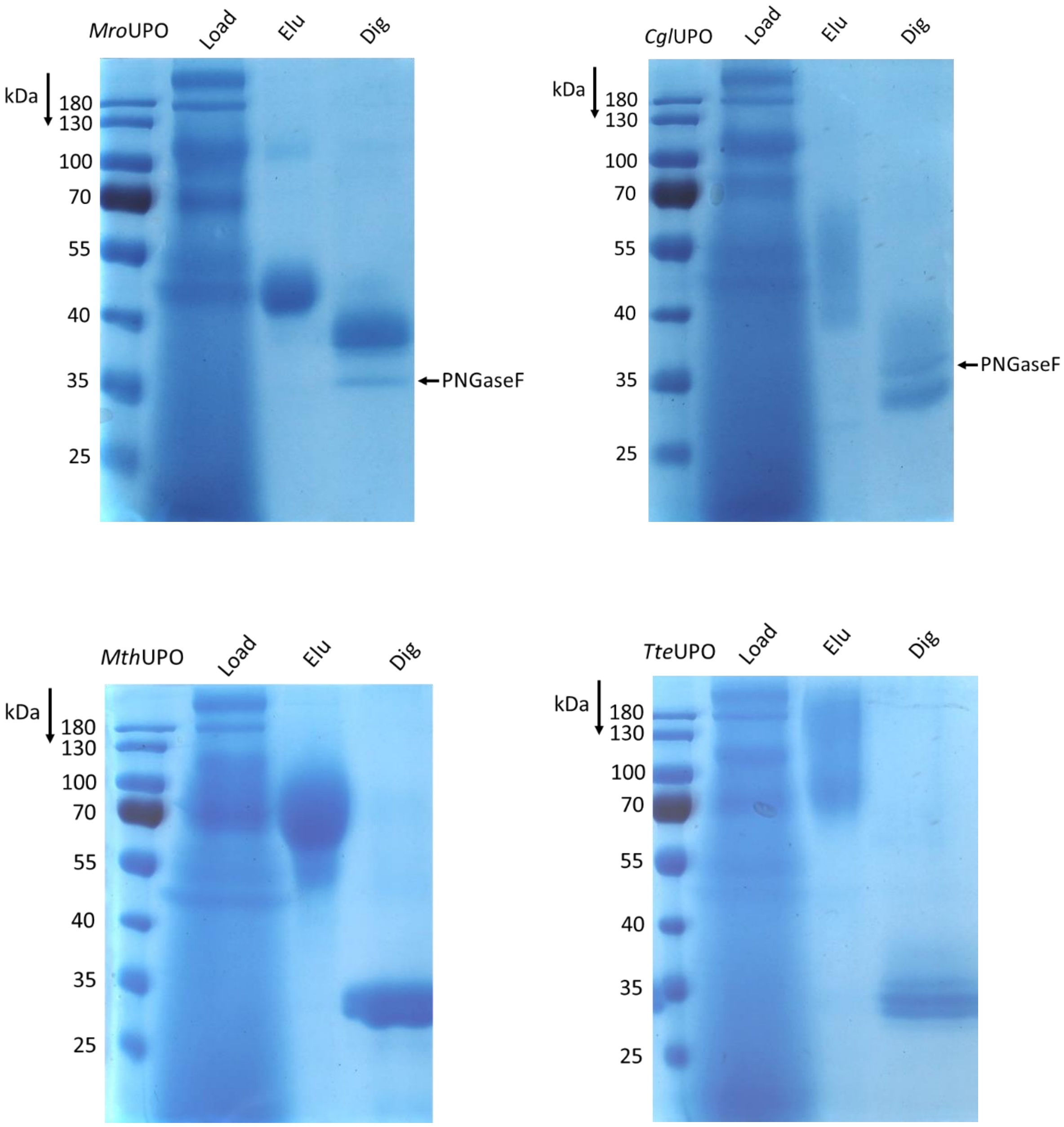

Supplementary Fig. 14 SDS PAGE analysis of UPOs derived from *P. pastoris*

SDS PAGE analysis of recombinant UPOs produced in *P. pastoris* utilising the concentrated supernatant (Load) the elution fraction after dialysis (Elu) and PNGaseF digested, deglycosylated UPO sample (Dig).

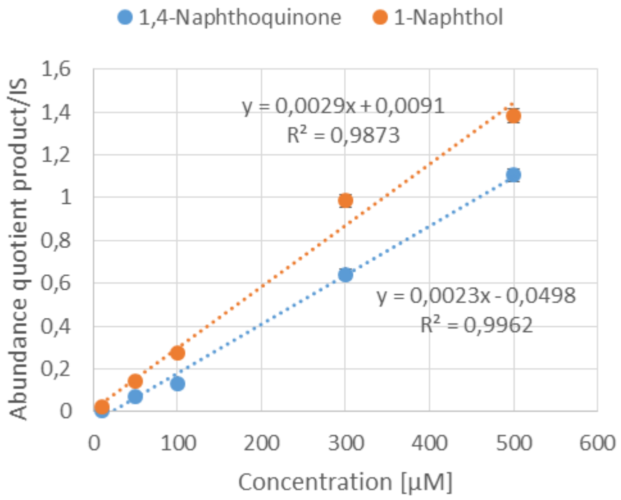

1,4-Naphthoquinone
1-Naphthol
y = 0,0029x + 0,0091
R² = 0,9873
y = 0,0023x - 0,0498
R² = 0,9962
Abundance quotient product/IS
Concentration [µM]

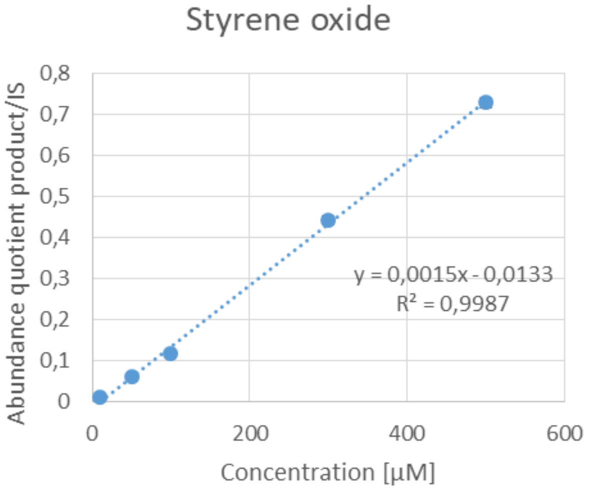

Styrene oxide
y = 0,0015x - 0,0133
R² = 0,9987
Abundance quotient product/IS
Concentration [µM]

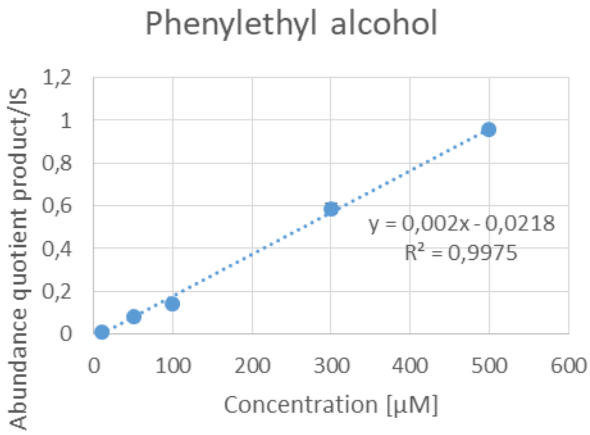

Phenylethyl alcohol
y = 0,002x - 0,0218
R² = 0,9975
Abundance quotient product/IS
Concentration [µM]

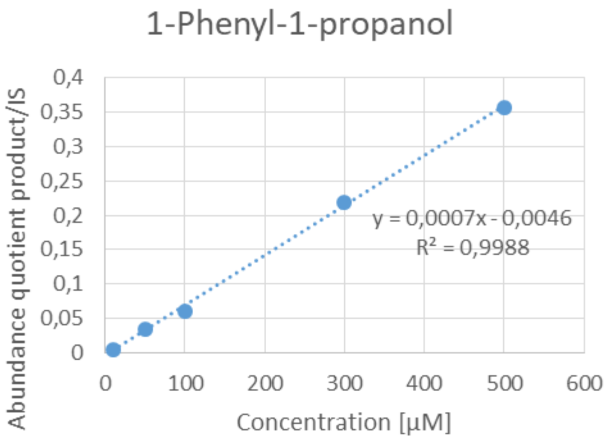

1-Phenyl-1-propanol
y = 0,0007x - 0,0046
R² = 0,9988
Abundance quotient product/IS
Concentration [µM]

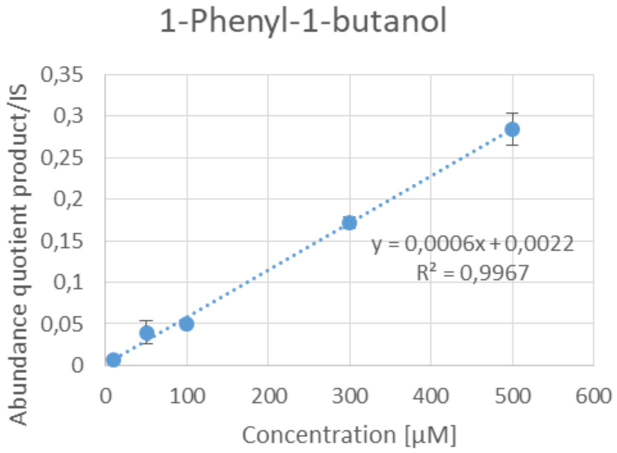

1-Phenyl-1-butanol
y = 0,0006x + 0,0022
R² = 0,9967
Abundance quotient product/IS
Concentration [µM]

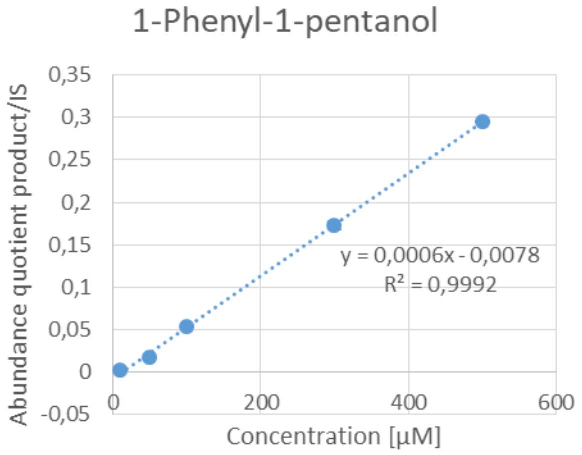

1-Phenyl-1-pentanol
y = 0,0006x - 0,0078
R² = 0,9992
Abundance quotient product/IS
Concentration [µM]

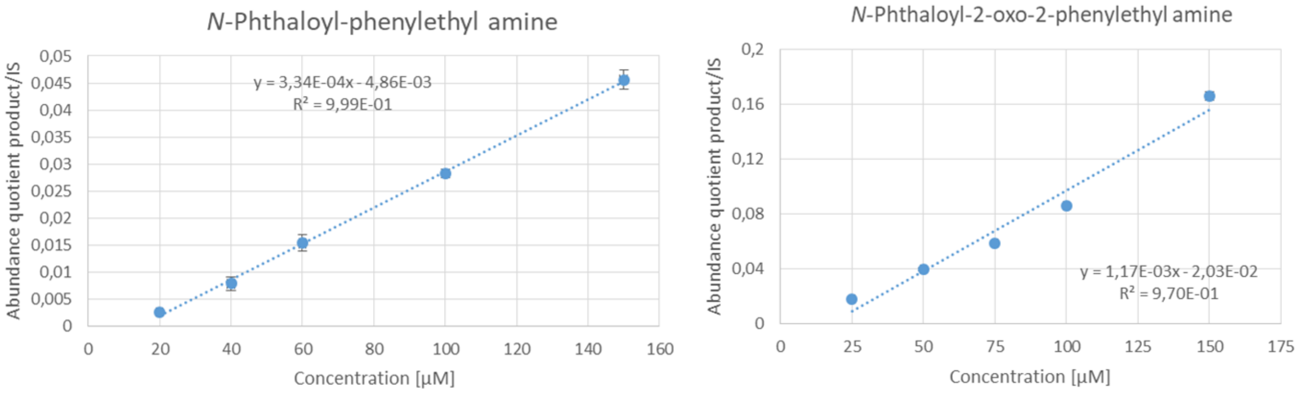

Supplementary Fig. 15 Calibration curves GC-MS

Calibration curves for GC MS based product quantification based on linear fitting of the obtained data points. Each sample was measured as a triplicate and correlated to an internal standard (reference substance)

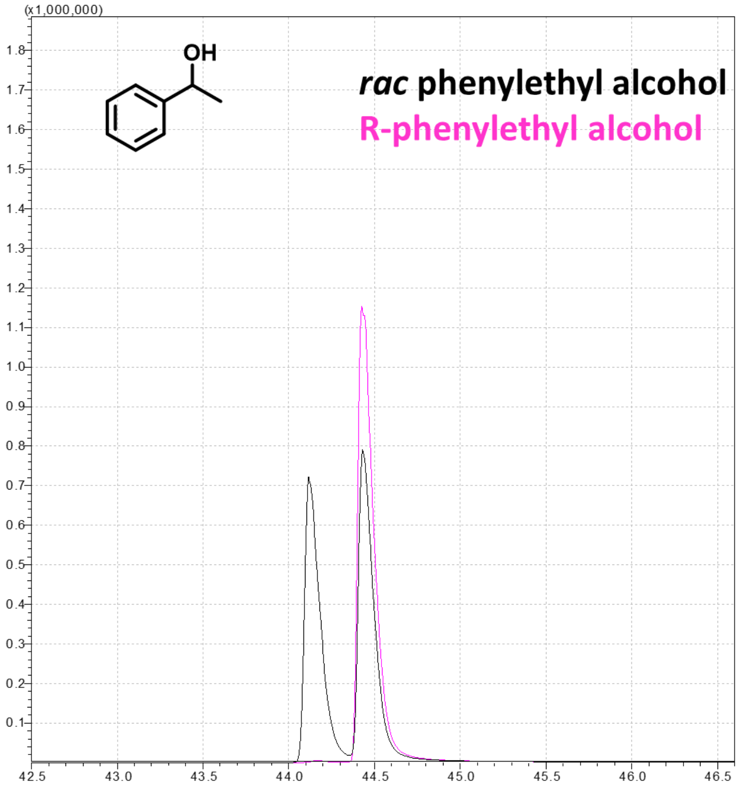

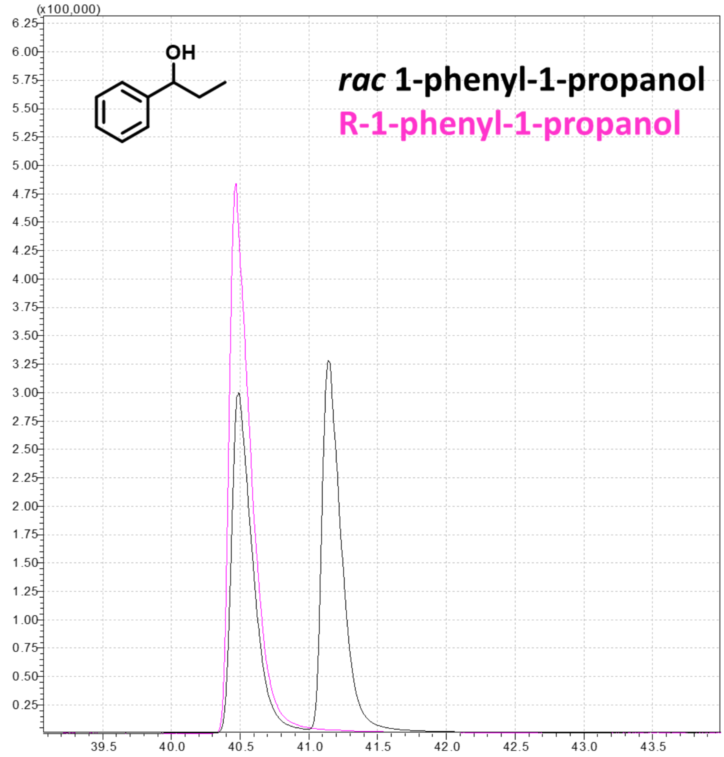

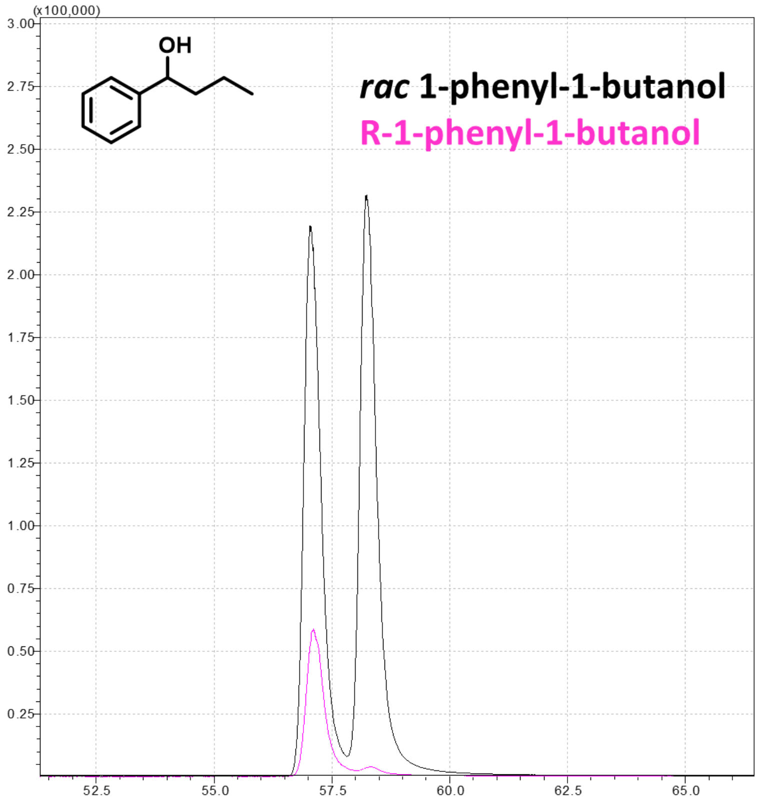

Supplementary Fig. 16 Enantiomeric separation 1-phenylethyl alcohol products

Phenylethane

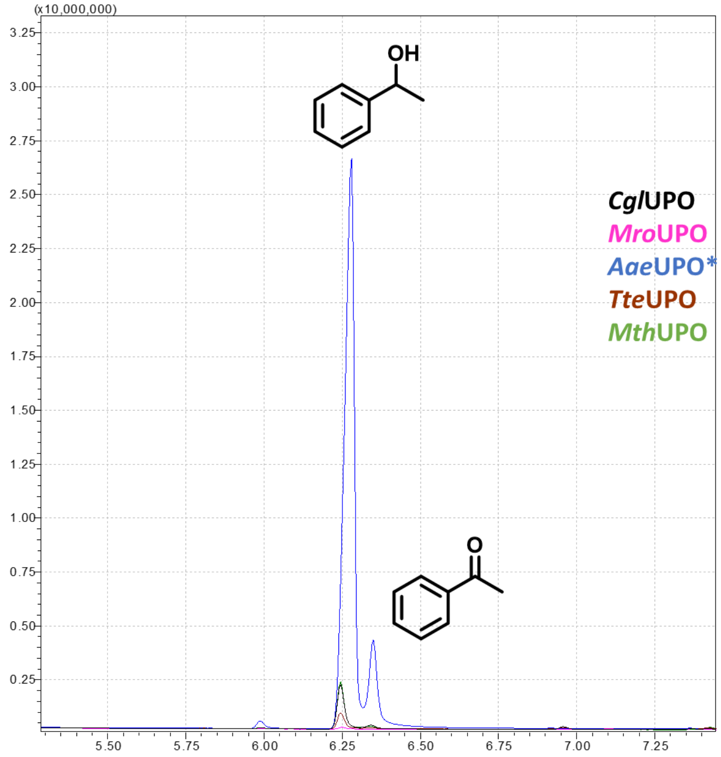

Phenylpropane

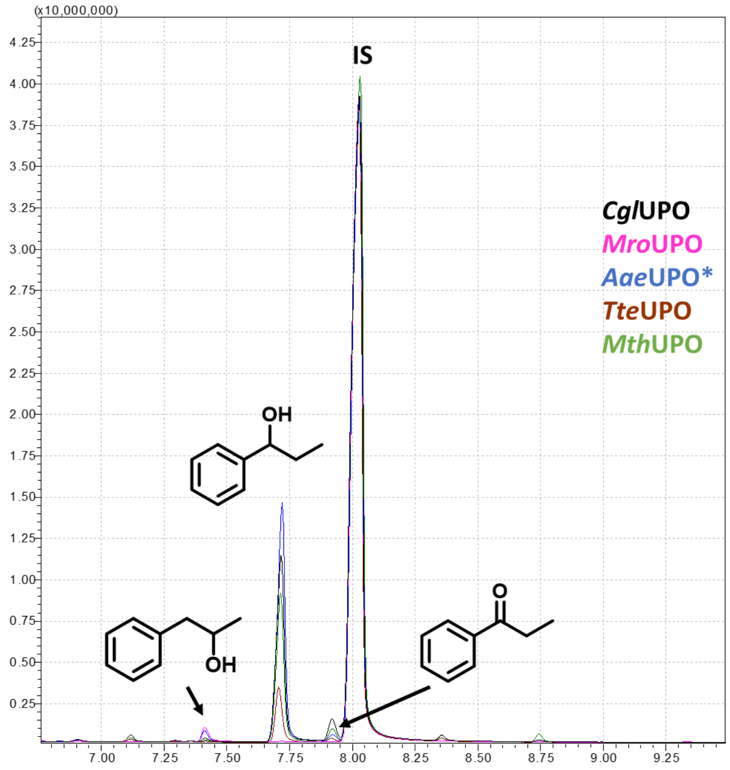

Phenylbutane

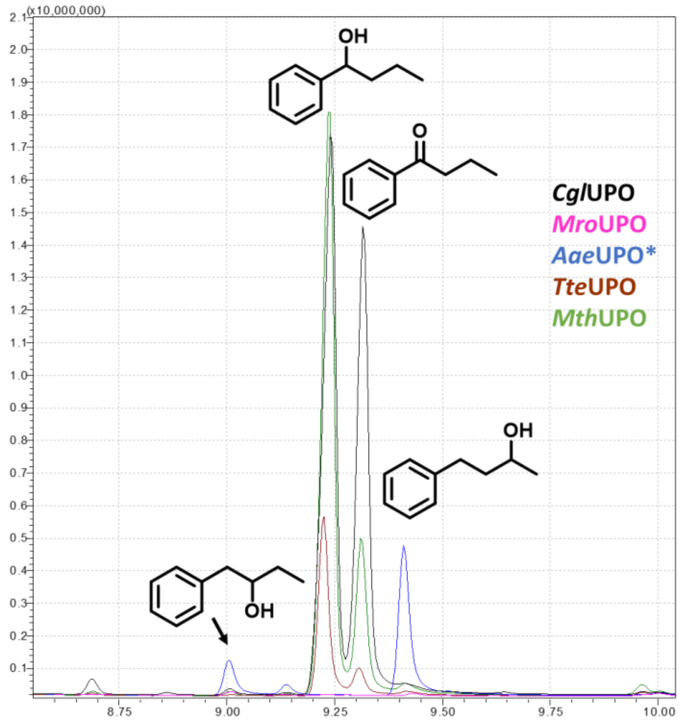

Phenylpentane

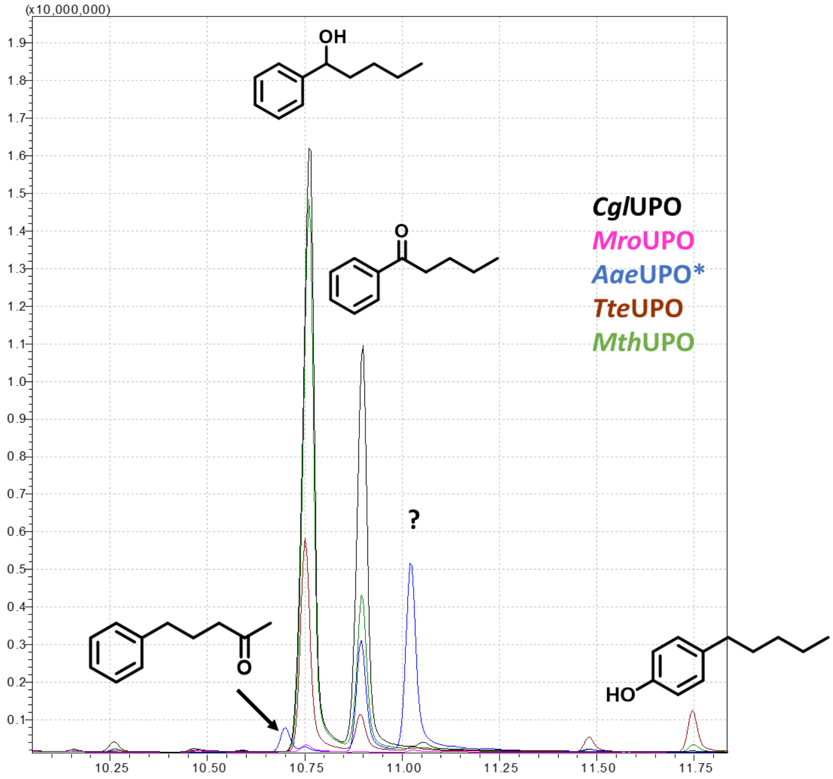

Supplementary Fig. 17 Scan Mode measurements of UPO catalysed Phenyl alkane hydroxylation

Exemplary GC MS measurements of various phenyl alkane substrate conversion catalysed by five distinct UPOs and occurring product mixtures obtained in the scan mode.

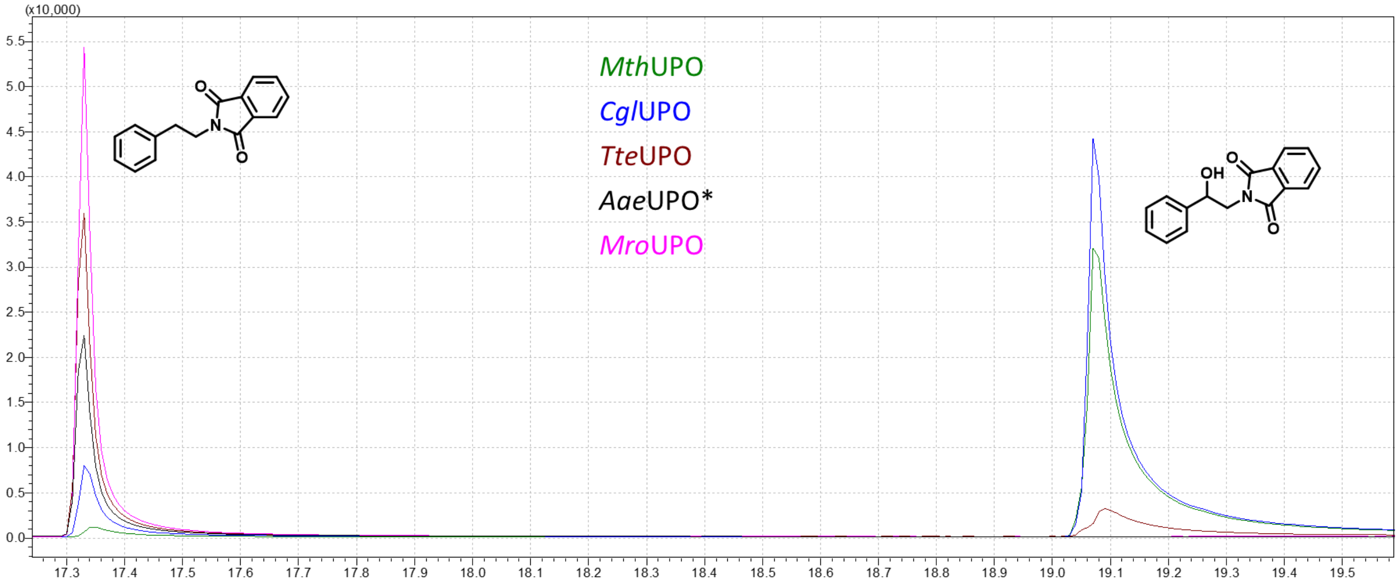

Supplementary Fig. 18 SIM Mode measurements of UPO catalysed *N*-Phthaloyl-phenylethyl amine conversion

Exemplary GC MS measurements of *N*-Phthaloyl-phenylethyl amine conversion catalysed by five distinct UPOs and occurring product mixtures obtained in the SIM mode.

*Mth*UPO

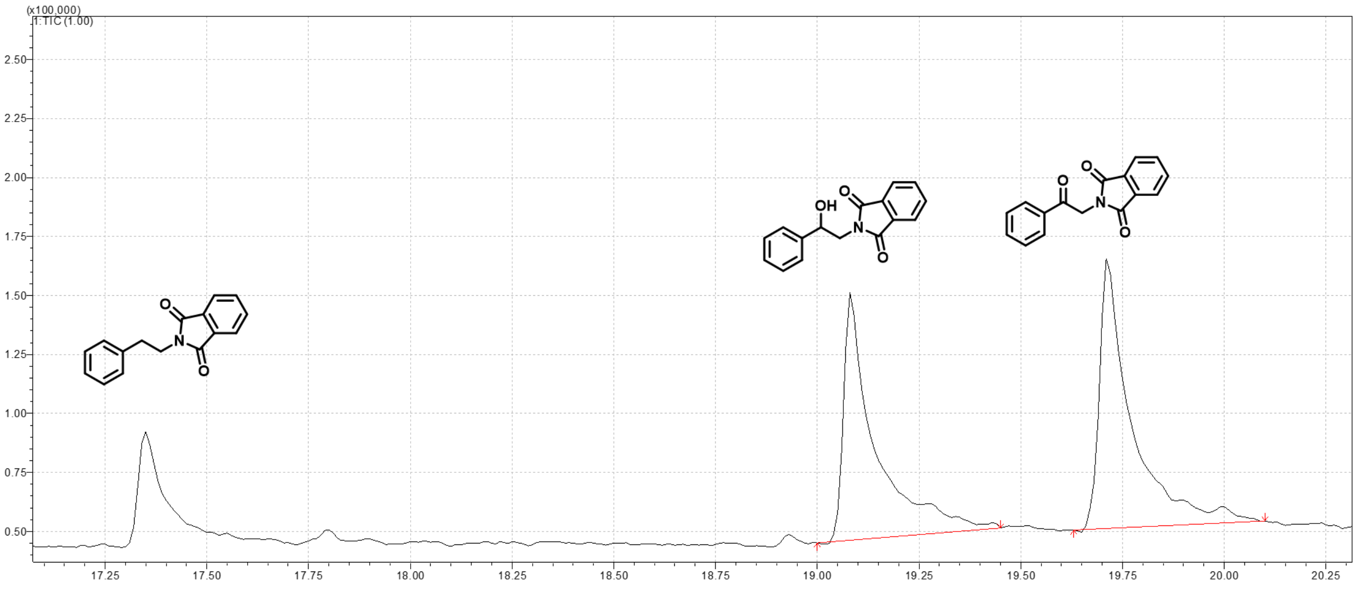

***Cgl*UPO**

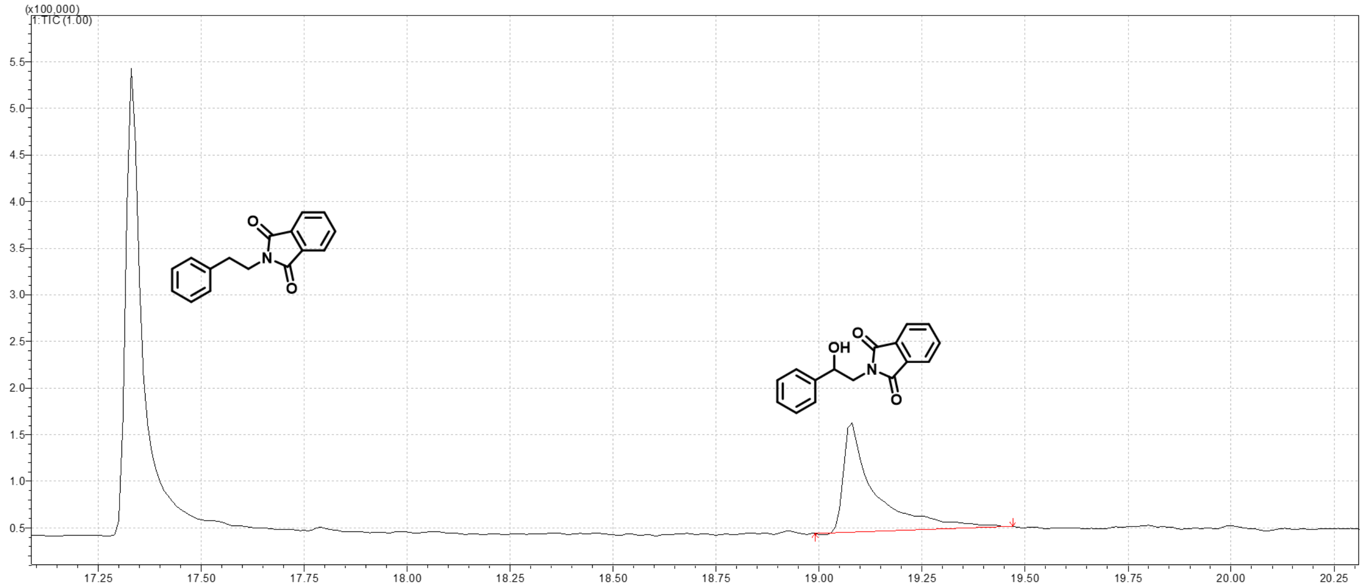

***Tte*UPO**

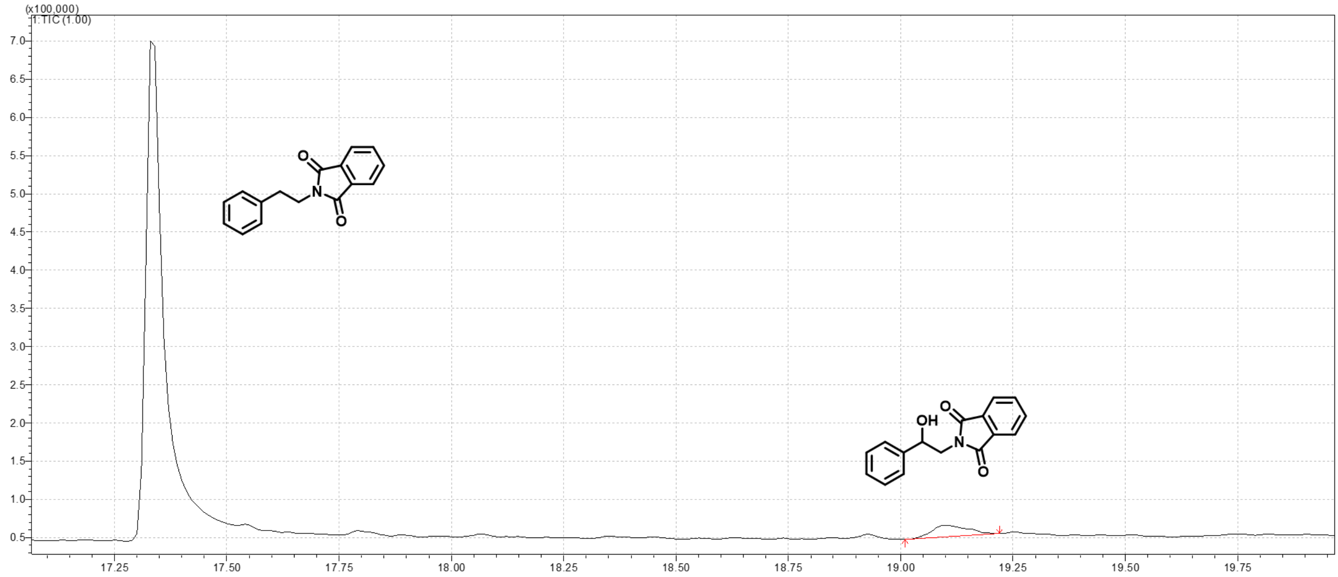

Supplementary Fig. 19 Scan Mode measurements of UPO catalysed *N*-Phthaloyl-phenylethyl amine conversion

Exemplary GC MS measurements of *N*-Phthaloyl-phenylethyl amine conversion catalysed by *Mth*UPO, *Cgl*UPO and T*te*UPO depicting formation of the alcohol product (all) and further oxidation to the ketone product (*Mth*UPO) obtained in Scan Mode.

Small scale reactions (400 µL) and utilised standards

(R,S)-2-N-Phthaloyl-1-phenylethanol

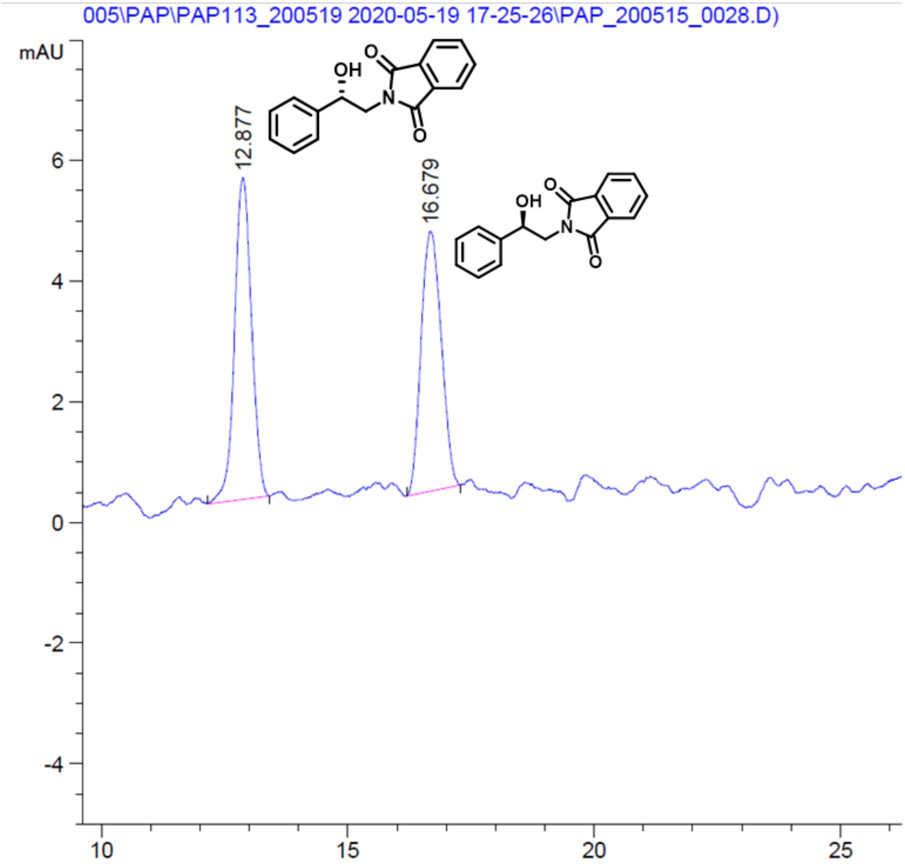

(S)-(+)-2-N-Phthaloyl-1-phenylethanol

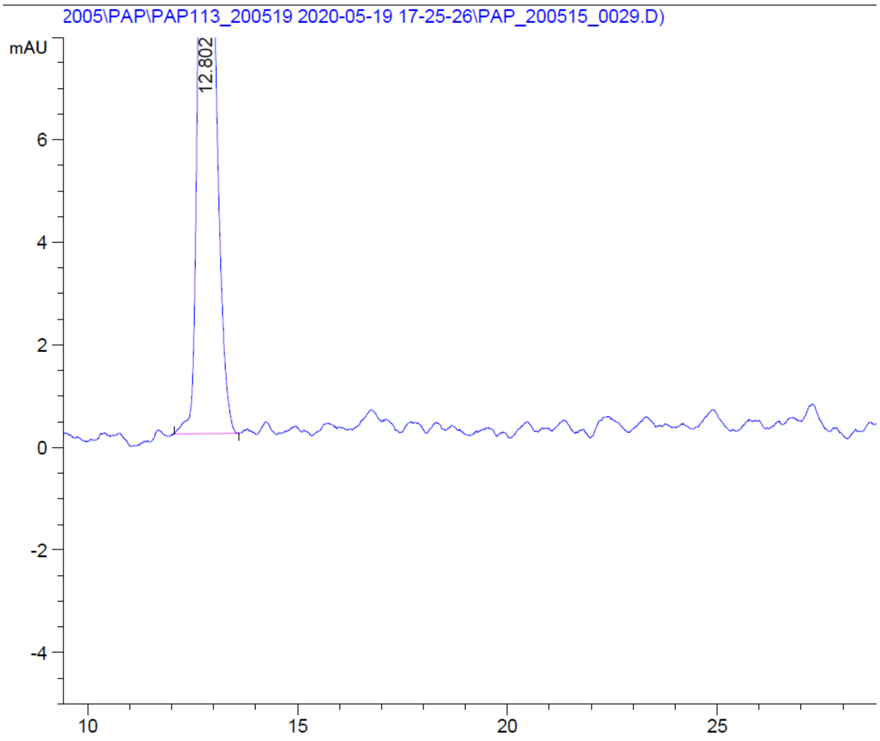

***Mth*UPO**

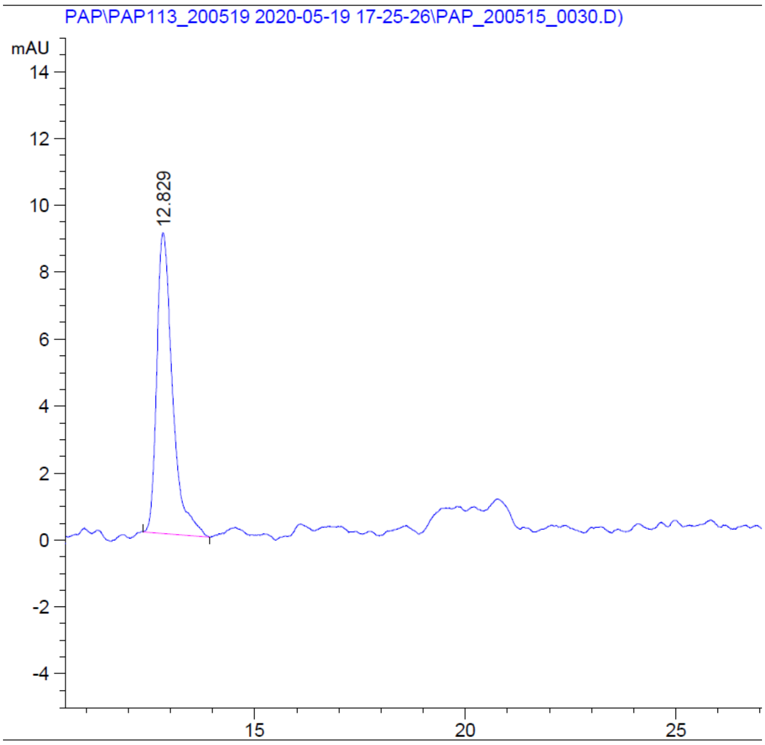

***Cgl*UPO**

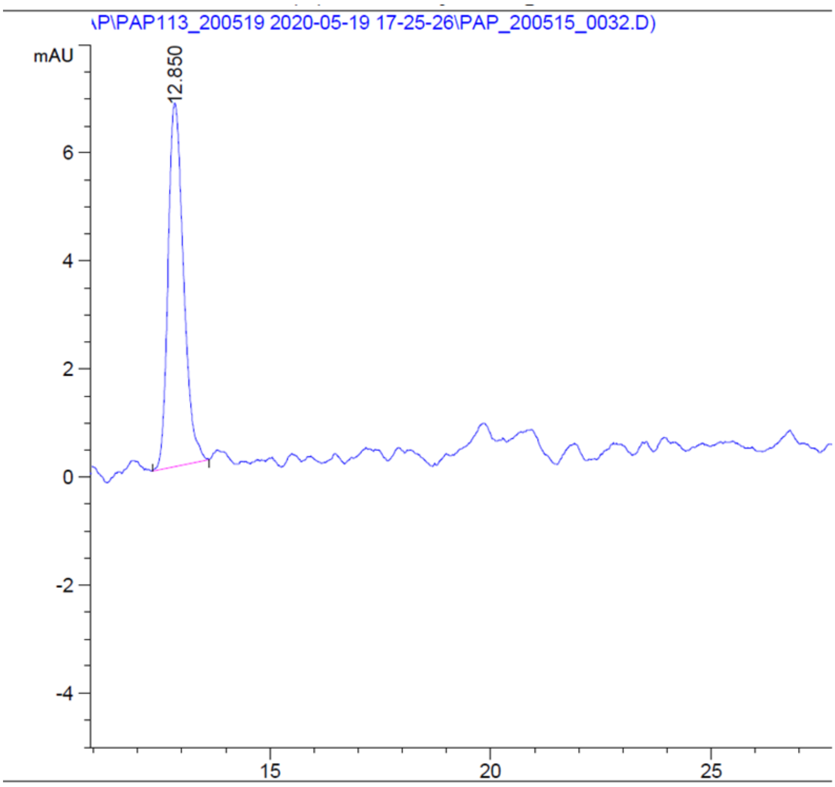

***Tte*UPO**

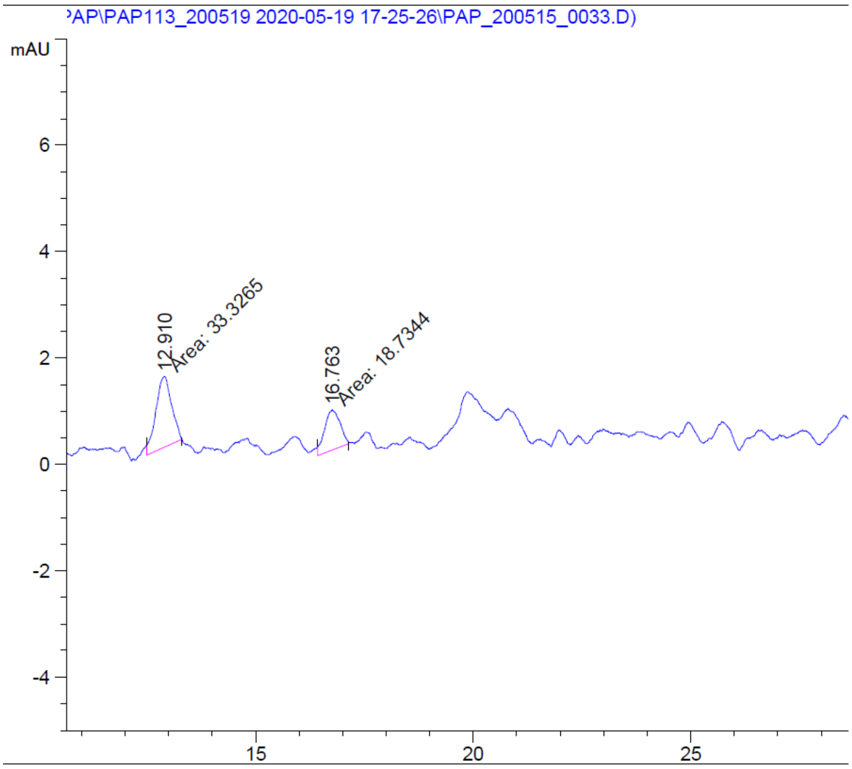

Larger scale reactions and standards for enantiomeric excess determination

***Mth*UPO**

(R,S)-2-N-Phthaloyl-1-phenylethanol

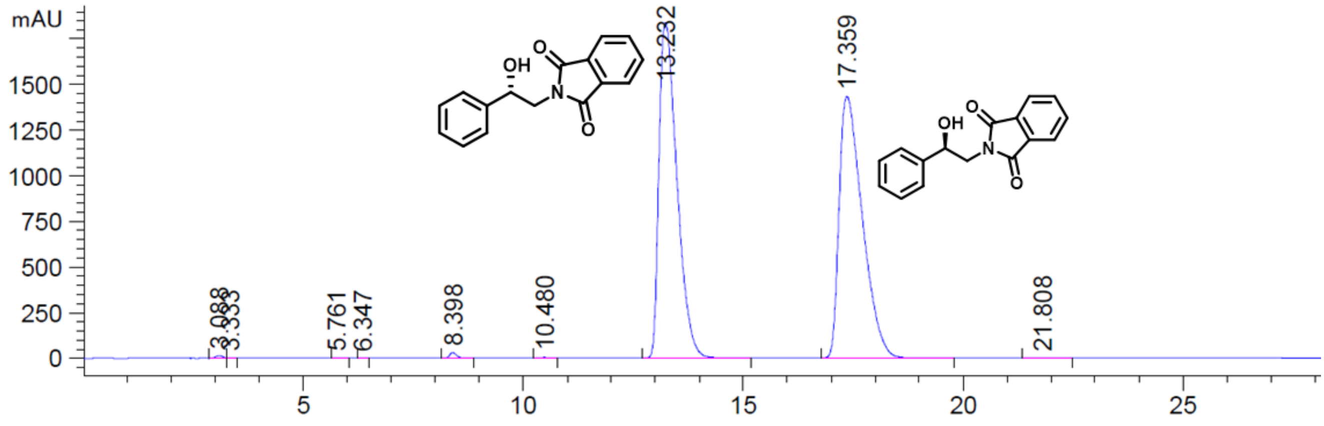

(S)-(+)-2-N-Phthaloyl-1-phenylethanol

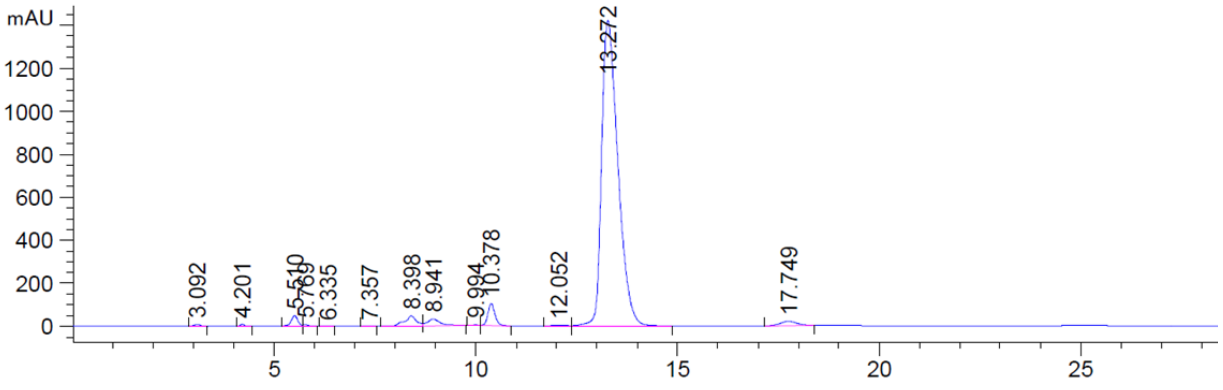

Preparative enzymatic conversion (300 mL approach)

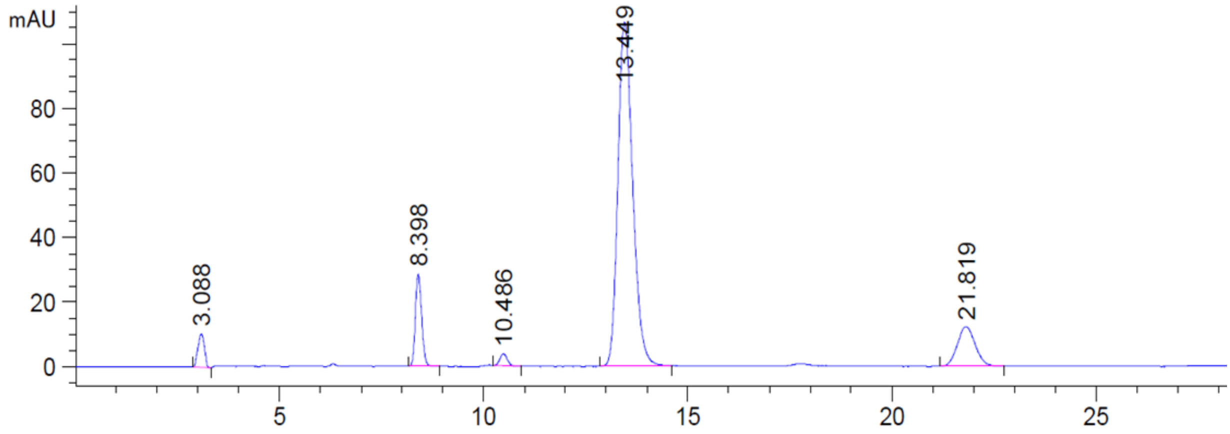

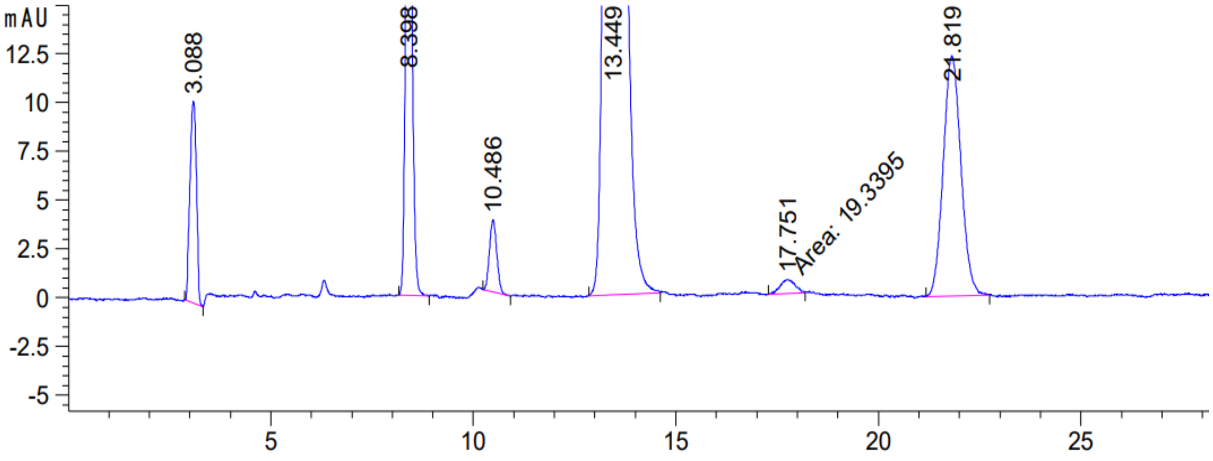

CglUPO

(R,S)-2-N-Phthaloyl-1-phenylethanol

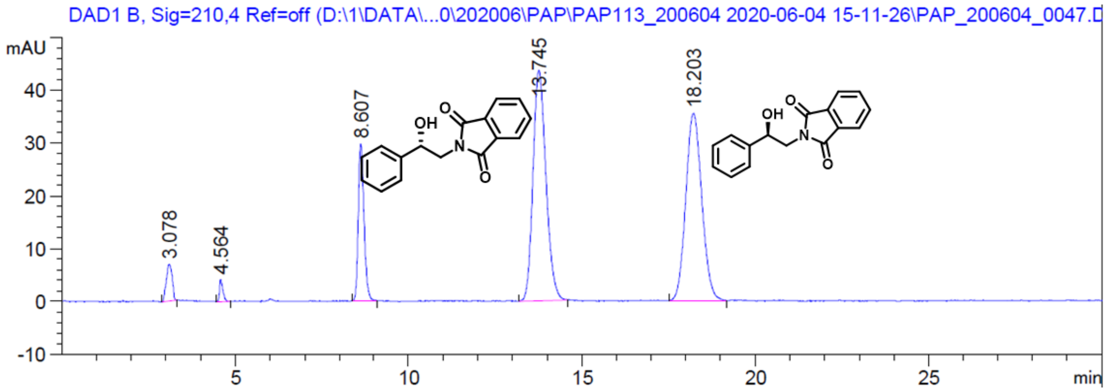

(S)-(+)-2-N-Phthaloyl-1-phenylethanol

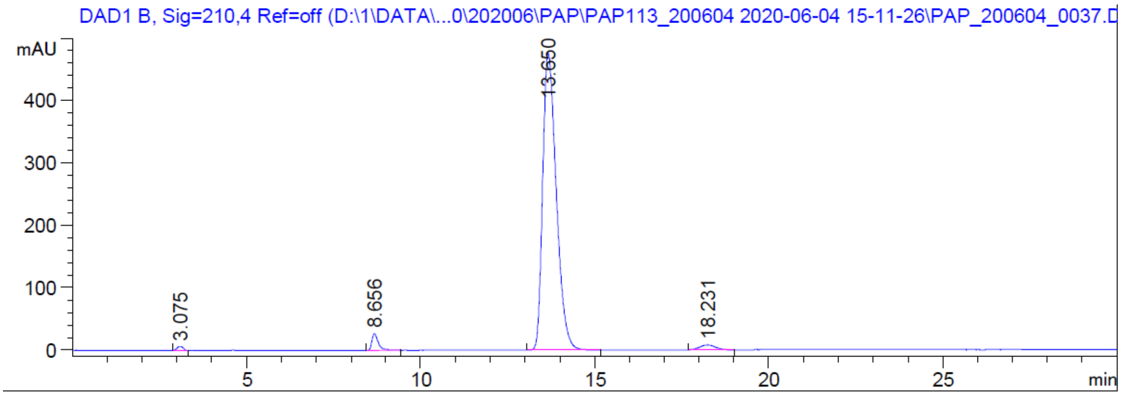

Large scale enzymatic conversion (10 ml reaction setup)

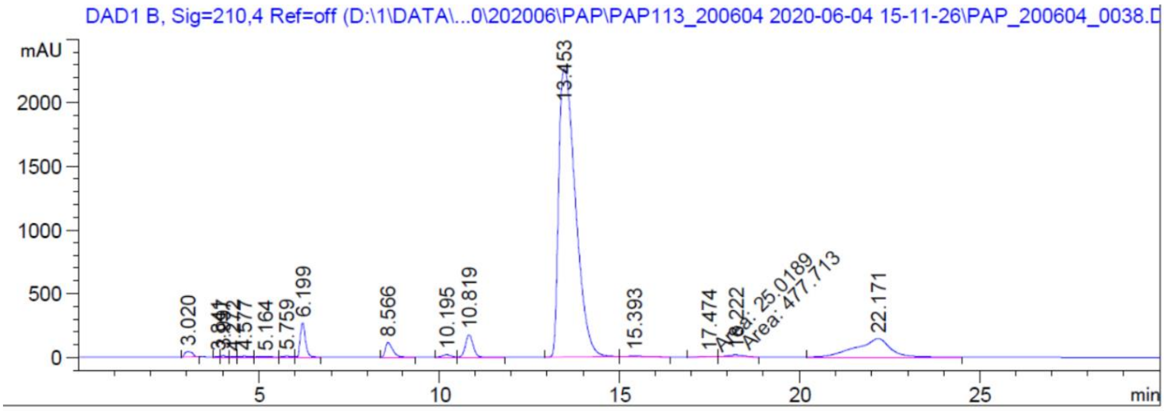

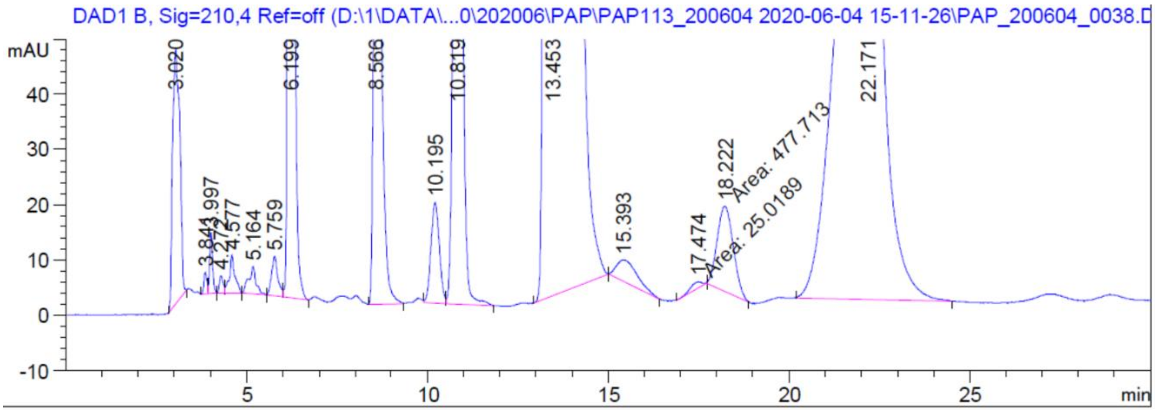

Supplementary Fig. 20 Chiral HPLC analysis of UPO catalysed *N*-Phthaloyl-phenylethyl amine conversion

Exemplary chiral HPLC measurements of *N*-Phthaloyl-phenylethyl amine conversion catalysed by *Mth*UPO, *Cgl*UPO and T*te*UPO on analytical scale (all enzymes) and after reaction upscaling (*Mth*UPO and *Cgl*UPO).

II. Supplementary Tables

Supplementary Table 1 Overview of oligonucleotides for sequencing of the created plasmids

Overview of oligonucleotides which can be employed for sequencing of the respective created Golden Gate plasmids

Name	*Sequence (5´ → 3´)*
pAGM9121_for	CCTGTCGGGTTTCGCCACCT
pAGM9121_rev	GCCGTTACCACCGCTGCGTT
pAGT572_Nemo 2.0_for	CATCTTATTAAAGTATCATCAAGAAATTGTTA
pAGT572_Nemo_rev	AAAACGAACTAACTAATGTTTAAGTAAAAGAA
pAGT572_Nemo_for	GTAATAAAAGTATCAACAAAAAATTGTTAATATACCTC
pGAP_for	GGTTTCTCCTGACCCAAAGACTTTAAA
pCat1_for	TAAGCTGTAGACCCAGCACTTCAAT
tGAP_rev	TCATTATGGCTGTATCTACTTTAGCGTA

Supplementary Table 2 Overview of utilised strains for cloning and protein production purposes

Overview of all bacterial (*E. coli*) and yeast (*S. cerevisiae* and *P. pastoris*) strains that have been utilised within this study.

Descriptor	Organism	Genotype	Purpose	Source
**DH10B**	*Escherichia coli*	F^- mcrA Δ(*mrr-hsd*RMS-*mcr*BC) Φ80d*lac*ZΔM15 Δ*lac*X74 *end*A1 *rec*A1 *deo*R Δ(*ara,leu*)7697 *ara*D139 *gal*U *gal*K *nup*G *rps*L λ^-	Cloning and plasmid propagation	ThermoFisher Scientific (Waltham, US)
**BL21(DE3)**	*Escherichia coli*	*B dcm ompT hsdS* ($r_B^- m_B^-$) *gal*	sfGFP 1-10 production	ThermoFisher Scientific (Waltham, US)
**INV*Sc*1**	*Saccharomyces cerevisiae*	*MATa his3D1 leu2 trp1-289 ura3-52 MAT his3D1 leu2 trp1-289 ura3-52*	Protein production	ThermoFisher Scientific (Waltham, US)
**X-33**	*Pichia pastoris*	wild type<br>mut^+ phenotype	Protein production	ThermoFisher Scientific (Waltham, US)

Supplementary Table 3 Overview of all created and utilised plasmids within this study

Overview of all exploited and created plasmids within this study. All plasmids are part of the Yeast Secrete and Detect Kit which is available through the non-profit plasmid depository Addgene (Kit # 1000000166).

name	insert	purpose	Creation	Addgene entry
pAGM9121	lacZ selection marker	universal Level 0 module cloning	Sylvestre Marillonnet, previous work	#51833
pAGM22082_cRed	canthaxanthin selection marker	Level 2 episomal *E. coli* expression plasmid	Pascal Püllmann, previous work	#117225
pAGM22082_sfGFP1-10	superfolder GFP 1-10 variant	production of sfGFP 1-10 for split GFP assay	this study	#153515
pAGT572_Nemo	lacZ selection marker	episomal protein production in *S. cerevisiae*	this study	#153487
pPAP001	lacZ selection marker	episomal protein production in *P. pastoris*	this study	#153488
pPAP002	lacZ selection marker	episomal protein production in *P. pastoris*	this study	#153489
pPAP003	lacZ selection marker	Genomic integration plasmid for *P. pastoris*	this study	#153490
pAGM9121_TwinStrep-GFP11	TwinStrep-GFP11 tag	C-terminal tag for modular assembly	this study	#153514
pAGM9121_TwinStrep	TwinStrep tag	C-terminal tag for modular assembly	this study	#153513
pAGM9121_Strep II	Strep II tag	C-terminal tag for modular assembly	this study	#153512

pAGM9121_His2-GFP11	His2-GFP11 tag	C-terminal tag for modular assembly	this study	#153511
pAGM9121_TEV-His-GFP11	TEV-His-GFP11 tag	C-terminal tag for modular assembly	this study	#153510
pAGM9121_OctaHis-GFP11	OctaHis-GFP11 tag	C-terminal tag for modular assembly	this study	#153509
pAGM9121_GFP11	GFP11 tag	C-terminal tag for modular assembly	this study	#153508
pAGM9121_Sce-Prepro SP	*Sce*-Prepro signal peptide	signal peptide for modular assembly	this study	#153491
pAGM9121_Kma-Inulinase SP	*Kma*-Inulinase signal peptide	signal peptide for modular assembly	this study	#153492
pAGM9121_Sce-Invertase 2 SP	*Sce*-Invertase 2 signal peptide	signal peptide for modular assembly	this study	#153493
pAGM9121_Sce-Acid Phosphatase SP	*Sce*-Acid Phosphatase signal peptide	signal peptide for modular assembly	this study	#153494
pAGM9121_Gma-UPO SP	*Gma*-UPO signal peptide	signal peptide for modular assembly	this study	#153495
pAGM9121_Hsa-Serum Albumin SP	*Hsa*-Serum Albumin signal peptide	signal peptide for modular assembly	this study	#153496
pAGM9121_Aaw-Glucoamylase SP	*Aaw*-Glucoamylase signal peptide	signal peptide for modular assembly	this study	#153497
pAGM9121_Sce-Killer Protein SP	*Sce*-Killer Protein signal peptide	signal peptide for modular assembly	this study	#153498
pAGM9121_Mro-UPO SP	*Mro*-UPO signal peptide	signal peptide for modular assembly	this study	#153499

pAGM9121_Cfo-CPO SP	*Cfo*-CPO signal peptide	signal peptide for modular assembly	this study	#153500
pAGM9121_Cgl-UPO SP	*Cgl*-UPO signal peptide	signal peptide for modular assembly	this study	#153501
pAGM9121_Cci-UPO SP	*Cci*-UPO signal peptide	signal peptide for modular assembly	this study	#153502
pAGM9121_Ani- α Amylase SP	*Ani*-α Amylase signal peptide	signal peptide for modular assembly	this study	#153503
pAGM9121_Sce- α Galactosidase SP	*Sce*-α Galactosidase signal peptide	signal peptide for modular assembly	this study	#153504
pAGM9121_Gga-Lysozym SP	*Gga*-Lysozym signal peptide	signal peptide for modular assembly	this study	#153505
pAGM9121_Aae-UPO* SP	*Aae*-UPO* signal peptide	signal peptide for modular assembly	this study	#153506
pAGM9121_Mth-UPO SP	*Mth*-UPO signal peptide	signal peptide for modular assembly	this study	#153507

Supplementary Table 4 Employed signal peptides, their origins and amino acid sequences

Overview and background information on all exploited signal peptides within this study.

Naturally secreted protein	***Kingdom***	***Genus***	***Species***	***Abbreviation***	***Sequence***
Mating pheromone alpha factor	Fungi	Yeast	*Saccharomyces cerevisiae*	*Sce*–Prepro	**MRFPSIFTAVLFAASSALAAPVNTTTEDETAQIPAEAVIGYLDLEGDFDVAVLPFSNSTNNGLLF INTTIASIAAKEEGVSLDKREA**
Inulinase	Fungi	Yeast	*Kluyveromyces marxianus*	*Kma*–Inulinase	**MKLAYSLLLPLAGVSAA**
Invertase 2	Fungi	Yeast	*Saccharomyces cerevisiae*	*Sce*–Invertase2	**MLLQAFLFLLAGFAAKISA**
Acid Phosphatase 5	Fungi	Yeast	*Saccharomyces cerevisiae*	*Sce*–Acid Phosphatase	**MFKSVVYSILAASLANAA**
Gma UPO	Fungi	Basidiomy cete	*Galerina marginata*	*Gma*–UPO	**MRGTPIFASLIALFAHAAIAFPAYGSLAGLTREQLDEILPTLEIRA**
Serum Albumin	Animal	Homo	*Homo sapiens*	*Hsa*–Serum Albumin	**MKWVTFISLLFLFSSAYSA**
Glucoamylase	Fungi	Ascomyce te	*Aspergillus awamori*	*Aaw*–Glucoamylase	**MSFRSLLALSGLVCSGLA**
Killer Protein K1 Toxin	Fungi	Yeast	*Saccharomyces cerevisiae*	*Sce*–Killer Protein	**MTKPTQVLVRSVSILFFITLLHLVVA**
Mro UPO	Fungi	Basidiomy cete	*Marasmius rotula*	*Mro*–UPO	**MKLAISSSLIALVSVTTALANSQDVVDFGA**
Cfo CPO	Fungi	Ascomyce te	*Caldariomyces fumago*	*Cfo*–CPO	**MFSKVLPFVGAVAALPHSVRA**
Cgl UPO	Fungi	Ascomyce te	*Chaetomium globosum*	*Cgl*–UPO	**MRTSLLPALAAVSPVLA**
Cci UPO	Fungi	Basidiomy cete	*Coprinopsis cinerea*	*Cci*–UPO	**MISTSKHLFVLLPLFLVSHLSLVLGFPAYASLGGLTERQVEEYTSKLPIVA**

α Amylase	Fungi	Ascomycete	*Aspergillus niger*	*Ani*–α Amylase	**MVAWWSLFLYGLQVAAPALA**
α Galactosidase	Fungi	Yeast	*Saccharomyces cerevisiae*	*Sce*–α Galactosidase	**MFAFYFLTACISLKGVFGA**
Lysozyme g2	Animal	Bird	*Gallus gallus*	*Gga*–Lysozym	**MLGKNDPMCLVLVLLGLTALLGICQGA**
Aae UPO (variant PaDa-I)*	Fungi	Basidiomycete	*Agrocybe aegerita*	*Aae*–UPO*	**MKYFPLFPTLVYAVGVVAFPDYASLAGLSQQELDAIIPTLEARA**
Mth UPO	Fungi	Ascomycete	*Myceliophthora thermophila*	*Mth*–UPO	**MRASVLPVLIAISPALA**

Supplementary Table 5 Employed C-terminal Tags, their amino acid sequence and purpose

Overview and sequence of all employed C-terminal protein tags constructed and utilised within this study. All sequences are terminated by introduction of a stop codon (*), terminating the open reading frame of tripartite signal peptide-gene-C-terminal Tag constructs.

Name	*Sequence*	*Purpose*
GFP11	SDGGSGGGSTSRDHMVLHEYVNAAGIT*	Protein detection (split GFP)
OctaHis-GFP11	SGGSGGGHHHHHHHHDGGSGGGSTSRDHMVLHEYVNAAGIT*	Protein purification (His Tag)<br>Protein detection (split GFP)
TEV-His-GFP11	SGSENLYFEGMGSHHHHHHSGMSDGGSGGGSTSRDHMVLHEYVNAAGIT*	Protein purification (His Tag)<br>Protein detection (split GFP)
His2-GFP11	SGGSGGGHHHHHHGGSGGSHHHHHHDGGSGGGSTSRDHMVLHEYVNAAGIT*	Protein purification (His Tag)<br>Protein detection (split GFP)
Strep II	SGGSAWSHPQFEK*	Protein purification (Strep Tag)
TwinStrep	SGGSAWSHPQFEKGGGSGGGSGGSAWSHPQFEK*	Protein purification (Strep Tag)
TwinStrep-GFP11	SGGSAWSHPQFEKGGGSGGGSGGSAWSHPQFEKDGGSGGGSTSRDHMVLHEYVNAAGIT*	Protein detection (split GFP)<br>Protein purification (Strep Tag)

Supplementary Table 6 Protein identification by mass spectroscopy

Analysis summary of the different unspecific peroxygenases produced in *Saccharomyces cerevisiae* by tryptic protein digest and MS analysis.

Protein Name	*SUM PEP Score*	*Sequence Coverage (%)*	*MW (kDa)†*	*calc. pI†*
*Aae*UPO*	283.796	55	46.6	5.92
*Gma*UPO	155.372	56	43.6	6.18
*Mro*UPO	379.202	76	34.4	5.91
*Mwe*UPO	215.479	65	34.3	5.91
*Cgl*UPO	34.652	27	35.5	5.58
*Mth*UPO	359.253	62	35.6	6.35
*Tte*UPO	271.531	57	42.0	5.86

†including the respective, attached signal peptide

Supplementary Table 7 Measurement parameters for achiral and chiral GC-MS

Summary of measurement parameters of achiral and chiral GC-MS analysis depending on the respective substrate for conversion

Substrate	GC-MS	Column	Products	Internal Standard	Temperature program
Napthalene	Achiral	SH-Rxi-5Sil MS	1,4-Naphthoquinone (*m/z* 158)<br>1-Naphthol (*m/z* 144)	Ethyl benzoate (*m/z* 150)	50 °C<br>9 °C/min to 190 °C<br>55 °C/min to 300 °C hold 2 min
Styrene	Chiral	Lipodex E	Styrene oxide (*m/z* 119)	Ethyl benzoate (*m/z* 150)	80 °C hold 30 min<br>50 °C/min to 200 °C hold 5 min
Phenylethane	Chiral	Lipodex E	Phenylethyl alcohol (*m/z* 122)	Ethyl benzoate (*m/z* 150)	70 °C hold 40 min<br>10 °C/min to 170 °C<br>50 °C/min to 200 °C hold 5 min
Phenylpropane	Chiral	Lipodex E	1-Phenyl-1-propanol (*m/z* 136)	Ethyl benzoate (*m/z* 150)	70 °C hold 35 min<br>5 °C/min to 110 °C<br>100 °C/min to 200 °C hold 5 min
Phenylbutane	Chiral	Lipodex E	1-Phenyl-1-butanol (*m/z* 150)	Ethyl benzoate (*m/z* 150)	80 °C hold 40 min<br>1 °C/min to 110 °C<br>100 °C/min to 200 °C hold 5 min
Phenylpropane	Chiral	Lipodex E	1-Phenyl-1-pentanol (*m/z* 164)	Ethyl benzoate (*m/z* 150)	90 °C hold 60 min<br>1 °C/min to 110 °C<br>100 °C/min to 200 °C hold 5 min
N-phenethyl phthalimide	Achiral	OPTIMA 5MS Accent	*N*-(2-hydroxy-2-phenylethyl)phthalimide<br>*N*-Phthaloyl-2-oxo-phenylethyl amine	Ethyl benzoate (*m/z* 150)	50°C<br>10°C/min to 300°C hold 3 min

Supplementary Table 8 Protein coverage of the protein digest and MS analysis

Sequence coverage of the primary UPO sequences by tryptic protein digest and subsequent peptide analysis.

Signal Peptide

NDEAHP – Detected peptide fragments

PAP230120_1 *Aae*UPO*

MRGTPIFASL IALFAHAAIA FPAYGSLAGL TREQLDEILP TLEIR**AEPGL PPGPLENSSA KLVNDEAHPW KPLRPGDIRG PCPGLNTLAS HGYLPR**NGVA TPAQIINAVQ EGFNFDNQAA IFATYAAHLV DGN**LITDLLS IGR**K**TRLTGP DPPPPASVGG LNEHGTFEGD ASMTRGDAFF GNNH**DFNETL F**EQLVDYSNR** FGGGKYNLTV AGELRFK**RIQ DSIATNPNFS FVDFR**FFTAY **GETTFPANLF VDGRRDDGQL DMDAARSFFQ FSRMPDDFFR** APSPR**SGTGV EVVVQAHPMQ PGR**NVGKINS YTVDPTSSDF STPCLMYEKF VNITVK**SLYP NPTVQLRKAL NTNLDFLFQG VAA**GCTQVFP YGR**DSGGSAW SHPQ**FEK**GGG SGGGSGGSAW SHPQ**FEKDGG SGGGSTSRDH MVLHEYVNAA GIT

PAP230120_2 *Gma*UPO

MVAWWSLFLY GLQVAAPALA **EPAKPPGPLK** DTSAKLVNDK **AHTWKPLTPT DIRGPCPGLN TLASHG**WLPR NGIASPSEII TAVQEGFNMD NSLAIF**VTYA AHLVDGNILT DK**LSIGGK**TA LTGPNPPAPA IVGGLNTHAV FEGDTSMTR**G DFFFGNNHDF NETLF**DEFVD FSNR**FGGGKY NLTVAGEFRW QR**IQDSIATN PNFSFVSPR**Y FTAY**AESTFP INFFIDGRQN DGQLNLTVAR** GFFQN**SRMPD GFHR**ANGTRG TEGIDVIAEA HPIEPGSNVG GVNNYVVDPT SADFNTFCLL YENFVNKTIK **GLYPNPTGAL RKALNTNLGF FFSGISDTGC TQVFPYGKGS GGSAWSHPQF EKGGGSGGGS GGSAWSHPQF EK**DGGSGGGS TSR**DHMVLHE YVNAAGIT**

PAP230120_3 *Mro*UPO

MKLAISSSLI ALVSVTTA**LA NSQDVVDFGA SAHPWK**APGP NDSR**GPCPGL NTLANHGFLP R**NGRNISVPM IVK**AGFEGYN VQSDILILAG KIGMLTSREA DTISLEDLKL HGTIEHDASL SRED**VAIGDN LHFNEAIFTT LANSNPGADV YNISSAAQVQ H**DRLADSLAR NPNVTNTDLT ATIRSSESAF FLTVMSAGDP LRGEAPKKFV NVFFR**EER**MP IKEGWKRSTT PITIPLLGPI IERITELSDW KPTGDNCGAI VLSPELSGGS AWSHPQFEKG GGSGGGSGGS AWSHPQFEKD GGSGGGSTSR DHMVLHEYVN AAGIT**

PAP230129_4 *Mwe*UPO

MKLAISSSLI ALVSVTTALA **NSQDVVDFGA SAHPWK**APGP NDSR**GPCPGL NTLANHGFLP R**NGRNISVPM IVK**AGFEGYN VQSDVLITAG K**VGMLTSR**EA DTISLEDLKL HGTIEHDASL SR**EDAAIGDN LHFNEAIFTT LANSNPGADV YNISSAAQVQ H**DRLADSLAR** NPNVTNTDVT ATIR**ASESAF YLTVMSAGDP LRGEAPKKFV NVCFR**EERMP VKEGWKR**STT PINIPLLVPI IERIIELSDW KPTGDNCGAI VLSPDLSGGS AWSHPQFEKG GGSGGGSGGS AWSHPQFEKD GGSGGGSTSR DHMVLHEYVN AAGIT**

PAP230120_5 *Cgl*UPO

MVAWWSLFLY GLQVAAPALA GFDTWAPPGP YDVRGPCPML NTLTNHGFFP HDGQDIDRET TENALFDALH VNKTLASFLF DFALTTNPIA NSTTFSLNDL GNHNVLEHDA SLSRADAYHG SVLAFNHTIF EETK**SYWTDE TVTLK**MAADA RYYRIKSSQA TNPTYQMSEL GDAFTYGESA AYVVLFGDKE SQTVPRSWVE WLFEK**EQLPQ HLGWKRPATS FELNDLDKFM ALIQNYTQEI EEPSCESR**KQ RRKPRGPSHF GFSGGSAWSH PQFEK**GGGSG GGSGGSAWSH PQFEK**DGGSG GGSTSR**DHMV LHEYVNAAGI T**

PAP230110_6 *Mth*UPO

MFAFYFLTAC ISLKGVFG**AG FDTWSPPGPY DVRAPCPMLN TLANHGFLPH DGKDITR**EQT ENALFEALHI NKTLASF**LFD FALTTNPK**NT STF**SLNDLGN HNILEHDASL SR**ADAYFGNV LQFNQTVFDE TK**TYWEGDTI DLR**MAAKARL GRIK**TSQATN PTYSMSELGD AFTYGESAAY VVVLGDKESR** TVKRSWVEWF FEH**EQLPQHL GWKRPAASFE EEDLNSSMEE IEK**YTKELEG SNSTSGSQKH RRRLPRR**RAH FGFSGGSAWS HPQFEKGGGS GGGSGGSAWS HPQFEK**DGGS GGGSTSR**DHM VLHEYVNAAG IT**

PAP230120_7 *Tte*UPO

MRFPSIFTAV LFAASSALAA PVNTTTEDET AQIPAEAVIG YLDLEGDFDV AVLPFSNSTN NGLLFINTTI ASIA**AKEEGV SLDKRE**AGFD **SWHPPAPGDR RGPCPMLNTL ANHGFLPHNG R**NITK**EITVN ALNSALNVNK** TLGELLFNF**A VTTNPQPNAT FFDLDHLSRH NILEHDASLS R**ADYYFGHDD HTFNQTVFDQ TKSYWK**TPII DVQQAANAR**L ARVLTSNATN PTFVLSQIGE AF**SFGETAAY ILALGDR**VSG TVP**RQWVEYL FENERLPLEL GWR**R**AKEVIS NSDLDQLTNR VINATGALAN ITR**KIKVRDF HAGR**FPGEGS GGSAWSHPQF EKGGGSGGGS GGSAWSHPQF EK**DGGSGGGS TSR**DHMVLHE YVNAAGIT**

Supplementary Table 9 Enantiomeric excess determination for UPO catalysed *N*-Phthaloyl-phenylethyl amine conversion by chiral HPLC analysis

Overview of measurement results for *N*-Phthaloyl-phenylethyl amine conversion by chiral HPLC analysis.

Enzyme	Retention time S-Enantiomer [min]	Retention time R-Enantiomer [min]	Area S [mAU*s]	Area R [mAU*s]	ee
*Cgl*UPO	13.45	18.22	7.294E04	477.713	98.7 %
*Tte*UPO	12.91	16.76	33.33	18.73	28.0 %
*Mth*UPO	13.45	17.75	2683.68945	19.33951	98.6 %

Supplementary Table 10 Overview of protein sequences and sequence alignments

Overview of protein sequences of all utilised peroxygenases differentiated by their occurrence as long- or short-type UPO. Sequence identity comparison analysis conducted with Geneious R10 (Biomatters Limited) depicting % of aa identity.

Protein sequences

A: Signal Peptide- Gene overhang amino acid (A**GCA**)

S: Gene- C-terminal Tag overhang amino acid (T**TCG**)

Long-type UPOs:

Aae*UPO (330 amino acids)

AEPGLPPGPLENSSAKLVNDEAHPWKPLRPGDIRGPCPGLNTLASHGYLPRNGVATPAQIINAVQEGFNFDNQAAI
FATYAAHLVDGNLITDLLSIGRKTRLTGPDPPPPASVGGLNEHGTFEGDASMTRGDAFFGNNHDFNETLFEQLVDY
SNRFGGGKYNLTVAGELRFKRIQDSIATNPNFSFVDFRFFTAYGETTFPANLFVDGRRDDGQLDMDAARSFFQFSR
MPDDFFRAPSPRSGTGVEVVVQAHPMQPGRNVGKINSYTVDPTSSDFSTPCLMYEKFVNITVKSLYPNPTVQLRKA
LNTNLDFLFQGVAAGCTQVFPYGRD**S**

***Gma*UPO** (331 amino acids)

AEPAKPPGPLKDTSAKLVNDKAHTWKPLTPTDIRGPCPGLNTLASHGWLPRNGIASPSEIITAVQEGFNMDNSLAIF
VTYAAHLVDGNILTDKLSIGGKTALTGPNPPAPAIVGGLNTHAVFEGDTSMTRGDFFFGNNHDFNETLFDEFVDFS
NRFGGGKYNLTVAGEFRWQRIQDSIATNPNFSFVSPRYFTAYAESTFPINFFIDGRQNDGQLNLTVARGFFQNSRM
PDGFHRANGTRGTEGIDVIAEAHPIEPGSNVGGVNNYVVDPTSADFNTFCLLYENFVNKTIKGLYPNPTGALRKALN
TNLGFFFSGISDTGCTQVFPYGKG**S**

***Cci*UPO** (339 amino acids)

AFPPPPPEPIKDPWLKLVNDRAHPWRPLRRGDVRGPCPGLNTLASHGYLPRDGVATPAQIITAVQEGFNMEYGIAT
FVTYAAHLVDGNPLTNLISIGGKTRKTGPDPPPPAIVGGLNTHAVFEGDASMTRGDFHLGDNFNFNQTLWEQFKD
YSNRYGGGRYNLTAAAELRWARIQQSMATNGQFDFTSPRYFTAYAESVFPINFFTDGRLFTSNTTAPGPDMDSALS
FFRDHRYPKDFHRAPVPSGARGLDVVAAAYPIQPGYNADGKVNNYVLDPTSADFTKFCLLYENFVLKTVKGLYPNP
KGFLRKALETNLEYFYQSFPGSGGCPQVFPWGKSD**S**

Short-type UPOs:

***Mro*UPO** (238 amino acids)

ASAHPWKAPGPNDSRGPCPGLNTLANHGFLPRNGRNISVPMIVKAGFEGYNVQSDILILAGKIGMLTSREADTISLE
DLKLHGTIEHDASLSREDVAIGDNLHFNEAIFTTLANSNPGADVYNISSAAQVQHDRLADSLARNPNVTNTDLTATI
RSSESAFFLTVMSAGDPLRGEAPKKFVNVFFREERMPIKEGWKRSTTPITIPLLGPIIERITELSDWKPTGDNCGAIVLS
PEL**S**

***Mwe*UPO** (238 amino acids)

ASAHPWKAPGPNDSRGPCPGLNTLANHGFLPRNGRNISVPMIVKAGFEGYNVQSDVLITAGKVGMLTSREADTIS
LEDLKLHGTIEHDASLSREDAAIGDNLHFNEAIFTTLANSNPGADVYNISSAAQVQHDRLADSLARNPNVTNTDVTA
TIRASESAFYLTVMSAGDPLRGEAPKKFVNVCFREERMPVKEGWKRSTTPINIPLLVPIIERIIELSDWKPTGDNCGAI
VLSPDL**S**

***Cgl*UPO** (244 amino acids)

AGFDTWAPPGPYDVRGPCPMLNTLTNHGFFPHDGQDIDRETTENALFDALHVNKTLASFLFDFALTTNPIANSTTF
SLNDLGNHNVLEHDASLSRADAYHGSVLAFNHTIFEETKSYWTDETVTLKMAADARYYRIKSSQATNPTYQMSELG
DAFTYGESAAYVVLFGDKESQTVPRSWVEWLFEKEQLPQHLGWKRPATSFELNDLDKFMALIQNYTQEIEEPSCES
RKQRRKPRGPSHFGF**S**

***Mth*UPO** (246 amino acids)

AGFDTWSPPGPYDVRAPCPMLNTLANHGFLPHDGKDITREQTENALFEALHINKTLASFLFDFALTTNPKNTSTFSL
NDLGNHNILEHDASLSRADAYFGNVLQFNQTVFDETKTYWEGDTIDLRMAAKARLGRIKTSQATNPTYSMSELGD
AFTYGESAAYVVVLGDKESRTVKRSWVEWFFEHEQLPQHLGWKRPAASFEEEDLNSSMEEIEKYTKELEGSNSTSG
SQKHRRRLPRRRAHFGF**S**

***Tte*UPO** (244 amino acids)

AGFDSWHPPAPGDRRGPCPMLNTLANHGFLPHNGRNITKEITVNALNSALNVNKTLGELLFNFAVTTNPQPNATF
FDLDHLSRHNILEHDASLSRADYYFGHDDHTFNQTVFDQTKSYWKTPIIDVQQAANARLARVLTSNATNPTFVLSQI
GEAFSFGETAAYILALGDRVSGTVPRQWVEYLFENERLPLELGWRRAKEVISNSDLDQLTNRVINATGALANITRKIK
VRDFHAGRFPGEG**S**

Sequence identity comparison

	PaDa-I	GmaUPO	CciUPO	MroUPO	MweUPO	CglUPO	MthUPO	TteUPO
PaDa-I		71.4%	61.8%	30.1%	30.1%	22.6%	24.5%	24.8%
GmaUPO	71.4%		64.5%	28.6%	28.2%	22.2%	24.9%	24.1%
CciUPO	61.8%	64.5%		25.8%	25.0%	22.1%	22.6%	23.6%
MroUPO	30.1%	28.6%	25.8%		94.5%	30.4%	31.0%	31.0%
MweUPO	30.1%	28.2%	25.0%	94.5%		30.8%	31.0%	31.4%
CglUPO	22.6%	22.2%	22.1%	30.4%	30.8%		72.1%	49.8%
MthUPO	24.5%	24.9%	22.6%	31.0%	31.0%	72.1%		51.8%
TteUPO	24.8%	24.1%	23.6%	31.0%	31.4%	49.8%	51.8%	

III. NMR spectra

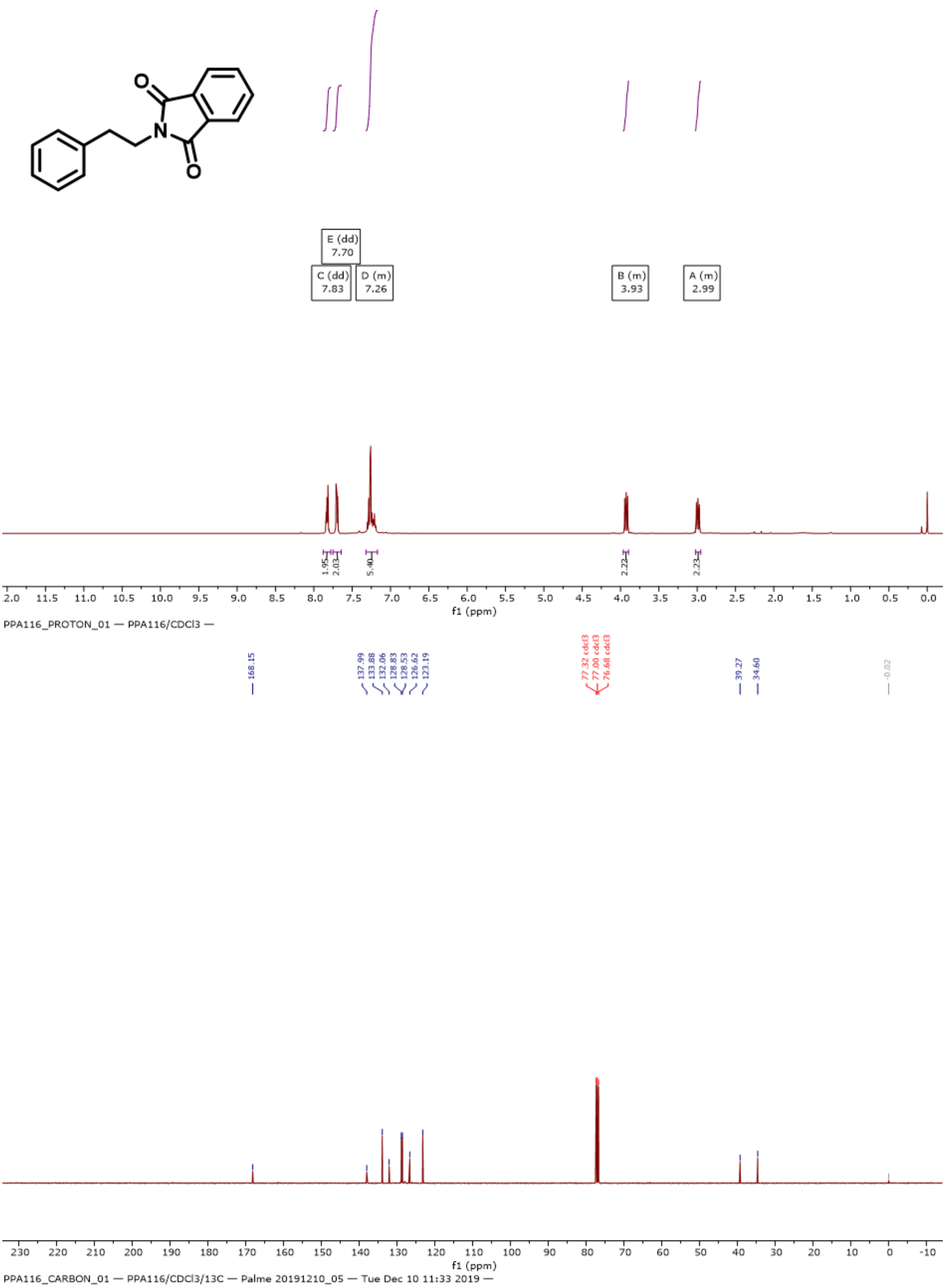

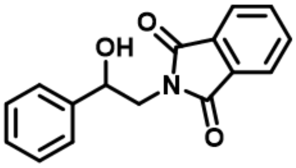

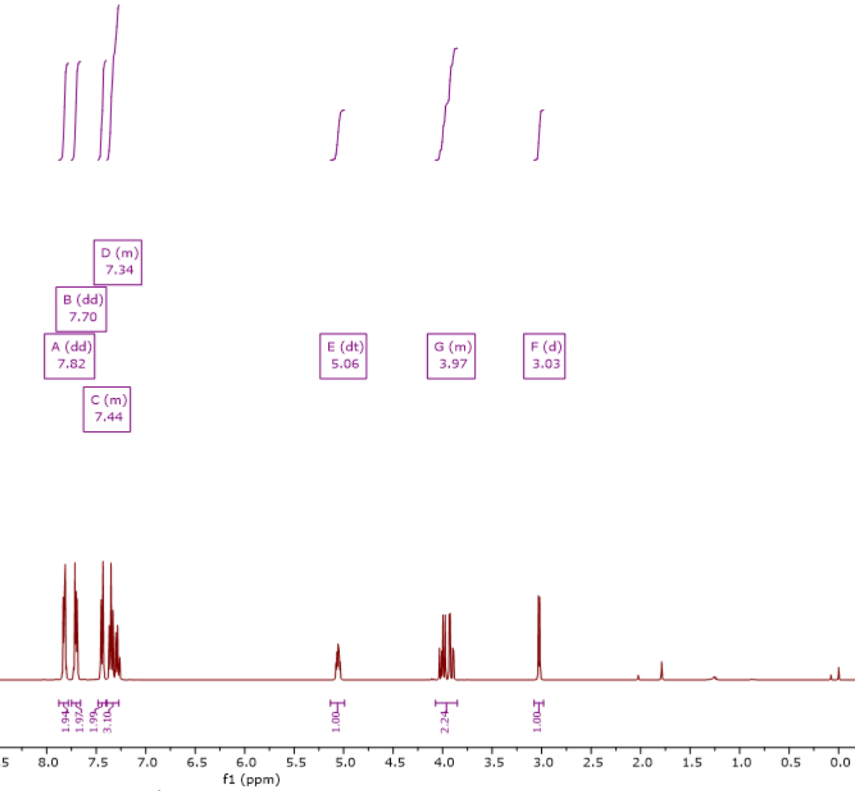

PPA120_2_PROTON_01 — PPA120_2/CDCl3/1H — Palme 20200108_03 — Wed Jan 8 15:55 2020 —

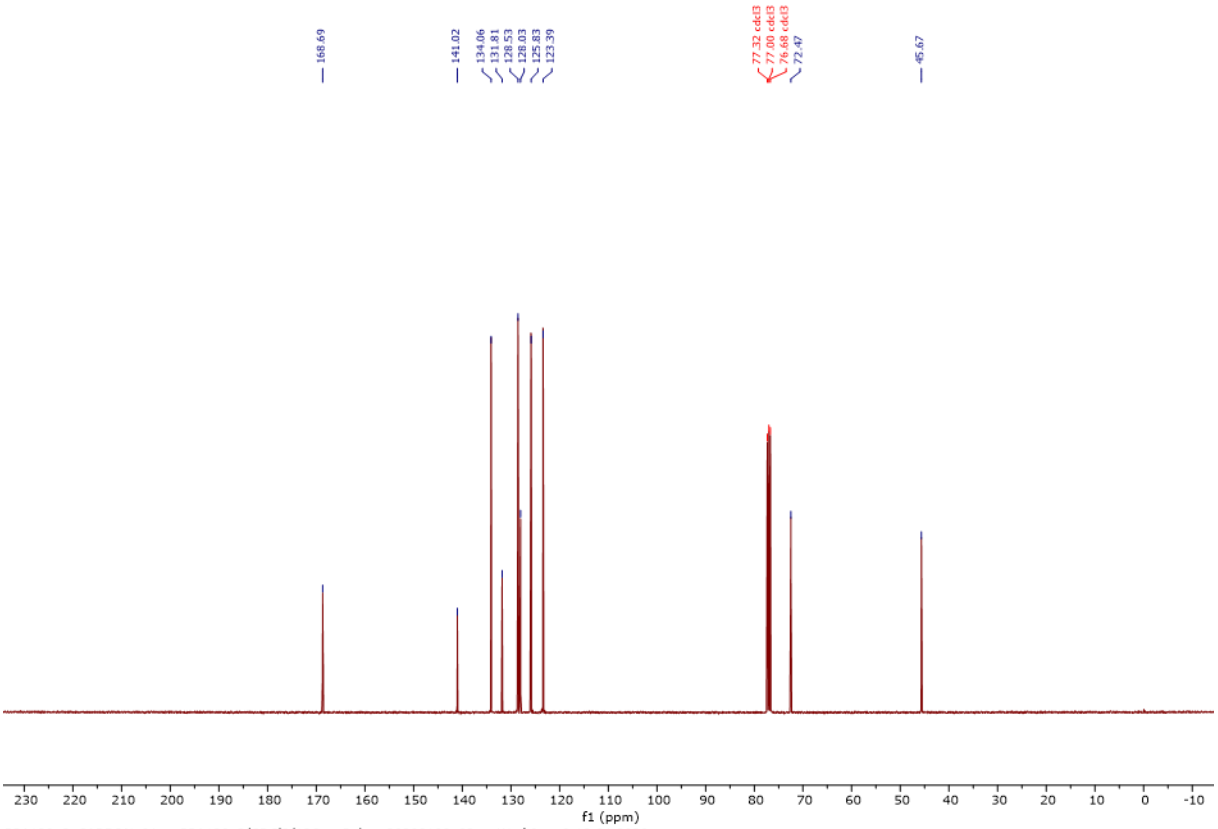

PPA120_2_CARBON_01 — PPA120_2/CDCl3/13C — Palme 20200108_03 — Wed Jan 8 18:01 2020 —

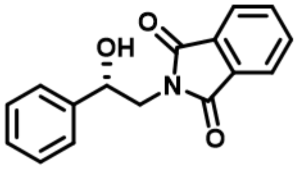

by chemical conversion

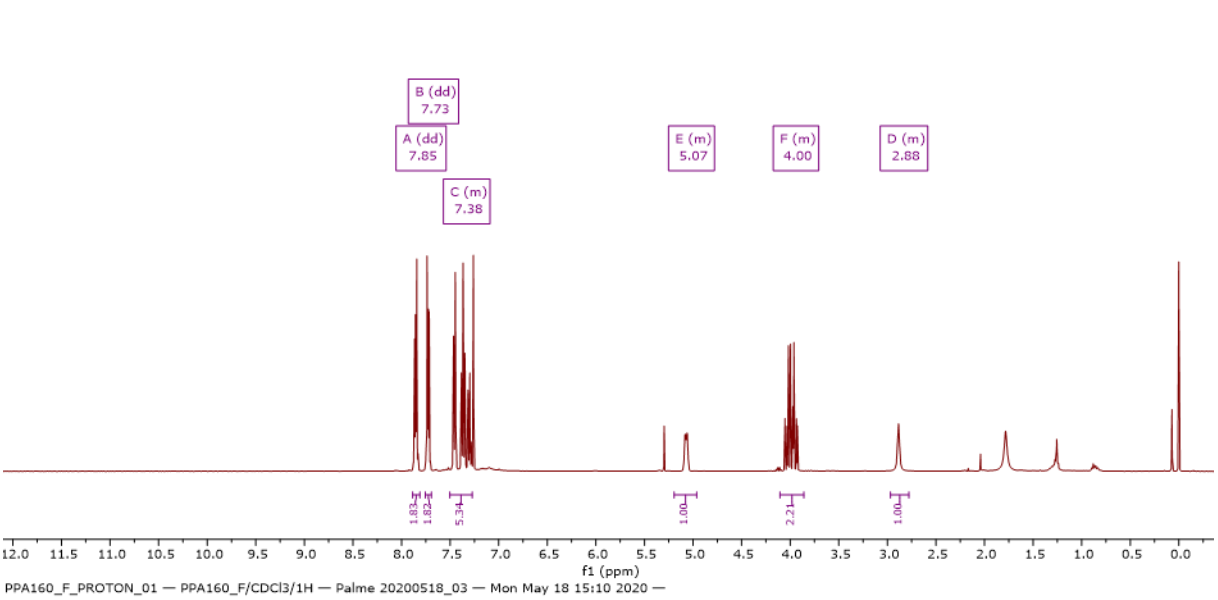

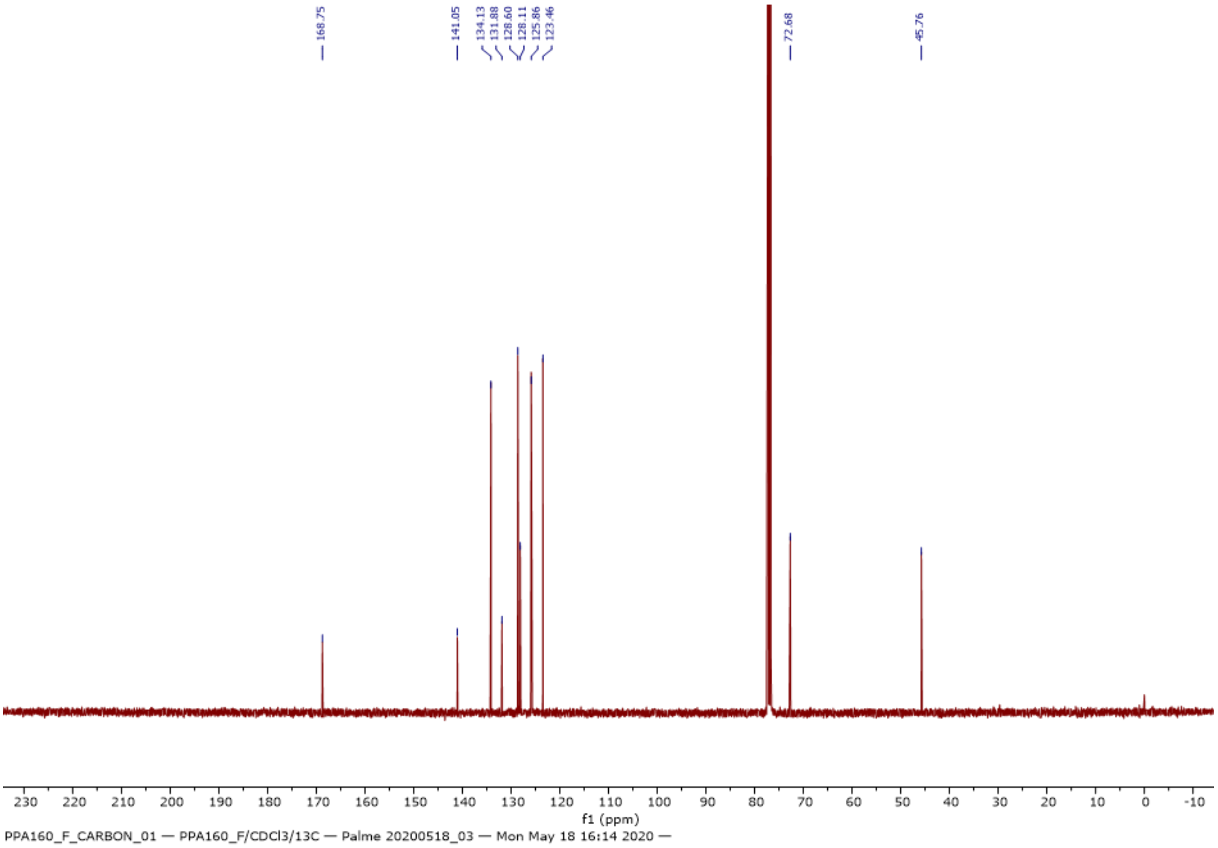

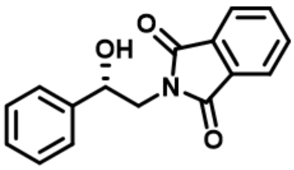

by enzymatic conversion

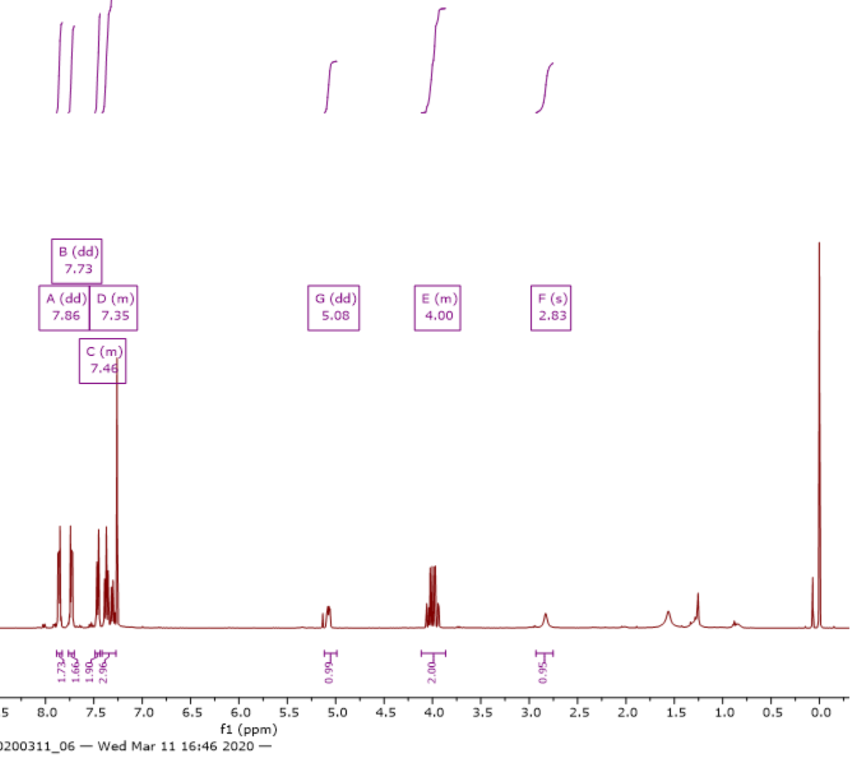

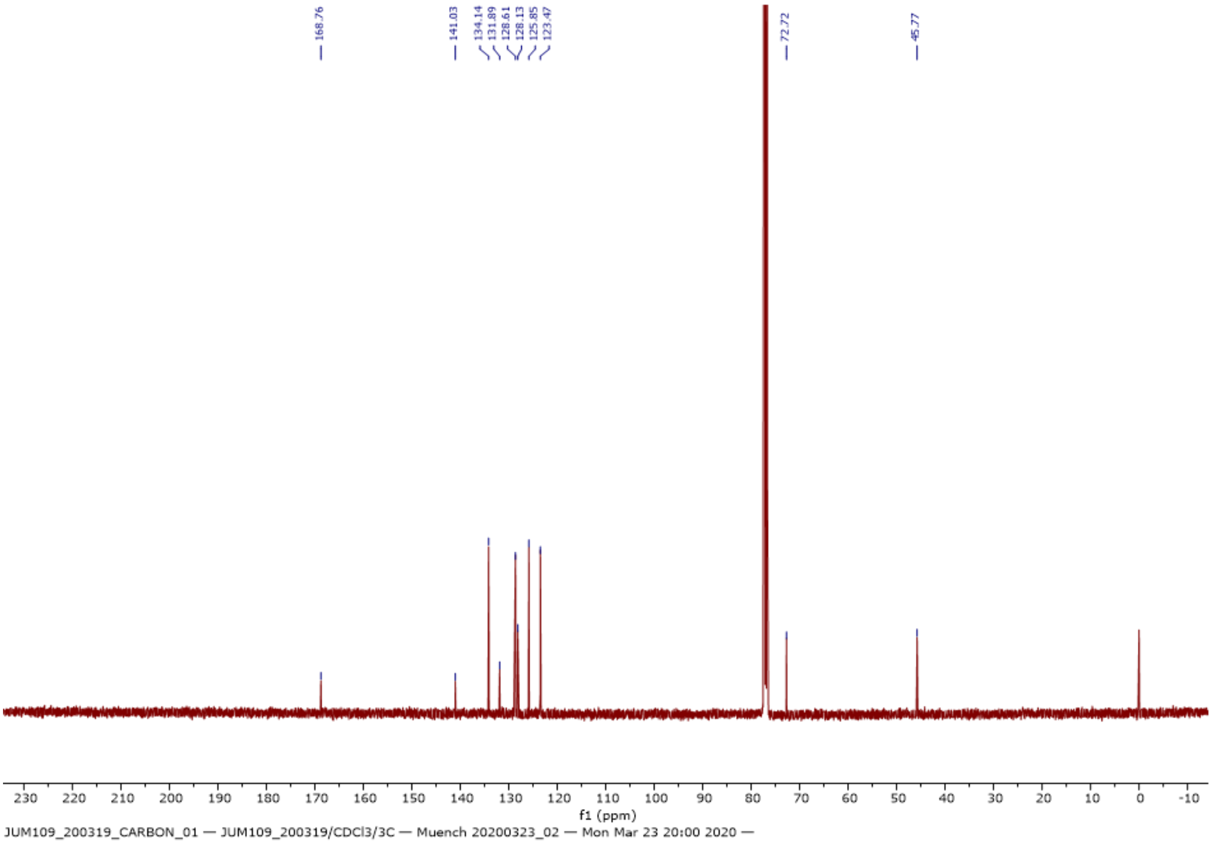

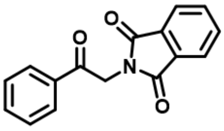

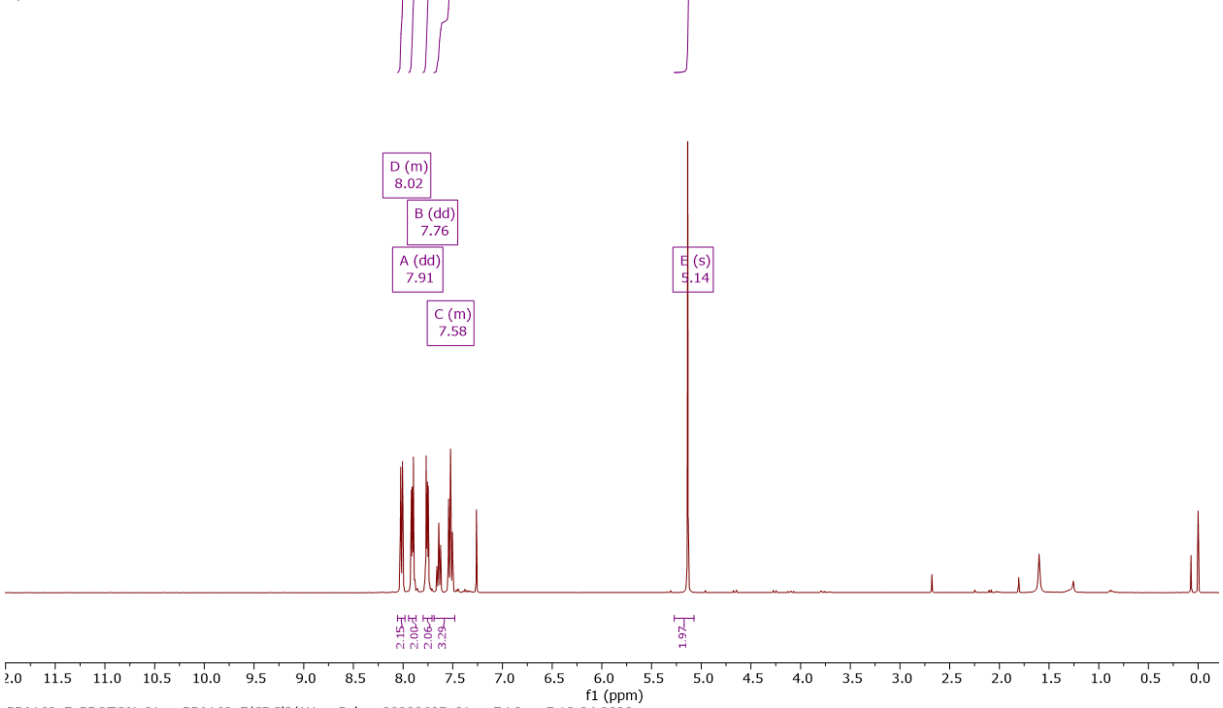

PPA162_F_PROTON_01 — PPA162_F/CDCl3/1H — Palme 20200605_01 — Fri Jun 5 13:34 2020 —

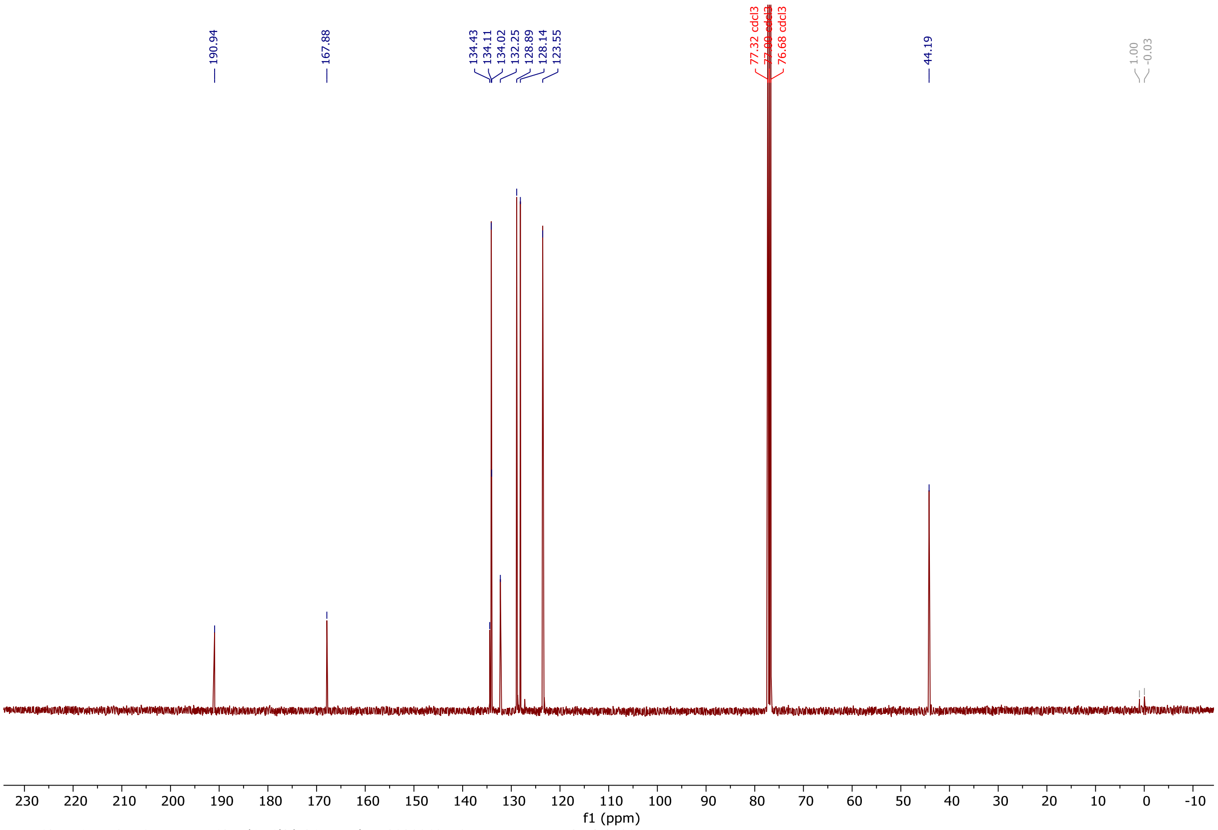

PPA162_F_CARBON_01 — PPA162_F/CDCl3/13C — Palme 20200605_01 — Fri Jun 5 13:58 2020 —
